# A Versatile Room-Temperature Route to Di- and Trisubstituted Allenes Using Flow-Generated Diazo Compounds**

**DOI:** 10.1002/anie.201501538

**Published:** 2015-05-26

**Authors:** Jian-Siang Poh, Duc N Tran, Claudio Battilocchio, Joel M Hawkins, Steven V Ley

**Affiliations:** Innovative Technology Centre, Department of ChemistryUniversity of Cambridge, Lensfield Road, Cambridge CB2 1EW (UK); Pfizer Worldwide Research and DevelopmentEastern Point Road, Groton, CT 06340 (USA)

**Keywords:** allenes, carbenes, copper, diazo compounds, flow chemistry

## Abstract

A copper-catalyzed coupling reaction between flow-generated unstabilized diazo compounds and terminal alkynes provides di- and trisubstituted allenes. This extremely mild and rapid transformation is highly tolerant of several functional groups.

The use of machine-based enabling technologies can accelerate the chemical discovery process and improve research efficiency.[1] Likewise, flow chemistry methods can play a useful role by assisting the adoption of new synthesis concepts.[2] Nevertheless, any new technology will be disruptive and eventual incorporation must be done with considerable care and forethought to ensure full integration with existing practices.

We, as well as others,[3] have been interested in exploiting the dynamics of flow reactor systems to generate wider chemical reactivity windows. For example, the generation of unstabilized diazo compounds in flow has proven to be an attractive method, as these reactive intermediates can be combined directly with a variety of substrates under mild reaction conditions.[3a,b]

Allenes are interesting functional groups owing to their orthogonal π bonds. Several natural bioactive molecules, as well as synthetic materials, possess this particular structural motif.[4] These building blocks are used for a wide range of transformations, in particular those producing heterocyclic architectures, for example, furans and pyrrolidines.[5] Direct access to allenes is possible by various protocols,[6] however, harsh reaction conditions are often required. In the particular case of accessing trisubstituted allenes by direct coupling reactions, these are scarce and proceed with very low functional-group tolerance.[6a,e,f] We report herein a new practical method to generate functionalized di- and trisubstituted allenes using flow-generated unstabilized diazo compounds at room temperature.

Copper(I) is known to be a suitable catalyst for the coupling between diazo compounds and terminal alkynes. We anticipated that by generating unstabilized diazo compounds in flow, we could perform this coupling reaction under much milder reaction conditions. Following the mechanism described by Wang et al.,[6a] the reaction is expected to be initiated by the formation of the copper acetylide species **5** from the copper(I) catalyst and terminal acetylene **3** in the presence of a base (Scheme 1). The copper acetylide intermediate can then be intercepted by the diazo compound **2** to generate the carbene copper complex **6**. A 1,2-carbon migration followed by a 1,3-copper migration would then form the vinyl copper species **8**. A final protonation of this intermediate should provide the allene **4** and regenerate the active copper(I) catalyst species for the next catalytic cycle.

**Scheme 1:**
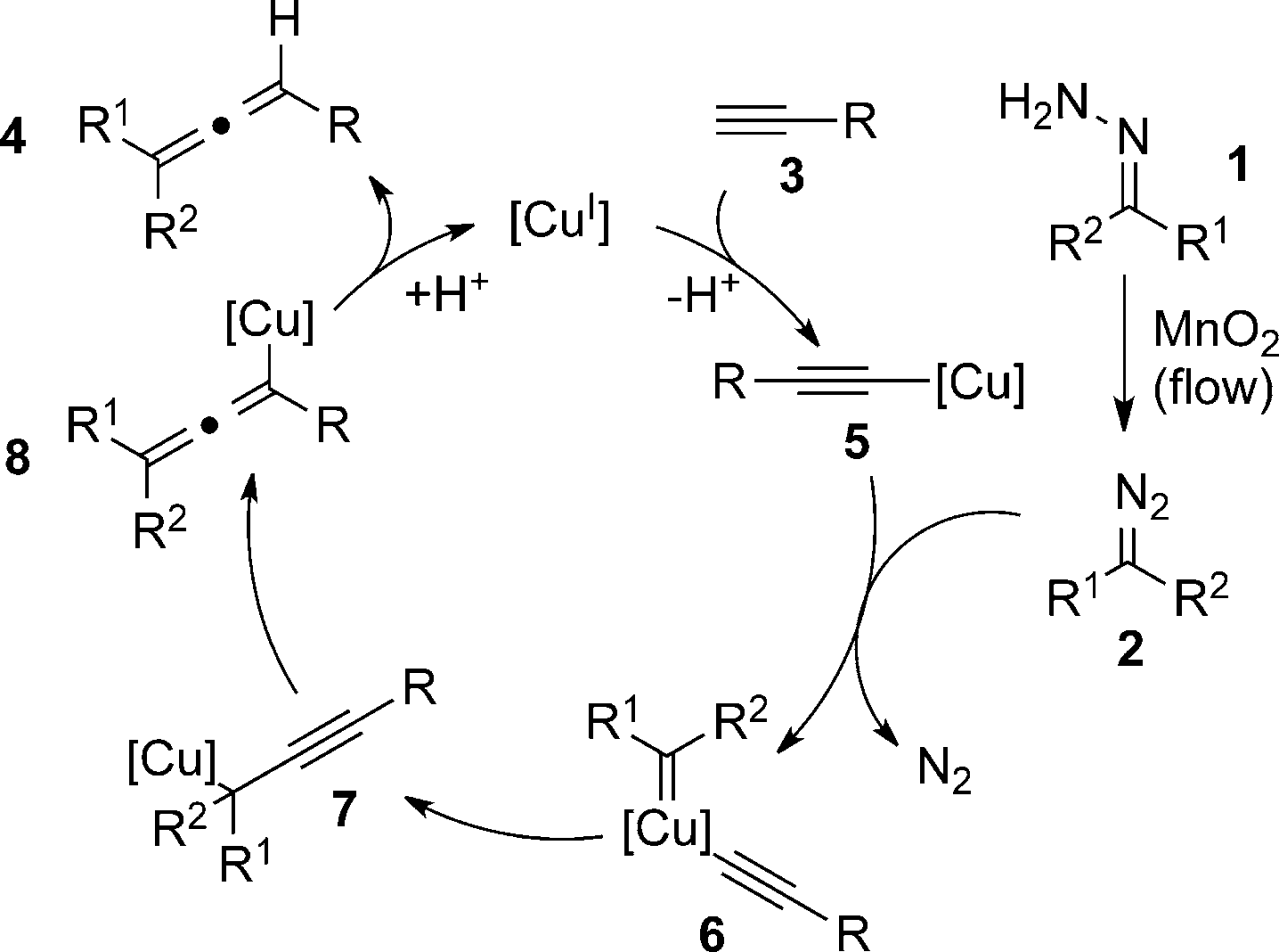
Proposed reaction mechanism of coupling between diazo compounds and terminal alkynes catalyzed by copper(I).

The reaction was initially optimized using (4-chlorobenzylidene)hydrazine (**1 a**)[7] and propargyl alcohol (**3 a**) as model substrates (Scheme 2). The hydrazone **1 a** was oxidized in flow to generate the diazo compound **2 a**. This latter compound was immediately quenched by adding it to a reaction mixture containing **3 a**, the catalyst, and the base. Our initial screening showed the importance of the counterion of the copper complex in determining the reaction outcome. For instance, the use of [Cu(acac)_2_], CuF_2_, CuOAc, CuCl, and CuTC (copper thiophene-2-carboxylate) resulted in less than 5 % yield of the desired allene product **4 a**. The main by-products detected resulted from the degradation of the diazo compound along with unreacted **3 a**. Only CuI provided the desired allene **4 a** in high yield without the use of additional ligands. Under the optimized reaction conditions, a solution of **1 a** (0.1 m in CH_2_Cl_2_, DIPEA 2 equiv) was passed through a packed column reactor containing activated MnO_2_ (0.86 g) at a 0.5 mL min^−1^ flow rate.[3a,^8^] The output solution of the diazo species **2 a** was added (over 6 min) directly into a vial containing a mixture of CuI (10 mol %, 0.02 mmol), Et_3_N (0.4 mmol), and **3 a** (0.2 mmol) in 1,4-dioxane (2 mL). The reaction mixture was stirred at room temperature for another 10 minutes and **4 a** was obtained in 91 % yield after filtration over Celite and purification.[9] Notably, in contrast with previous observations using stabilized diazo compounds (i.e. possessing adjacent ester groups), we did not detect any trace of the acetylene coupled by-product **4 a′**.[6b,^10^] Moreover, the reaction could be carried out either under argon or even exposed to air, with no change in the product yield.

**Scheme 2:**
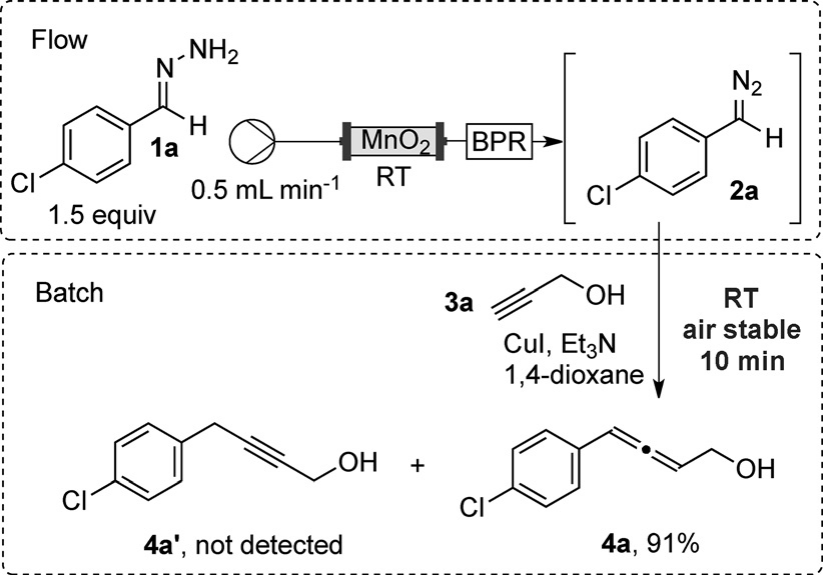
Design of reaction flow with model substrates.

We then investigated different diazo compounds and alkynes to assess the reliability and robustness of the protocol (Table 1). In most cases, the reactions were completed in very high yield using only 1.5 equivalents of the hydrazone (ca. 1.1 equiv of diazo compound).[8] Electronic properties of the aromatic rings did not greatly influence the reaction outcomes as shown with the allenes **4 a**–**g** (yields ranging from 81–99 %). A vinyl diazo compound provided the conjugated allene **4 h** in acceptable yield (48 %). We then studied the effect of alkyne substituents. A broad range of sensitive functional groups were found to be compatible with the transformation, including bromides, iodides, cyanides, acetals, and ferrocene. Moreover, the mild reaction conditions allow the use of volatile alkynes in the syntheses corresponding to the allenes **4 j**–**l** and **4 x**. Interestingly, free hydroxy and monoprotected amines were tolerated despite their potential to undergo copper-catalyzed O=H and N=H carbene insertion.[11] Highly sensitive functional groups such as epoxides were also tolerated, thus giving the allene **4 v** in 89 % yield without any degradation. Aryl alkynes participated smoothly in the reaction conditions, thus giving the allenes **4 m** and **4 w** in very high yields. Vinyl alkynes reacted with slightly lower efficiency owing to the instability of the conjugated system in the allene product **4 o**. No diastereoselectivity was observed when racemic substituted propargyl alcohols were used (**4 s** and **4 t**).

**Table 1:** Coupling products from diazo compounds and terminal alkynes.[a]

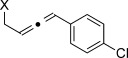	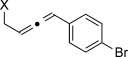	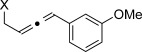
X=OH, **4 a**, 91 % X=NHTs, **4 d**, 93 %	X=OH, **4 b**, 99 % X=NHTs, **4 e**, 92 %	X=OH, **4 c**, 93 % X=NHTs, **4 f**, 81 %
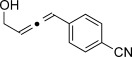	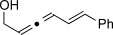	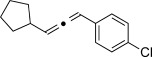
**4 g**, 83 %	**4 h**, 48 %^b^	**4 i**, 88 %
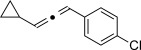	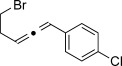	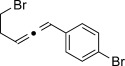
**4 j**, 99 %	**4 k**, 82 %	**4 l**, 93 %
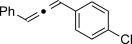	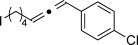	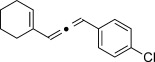
**4 m**, 92 %	**4 n**, 75 %	**4 o**, 63 %
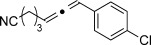	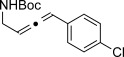	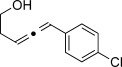
**4 p**, 93 %	**4 q**, 93 %	**4 r**, 90 %
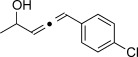	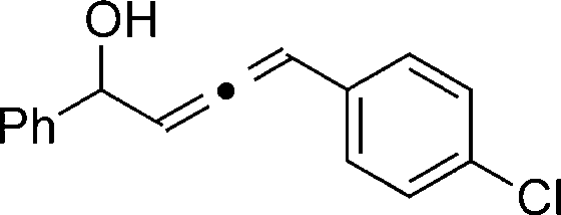	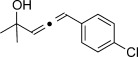
**4 s**, 83 % (1:1 d.r.)	**4 t**, 97 % (1:1 d.r.)	**4 u**, 82 %
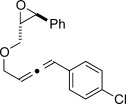	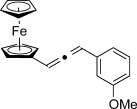	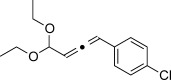
**4 v**, 89 % (1:1 d.r.)	**4 w**, 92 %	**4 x**, 99 %

[a] Standard reaction conditions: 0.2 mmol of the alkyne **3**, 0.3 mmol of the hydrazone **1**, 0.02 mmol of CuI, and 0.4 mmol of TEA in 2 mL 1,4-dioxane at RT; for more detail see the Supporting Information. The yield is that of the isolated product. The d.r. values were determined by NMR analysis of the crude reaction mixture. [b]>20:1 *E*/*Z* ratio. Boc=*tert*-butoxycarbonyl, TEA=triethylamine, Ts=4-toluenesulfonyl.

Additional evidence for the high level of selectivity and functional-group compatibility of this protocol was demonstrated with the successful modification of polysubstituted compounds (Scheme 3). Norethindrone, a drug molecule currently used in contraceptive pills, reacted under standard reaction conditions with **2 a** to provide a 63 % yield of **9** as a mixture of diastereoisomers (separable) in a 1.5:1 ratio. Interestingly, the Michael acceptor present in the molecule remained intact during the reaction of the terminal alkyne with the diazo species. High yield was also observed with the propargylated quinine **10**, again highlighting the versatility of our protocol.

**Scheme 3:**
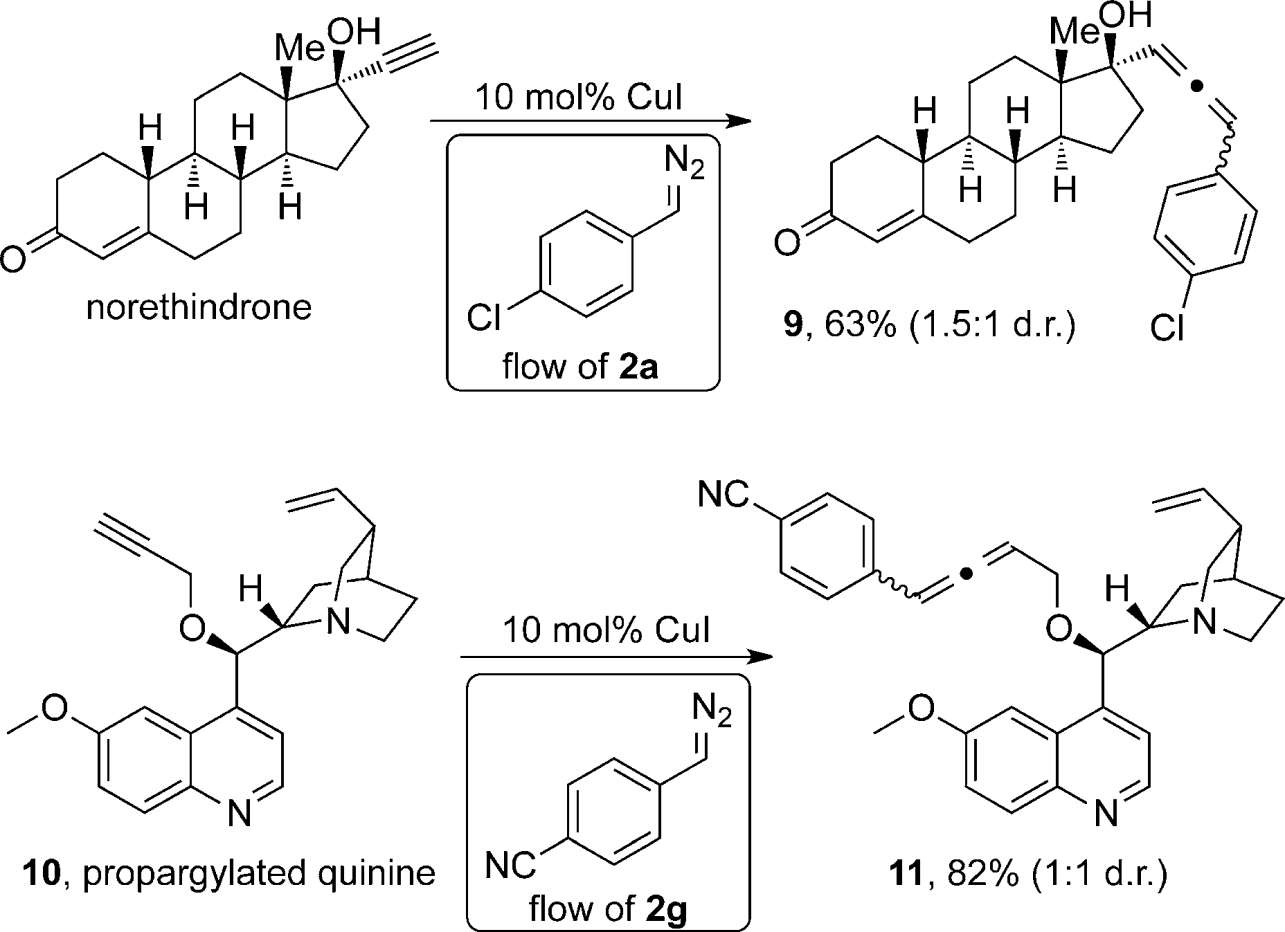
Late-stage modification of norethindrone and propargylated quinine.

Trisubstituted allenes are particularly challenging synthetic targets owing to the steric hindrance generated during the formation of the copper carbene species.[6a] For example, when applying our standard reaction conditions to ketones, dimerization of the corresponding diazo compound was mainly observed. Pleasingly, the use of 2,6-lutidine (20 mol %) as an additive gave very good yields of the trisubstituted allenes. By using this improved protocol, a similar level of group tolerance was achieved with yields ranging from 53–99 % (Table 2). Diaryl ketones were compatible, thus providing the corresponding allenes **12 h**–**j** in good yield. Thiophene was also tolerated to yield the allene **12 g** in 53 %. We were also able to access more complex structures (**12 k** and **12 l**), thus clearly demonstrating the novelty of our approach. Additionally, in an attempt to produce larger quantities of **12 f**, we were able to successfully obtain 0.88 g (73 %) of material within just 30 minutes of reaction time. Most importantly, at no point in the process did we detect any build-up of reactive diazo species.[12] It is important to ensure that the diazo species does not build-up if proceeding beyond small laboratory scale since these compounds can be highly energetic.

**Table 2:** Trisubstituted allenes obtained from coupling reaction between diazo compounds and terminal alkynes.[a]

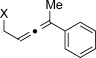	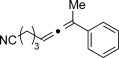	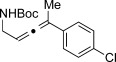
X=NHTs, **12 a**, 67 % X=NHBoc, **12 b**, 74 % X=OMe, **12 c**, 65 %	**12 d**, 73 %	**12 e**, 89 %
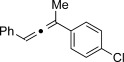	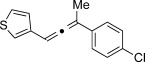	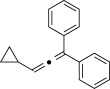
**12 f**, 99 % (73 %)[b]	**12 g**, 53 %	**12 h**, 78 %[c]
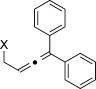	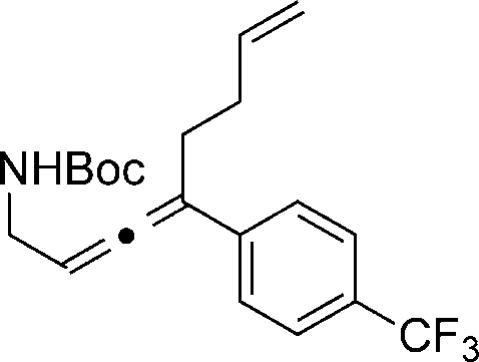	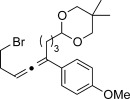
X=NHBoc, **12 i**, 87 %[c] X=OH, **12 j**, 80 %[c]	**12 k**, 58 %	**12 l**, 55 %

[a] Standard reaction conditions: 0.2 mmol of the alkyne **3**, 0.3 mmol of the hydrazone **1**, 0.02 mmol of CuI, 0.04 mmol of 2,6-lutidine, and 0.4 mmol of TEA in 2 mL 1,4-dioxane at RT; for more detail see the Supporting Information. The yield is that of the isolated product.

[b] Yield on 5 mmol scale, for more detail see the Supporting Information.

[c] 2.0 equiv of hydrazone were used.

We have also carried out a preliminary mechanistic study using deuterated methanol as an additive under standard reaction conditions (Scheme 4). This resulted in 37 % deuterium incorporation at the C3 position of the allene [D]-**4 j**, thereby providing some evidence for the vinylic copper species **8 j** as an intermediate.[13]

**Scheme 4:**
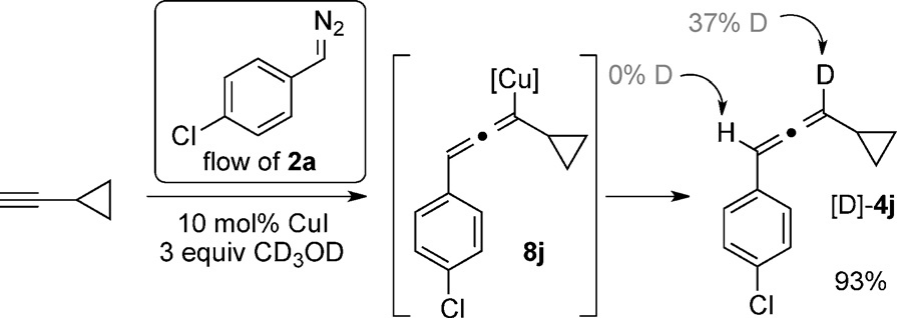
Trapping of the vinyl cuprate 8 j intermediate with CD_3_OD.

In summary, we have reported a useful method for the generation of highly substituted allenes. This approach results in a mild and practical preparation of allenes with a high degree of functional-group tolerance. The method illustrates further the advantages that accrue by amalgamating flow and batch methods of synthesis. Studies toward an asymmetric synthesis of allenes are currently ongoing in our laboratory.
