# Supplementary material for: A Versatile Room-Temperature Route to Di- and Trisubstituted Allenes Using Flow-Generated Diazo Compounds
Source: Angew Chem Int Ed Engl. 2015 May 26;54(27):7920–3. doi: 10.1002/anie.201501538 (PMC4515080; doi:10.1002/anie.201501538)
Supplement: Supplementary file 1 [file anie0054-7920-sd1.pdf]

Angewandte Chemie
Eine Zeitschrift der Gesellschaft Deutscher Chemiker

Supporting Information

A Versatile Room-Temperature Route to Di- and Trisubstituted Allenes Using Flow-Generated Diazo Compounds**

*Jian-Siang Poh, Duc N. Tran, Claudio Battilocchio, Joel M. Hawkins, and Steven V. Ley**

anie_201501538_sm_miscellaneous_information.pdf

Contents

1. General experimental details 2
2. Synthetic procedures and characterisation for starting materials 3
   2.1. Hydrazone synthesis 3
   2.2. Terminal alkyne synthesis 6
3. Synthetic procedures and characterisation for allenes 9
4. Deuteration experiment 32
5. NMR spectra 33
6. References 76

1. General experimental details

All batch reactions were performed using oven-dried glassware (200 °C) under an atmosphere of air unless otherwise stated. All flow reactions were performed using a Uniqsis FlowSyn platform.[1] In-line IR spectroscopy was performed using a Mettler Toledo FlowIR® device equipped with a SiComp (silicon) head.[2] Solvents were freshly distilled over calcium hydride and lithium aluminium hydride (THF or Et_2O) or calcium hydride (methanol, CH_2Cl_2, hexane and EtOAc). Additional anhydrous solvents were obtained from commercial sources and used directly (DMF, 1,4-dioxane, 2,6-lutidine). DIPEA and Et_3N were freshly distilled over calcium hydride and stored over 4 Å molecular sieves. All reagents were obtained from commercial sources and used without further purification.

Flash column chromatography was performed using high-purity grade silica gel (Merck grade 9385) with a pore size 60 Å and 230–400 mesh particle size under air pressure. Analytical thin layer chromatography (TLC) was performed using silica gel 60 F_{254} pre-coated glass backed plates and visualized by ultraviolet radiation (254 nm) and/or potassium permanganate solution as appropriate.

^{1}H NMR spectra were recorded on a 400 MHz DPX-400 Dual Spectrometer or a 600 MHz Avance 600 BBI Spectrometer as indicated. Chemical shifts are reported in ppm with the resonance resulting from incomplete deuteration of the solvent as the internal standard ($CDCl_3$: 7.26 ppm). ^{13}C NMR spectra were recorded the same spectrometers with complete proton decoupling. Chemical shifts are reported in ppm with the solvent resonance as the internal standard ($^{13}CDCl_3$: 77.16 ppm, t). ^{19}F NMR spectra were recorded on a 376 MHz Avance III HD Spectrometer. Chemical shifts are reported in ppm with $CFCl_3$ as the external standard ($CFCl_3$: 0.00 ppm). Data are reported as follows: chemical shift δ/ppm, integration (^{1}H only), multiplicity (s = singlet, d = doublet, t = triplet, q = quartet, br = broad, m = multiplet or combinations thereof; ^{13}C signals are singlets unless otherwise stated), coupling constants *J* in Hz, assignment. Spectra are assigned as fully as possible, using ^{1}H-COSY, DEPT-135, HMQC and HMBC where appropriate to facilitate structural determination. Signals that cannot be unambiguously assigned are reported with all possible assignments separated by a slash (e.g. H1/H2) or descriptions of their environments (e.g. ArH, OH, NH, CH). Multiple signals arising from diastereotopic or (pseudo)axial/equatorial positions are suffixed alphabetically (e.g. H1a, H1b). Overlapping signals that cannot be resolved are reported with their assignments denoted in list format (e.g. H1, H2 and H3). ^{1}H NMR signals are reported to 2 decimal places and ^{13}C signals to 1 decimal place unless rounding would produce a value identical to another signal. In this case, an additional decimal place is reported for both signals concerned.

Infrared spectra were recorded neat as thin films on a Perkin-Elmer Spectrum One FTIR spectrometer and selected peaks are reported (s = strong, m = medium, w = weak, br = broad).

High resolution mass spectrometry (HRMS) was performed using positive electrospray ionisation (ESI+), on either a Waters Micromass LCT Premier spectrometer or performed by the Mass Spectrometry Service for the Chemistry Department at the University of Cambridge. All *m/z* values are reported to 4 decimal places and are within ± 5 ppm of theoretical values.

Melting points were collected using a Stanford Research Systems OptiMelt Automated Melting Point System using a gradient of 1.0 °C per min.

2. Synthetic procedures and characterisation for starting materials

2.1. Hydrazone synthesis

General procedure for aldehyde-derived hydrazone formation:

To a solution of aldehyde (20.0 mmol, 1.0 equiv.) in methanol (20 mL) was added hydrazine hydrate (1.2 mL, 24 mmol, 1.2 equiv.) and the mixture stirred at r.t. for 1 h. The mixture was then evaporated under reduced pressure to provide the desired hydrazone. The crude hydrazone was used for generation of the corresponding diazo compound without further purification.

General procedure for ketone-derived hydrazone formation:

To a solution of ketone (20.0 mmol, 1.0 equiv.) in methanol (20 mL) was added hydrazine hydrate (2.9 mL, 60.0 mmol, 3.0 equiv.) and the mixture stirred at 80 °C for 3 h in a sealed vial. The solvent was removed under reduced pressure and the residue diluted with water (25 mL) and CH_2Cl_2 (25 mL). The mixture was separated, the aqueous layer extracted with CH_2Cl_2 (3 × 25 mL) and the combined organic extracts were dried ($MgSO_4$), filtered and evaporated under reduced pressure to provide the desired hydrazone. The crude hydrazone was used for generation of the corresponding diazo compound without further purification.

(4-chlorobenzylidene)hydrazine (1a):

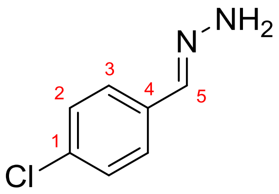

Isolated as an off-white amorphous solid (3.08 g, 19.9 mmol, 99%) following the general procedure for aldehyde-derived hydrazone formation. Data is consistent with a reported example.[3]

^{1}H NMR (600 MHz, $CDCl_3$): δ 7.69 (s, 1 H, H5), 7.47 (d, J = 8.5 Hz, 2 H, H3), 7.31 (d, J = 8.5 Hz, 2 H, H2), 5.54 (br s, 2 H, NH_2).
^{13}C NMR (150 MHz, $CDCl_3$): δ 141.8 (C5), 134.4 (C1), 133.7 (C4), 128.9 (C2), 127.4 (C3).
FTIR (ν_{max}, cm^{-1}): 3357 (w, NH_2), 3186 (w, NH_2), 1625 (w), 1594 (w), 1486 (w), 1393 (w), 1249 (w), 1218 (w), 1109 (w), 1090 (m), 1010 (w), 961 (w), 924 (w), 912 (w), 864 (w), 824 (s).

(4-bromobenzylidene)hydrazine:

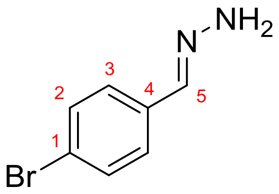

Isolated as an off-white amorphous solid (3.98 g, 20.0 mmol, 99%) following the general procedure for aldehyde-derived hydrazone formation. Data is consistent with a reported example.[4]

^{1}H NMR (600 MHz, CDCl$_3$): δ 7.67 (s, 1 H, H5), 7.47 (d, *J* = 8.5 Hz, 2 H, H2), 7.41 (d, *J* = 8.5 Hz, 2 H, H3), 5.56 (br s, 2 H, NH$_2$).
^{13}C NMR (150 MHz, CDCl$_3$): δ 141.7 (C5), 134.3 (C1), 131.9 (C2), 127.7 (C3), 122.4 (C4).
FTIR (ν_{max}, cm^{-1}): 3353 (w, NH$_2$), 3194 (w, NH$_2$), 1627 (w), 1586 (m), 1483 (w), 1391 (m), 1069 (s), 1006 (m), 924 (m), 912 (m), 862 (m), 818 (s).

(3-methoxybenzylidene)hydrazine:

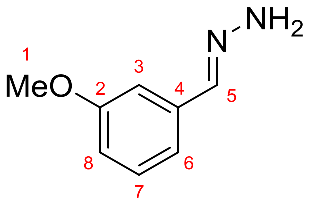

Isolated as a yellow oil (2.99 g, 19.9 mmol, 99%) following the general procedure for aldehyde-derived hydrazone formation.

^{1}H NMR (600 MHz, CDCl$_3$): δ 7.74 (s, 1 H, H5), 7.28 (dd, *J* = 8.4, 7.4 Hz, 1 H, H7), 7.18 (dd, *J* = 2.5, 0.8 Hz, 1 H, H3), 7.09 (dt, *J* = 7.4, 0.8 Hz, 1 H, H6), 6.88 (ddd, *J* = 8.4, 2.5, 0.8 Hz, 1 H, H8), 5.53 (br s, 2 H, NH$_2$), 3.84 (s, 3 H, H1).
^{13}C NMR (150 MHz, CDCl$_3$): δ 160.0 (C2), 143.1 (C5), 136.7 (C4), 129.7 (C7), 119.5 (C6), 115.4 (C8), 110.2 (C3), 55.4 (C1).
FTIR (ν_{max}, cm^{-1}): 3387 (w, NH$_2$), 3200 (w, NH$_2$), 2911 (w), 2835 (w), 1597 (s), 1575 (s), 1489 (m), 1466 (m), 1455 (m), 1431 (m), 1397 (w), 1317 (w), 1288 (m), 1263 (s), 1195 (w), 1155 (s), 1072 (w), 1038 (s), 994 (w), 967 (w), 922 (m), 862 (w).

4-(hydrazonomethyl)benzonitrile:

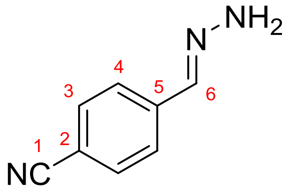

Isolated as a yellow amorphous solid (2.90 g, 20.0 mmol, 99%) following the general procedure for aldehyde-derived hydrazone formation. Data is consistent with a reported example.[5]

^{1}H NMR (600 MHz, CDCl$_3$): δ 7.70 (s, 1 H, H6), 7.62 (s, 4 H, H3 and H4), 5.81 (br s, 2 H, NH$_2$).
^{13}C NMR (150 MHz, CDCl$_3$): δ 139.9 (C6), 139.7 (C5), 132.5 (C3), 126.5 (C4), 119.1 (C1), 111.5 (C2).
FTIR (ν_{max}, cm^{-1}): 3377 (w, NH$_2$), 3198 (w, NH$_2$), 2221 (m, C≡N), 1589 (s), 1552 (m), 1503 (m), 1398 (m), 1244 (w), 1175 (w), 1091 (w), 909 (s), 869 (w), 824 (s).

((*E*)-3-phenylallylidene)hydrazine:

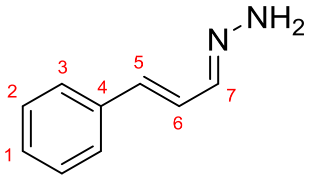

Isolated as a yellow waxy solid (2.91 g, 19.9 mmol, 99%) following the general procedure for aldehyde-derived hydrazone formation.

^{1}H NMR (600 MHz, $CDCl_3$): δ 7.55 (d, *J* = 9.1 Hz, 1 H, H7), 7.42 (d, *J* = 7.5 Hz, 2 H, H3), 7.33 (t, *J* = 7.5 Hz, 2 H, H2), 7.28 – 7.24 (m, 1 H, H1), 6.86 (dd, *J* = 16.1, 9.1 Hz, 1 H, H6), 6.66 (d, *J* = 16.1 Hz, 1 H, H5), 4.88 (br s, 2 H, NH_2).
^{13}C NMR (150 MHz, $CDCl_3$): δ 145.3 (C7), 136.6 (C4), 134.9 (C5), 128.8 (C2), 128.3 (C1), 126.7 (C3), 125.9 (C6).
FTIR (ν_{max}, cm^{-1}): 3322 (w, NH_2), 3177 (w, NH_2), 3025 (w), 2907 (w), 1640 (w), 1578 (w), 1485 (w), 1447 (m), 1383 (w), 1292 (w), 1257 (w), 1208 (w), 1178 (w), 1151 (w), 1127 (w), 1088 (m), 971 (s), 933 (w), 915 (w), 842 (w).

(1-phenylethylidene)hydrazine:

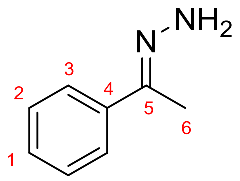

Isolated as a yellow oil (2.00 g, 14.9 mmol, 75%) following the general procedure for ketone-derived hydrazone formation. Data is consistent with a reported example.[3]

^{1}H NMR (600 MHz, $CDCl_3$): δ 7.67 – 7.63 (m, 2 H, H3), 7.38 – 7.33 (m, 2 H, H2), 7.32 – 7.28 (m, 1 H, H1), 5.36 (br s, 2 H, NH_2), 2.13 (s, 3 H, H6).
^{13}C NMR (150 MHz, $CDCl_3$): δ 147.4 (C5), 139.5 (C4), 128.4 (C2), 128.1 (C1), 125.6 (C3), 11.7 (C6).
FTIR (ν_{max}, cm^{-1}): 3385 (w, NH_2), 3214 (w, NH_2), 1592 (s), 1571 (w), 1495 (s), 1444 (s), 1369 (s), 1330 (w), 1254 (w), 1115 (m), 1079 (m), 1064 (m), 1026 (s), 951 (w), 914 (w).

(1-(4-chlorophenyl)ethylidene)hydrazine:

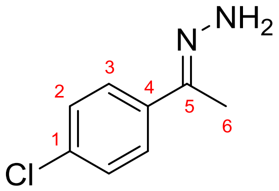

Isolated as an off-white crystalline solid (3.40 g, 20.0 mmol, 99%) following the general procedure for ketone-derived hydrazone formation; m.p. 48.5 – 49.5 °C (lit. m.p.[6] 48.4 – 50.1 °C). Data is consistent with reported examples.[3,6]

^{1}H NMR (600 MHz, $CDCl_3$): δ 7.57 (d, *J* = 8.6 Hz, 2 H, H3), 7.30 (d, *J* = 8.6 Hz, 2 H, H2), 5.38 (br s, 2 H, NH_2), 2.09 (s, 3 H, H6).
^{13}C NMR (150 MHz, $CDCl_3$): δ 146.0 (C5), 137.9 (C4), 133.9 (C1), 128.5 (C2), 126.8 (C3), 11.5 (C6).
FTIR (ν_{max}, cm^{-1}): 3356 (w, NH_2), 3215 (w, NH_2), 1638 (w), 1599 (w), 1488 (m), 1395 (w), 1369 (w), 1333 (w), 1270 (w), 1108 (w), 1092 (s), 1007 (m), 958 (w), 822 (s).

(1-(4-(trifluoromethyl)phenyl)pent-4-en-1-ylidene)hydrazine:

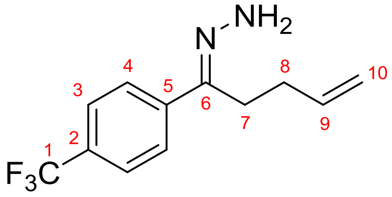

Isolated as a yellow waxy solid (4.24 g, 17.5 mmol, 88%) following the general procedure for ketone-derived hydrazone formation.

^{1}H NMR (600 MHz, $CDCl_3$): δ 7.74 (d, *J* = 8.2 Hz, 2 H, H4), 7.58 (d, *J* = 8.2 Hz, 2 H, H3), 5.86 (ddt, *J* = 17.0, 10.2, 6.7 Hz, 1 H, H9), 5.61 (br s, 2 H, NH_2), 5.11 (dq, *J* = 17.0, 1.4 Hz, 1 H, H10-trans), 5.05 (dd, *J* = 10.2, 1.4 Hz, 1 H, H10-cis), 2.74 – 2.68 (m, 2 H, H7), 2.33 – 2.27 (m, 2 H, H8).
^{13}C NMR (150 MHz, $CDCl_3$): δ 148.0 (q, *J* = 0.5 Hz, C6), 141.8 (q, *J* = 1.3 Hz, C5), 136.9 (C9), 129.8 (q, *J* = 32.4 Hz, C2), 125.8 (C4), 125.4 (q, *J* = 3.9 Hz, C3), 124.3 (q, *J* = 271.8 Hz, C1), 116.2 (C10), 29.2 (C8), 24.6 (C7).
^{19}F NMR (376 MHz, $CDCl_3$): -62.5 (s).
FTIR (ν_{max}, cm^{-1}): 3311 (w, NH_2), 3210 (w, NH_2), 1630 (w), 1524 (w), 1502 (w), 1408 (w), 1323 (s), 1161 (m), 1108 (s), 1067 (s), 1016 (m), 980 (w), 956 (w), 918 (w), 857 (m), 846 (m).

(4-(5,5-dimethyl-1,3-dioxan-2-yl)-1-(4-methoxyphenyl)butylidene)hydrazine:

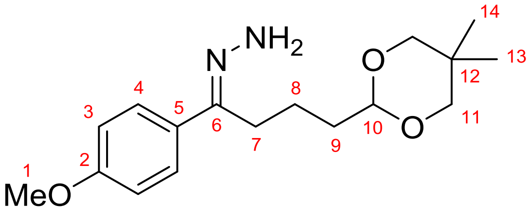

Isolated as a yellow viscous oil (1.53 g, 4.99 mmol, 99%) following the general procedure for ketone-derived hydrazone formation on a 5 mmol scale.

^{1}H NMR (600 MHz, $CDCl_3$): δ 7.57 (d, *J* = 9.0 Hz, 2 H, H4), 6.85 (d, *J* = 9.0 Hz, 2 H, H3), 5.40 (br s, 2 H, NH_2), 4.46 (t, *J* = 4.8 Hz, 1 H, H10), 3.80 (s, 3 H, H1), 3.59 (d, *J* = 11.2 Hz, 2 H, H11a), 3.40 (d, *J* = 11.2 Hz, 2 H, H11b), 2.66 – 2.59 (m, 2 H, H7), 1.75 – 1.64 (m, 4 H, H8 and H9), 1.17 (s, 3 H, H13/H14), 0.70 (s, 3 H, H13/H14).
^{13}C NMR (150 MHz, $CDCl_3$): δ 159.7 (C2), 150.7 (C6), 131.3 (C5), 127.0 (C4), 113.8 (C3), 101.9 (C10), 77.3 (C11), 55.4 (C1), 34.4 (C9), 30.3 (C12), 25.3 (C7), 23.1 (C13/C14), 21.9 (C13/C14), 20.0 (C8).
FTIR (ν_{max}, cm^{-1}): 3401 (w, NH_2), 3220 (w, NH_2), 2955 (w), 2839 (w), 1675 (w), 1603 (m), 1511 (s), 1464 (m), 1394 (m), 1363 (w), 1307 (m), 1247 (s), 1174 (m), 1131 (s), 1096 (m), 1033 (m), 1014 (m), 969 (w), 910 (m), 833 (m), 808 (w).

2.2. Terminal alkyne synthesis

2-phenyl-3-((prop-2-yn-1-yloxy)methyl)oxirane:

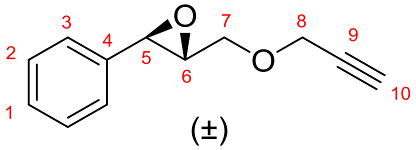

(±)

To a suspension of sodium hydride (88 mg, 2.40 mmol, 1.2 equiv., 60% dispersion in mineral oil) in anhydrous THF (5 mL) was added a solution of 3-phenylglycidol (300 mg, 2.00 mmol, 1.0 equiv.) in anhydrous THF (5 mL) slowly dropwise at 0 °C under an argon atmosphere. The mixture was stirred further at this temperature for 10 min. Propargyl bromide (0.18 mL, 2.40 mmol, 1.2 equiv.) was then added slowly dropwise at 0 °C and the mixture stirred further at r.t. for 1 h. The reaction mixture was quenched with a few drops of EtOAc and the solvent removed under reduced pressure. EtOAc (25 mL) was added to the residue and the organic layer washed with water (2 × 25 mL), brine (25 mL), dried ($MgSO_4$), filtered and evaporated under reduced pressure. The residue was purified by silica gel column chromatography (eluent: 10% EtOAc/hexane) to provide the title compound as a colourless oil (350 mg, 1.86 mmol, 93%). Data is consistent with a reported example.[7]

^{1}H NMR (600 MHz, $CDCl_3$): δ 7.37 – 7.33 (m, 2 H, H2), 7.33 – 7.29 (m, 1 H, H1), 7.29 – 7.26 (m, 2 H, H3), 4.26 (d, *J* = 2.4 Hz, 2 H, H8), 3.92 (dd, *J* = 11.4, 3.1 Hz, 1 H, H7a), 3.82 (dd, *J* = 6.3, 2.1 Hz, 1 H, H5), 3.70 (dd, *J* = 11.4, 5.3 Hz, 1 H, H7b), 3.24 (ddd, *J* = 5.3, 3.1, 2.1 Hz, 1 H, H6), 2.47 (t, *J* = 2.4 Hz, 1 H, H10).
^{13}C NMR (150 MHz, $CDCl_3$): δ 136.7 (C4), 128.6 (C2), 128.5 (C1), 125.8 (C3), 79.3 (C9), 75.2 (C10), 69.4 (C7), 60.9 (C6), 58.7 (C8), 56.0 (C5).
FTIR (ν_{max}, cm^{-1}): 3289 (w, alkyne CH), 2859 (w), 2118 (w, C≡C), 1743 (w), 1605 (w), 1498 (w), 1462 (w), 1443 (w), 1359 (w), 1243 (w), 1202 (w), 1097 (s), 1027 (w), 976 (w), 932 (w), 910 (w), 879 (m), 838 (w).
R_f = 0.25 (10% EtOAc/hexane).

(1*S*,2*S*,4*S*,5*R*)-2-((*R*)-(6-methoxyquinolin-4-yl)(prop-2-yn-1-yloxy)methyl)-5-vinyl-quinuclidine (10):

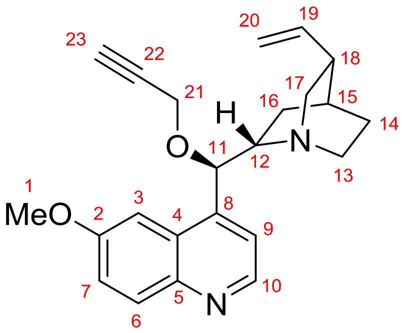

To a suspension of sodium hydride (0.40 g, 10.0 mmol, 2.0 equiv., 60% dispersion in mineral oil) in anhydrous DMF (15 mL) was added a solution of quinine (1.62 g, 5.0 mmol, 1.0 equiv.) in anhydrous DMF (15 mL) slowly dropwise at 0 °C under an argon atmosphere. The mixture was stirred further at this temperature for 10 min. Propargyl bromide (0.52 mL, 6.0 mmol, 1.2 equiv.) was then added slowly dropwise at 0 °C and the mixture stirred further at r.t. for 3 h. The reaction mixture was quenched with water (50 mL) and extracted with Et_2O (3 × 25 mL). The combined organic extracts were washed with water (4 × 50 mL), brine (50 mL), dried ($MgSO_4$), filtered and evaporated under reduced pressure. The residue was purified by silica gel column chromatography (eluent: 60% EtOAc/38% hexane/2% Et_3N) to provide the title compound **10** as an off-white amorphous solid (1.63 g, 4.5 mmol, 90%). Data is consistent with a reported example.[8]

^{1}H NMR (600 MHz, $CDCl_3$): δ 8.75 (d, *J* = 4.4 Hz, 1 H, H10), 8.03 (t, *J* = 5.9 Hz, 1 H, H6), 7.41 (d, *J* = 4.4 Hz, 1 H, H9), 7.39 – 7.34 (m, 2 H, H3 and H7), 5.83 – 5.68 (m, 1 H, H19), 5.31 (br s, 1 H, H11), 4.95 (dt, *J* = 17.1, 1.4 Hz, 1 H, H20-trans), 4.91 (dt, *J* = 10.3, 1.4 Hz, 1 H, H20-cis), 4.21 (dd, *J* = 15.9, 2.4 Hz, 1 H, H21a), 3.93 (s, 3 H, H1), 3.89 (dd, *J* = 15.9, 2.4

Hz, 1 H, H21b), 3.41 (br s, 1 H, H13a), 3.15 (br s, 1 H, H12), 3.07 (dd, J = 13.8, 10.1 Hz, 1 H, H17a), 2.72 – 2.62 (m, 1 H, H13b), 2.62 – 2.55 (m, 1 H, H17b), 2.45 (t, J = 2.4 Hz, 1 H, H23), 2.25 (br s, 1 H, H18), 1.82 – 1.79 (m, 1 H, H15), 1.79 – 1.71 (m, 2 H, H14a and H16a), 1.67 (br s, 1 H, H16b), 1.56 – 1.48 (m, 1 H, H14b).

^{13}C NMR (150 MHz, $CDCl_3$): δ 157.9 (C2), 147.7 (C10), 144.8 (C8), 143.9 (C5), 142.1 (C19), 132.0 (C6), 127.6 (C4), 121.9 (C7), 120.0 – 118.0 (br, C9), 114.3 (C20), 101.5 – 101.0 (br, C3), 79.4 (C22), 77.5 –76.7 (br, obscured by $CDCl_3$ peak, C11), 75.1 (C23), 60.1 (C12), 57.3 (C17), 56.3 (C21), 55.8 (C1), 43.2 (C13), 40.2 (C18), 27.87 (C14/C15), 27.86 (C14/C15), 23.5 – 22.5 (br, C16).

FTIR (ν_{max}, cm^{-1}): 2935 (m), 2099 (w, C≡C), 1620 (m), 1591 (w), 1506 (m), 1473 (m), 1455 (m), 1429 (m), 1362 (m), 1306 (w), 1240 (s), 1228 (s), 1107 (m), 1082 (s), 1050 (s), 1032 (s), 905 (m), 852 (m), 839 (m), 817 (s).

R_f = 0.20 (60% EtOAc/38% hexane/2% Et_3N).

3. Synthetic procedures and characterisation for allenes

General procedure for allene formation with aldehyde-derived hydrazones:

Conditioning phase: A solution of hydrazone (0.1 M) and DIPEA (0.2 M) in CH_2Cl_2 was passed through a column reactor (Omnifit® column, 6.6 mm i.d. × 50 mm length), packed with activated MnO_2 (0.86 g), at a flow rate of 0.5 mL min^{-1} for 20 min and the reactor output was monitored using a FlowIR® device. The flow was switched to solvent (DIPEA, 0.2 M in CH_2Cl_2) for 10 min. The column was then ready for the generation of the diazo compound.

Generation phase: A vial was charged with the appropriate alkyne (0.2 mmol, 1.0 equiv.), copper (I) iodide (3.9 mg, 0.02 mmol, 0.1 equiv.), 1,4-dioxane (2 mL) and Et_3N (0.05 mL, 0.4 mmol, 2 equiv.) and pre-mixed for 10 min. A solution of hydrazone (0.1 M) and DIPEA (0.2 M) in CH_2Cl_2 was passed through the pre-conditioned column reactor (Omnifit® column, 6.6 mm i.d. × 50 mm length), packed with activated MnO_2 (0.86 g), at a flow rate of 0.5 mL min^{-1}. When the FlowIR® showed that the intensity of the diazo peak was stable, 3 mL of the output (1.5 equiv. with respect to the hydrazone) was directly added into the reaction vial (over 6 min) containing the copper acetylide and the reaction mixture further stirred at r.t. for 10 min. The mixture was then filtered through a pad of Celite®, eluting with EtOAc, and the filtrate evaporated under reduced pressure. The residue was purified immediately by silica gel column chromatography to provide the desired di-substituted allene product.

Any excess diazo compound produced during the conditioning phase or the generation phase before steady-state was reached was gently quenched by directing the output of the flow reactor into a stirred suspension of copper (I) iodide (0.10 g) in MeOH (25 mL) (**Figure 1**).

General procedure for allene formation with ketone-derived hydrazones:

(N.B. Diazo compound generation from ketone-derived hydrazones does not require a pre-conditioning phase for the MnO_2 column reactor)

A vial was charged with the appropriate alkyne (0.2 mmol, 1.0 equiv.), copper (I) iodide (3.9 mg, 0.02 mmol, 0.1 equiv.), 2,6-lutidine (4.6 µL, 0.04 mmol, 0.2 equiv.), 1,4-dioxane (2 mL), Et_3N (0.05 mL, 0.4 mmol, 2 equiv.) and pre-mixed for 10 min. A solution of hydrazone (0.1 M) and DIPEA (0.2 M) in CH_2Cl_2 was passed through the column reactor (Omnifit® column, 6.6 mm i.d. × 50 mm length), packed with activated MnO_2 (0.86 g), at a flow rate of 0.5 mL min^{-1}. When the FlowIR® showed that the intensity of the diazo peak was stable, 3 mL of the output (1.5 equiv. with respect to the hydrazone) was directly added into the reaction vial (over 6 min) containing the copper acetylide and the reaction mixture further stirred at r.t. for 10 min. The mixture was then filtered through a pad of Celite®, eluting with EtOAc, and the filtrate evaporated under reduced pressure. The residue was purified immediately by silica gel column chromatography to provide the desired tri-substituted allene product.

Any excess diazo compound produced during the generation phase before steady-state was reached was gently quenched by directing the output of the flow reactor into a stirred suspension of copper (I) iodide (0.10 g) in MeOH (25 mL) (**Figure 1**).

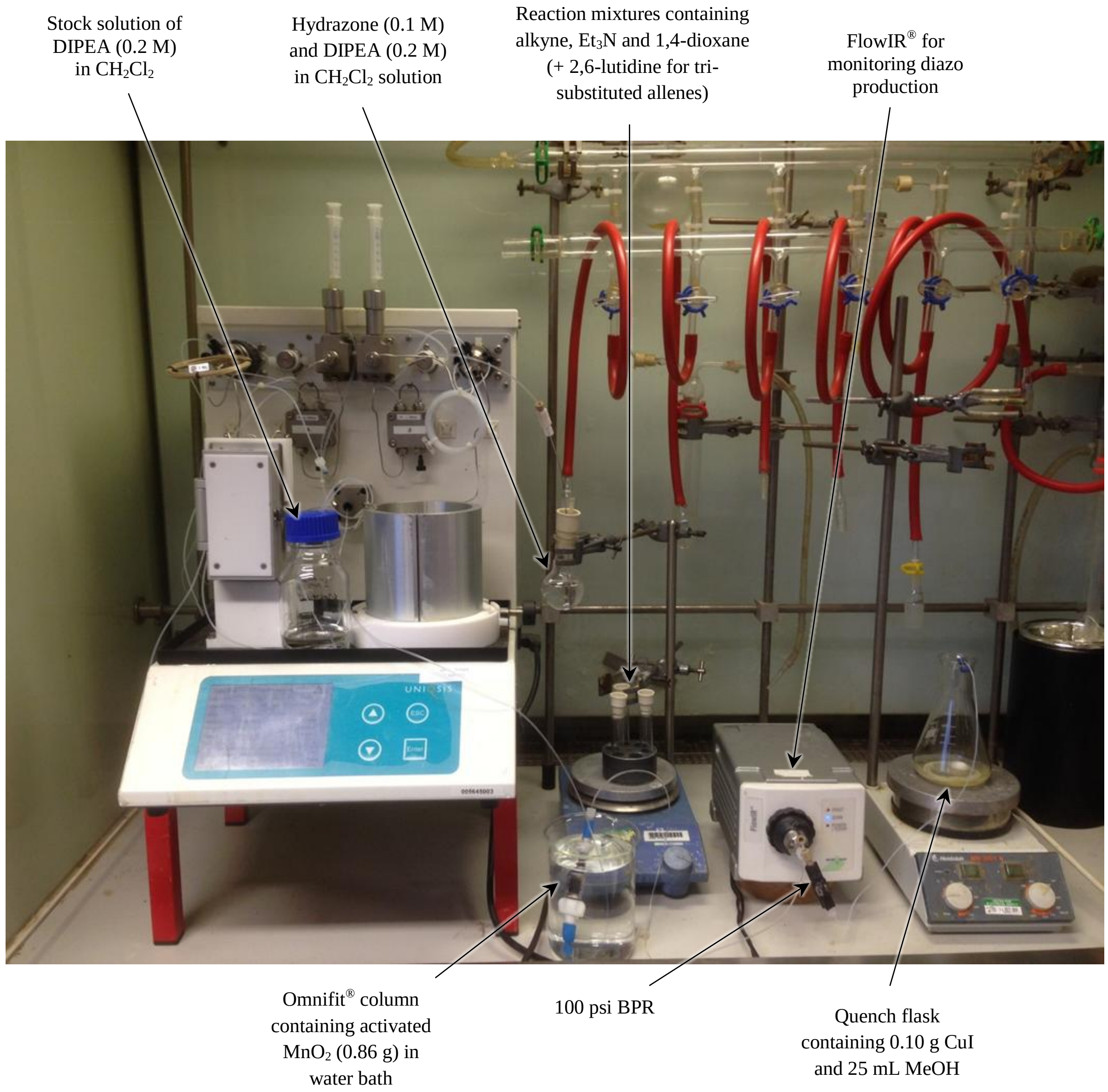

Figure 1: Reaction set-up for di- and tri-substituted allene synthesis on 0.2 mmol scale.

Scale-up procedure for allene 12f (Figure 2):

A 250 mL round-bottomed flask was charged with phenylacetylene (0.51 g, 5.0 mmol, 1.0 equiv.), copper (I) iodide (95 mg, 0.5 mmol, 0.1 equiv.), 2,6-lutidine (0.12 mL, 1.0 mmol, 0.2 equiv.), 1,4-dioxane (50 mL), Et_3N (1.39 mL, 10.0 mmol, 2.0 equiv.) and pre-mixed for 10 min. A Knauer pump[9] (flow rate: 1.0 mL min^{-1}) was used to continuously pump the solution through a FlowIR® device, monitoring at 2039 cm^{-1} for the diazo compound; the output was directed back into the reaction mixture. A solution of (1-(4-chlorophenyl)ethylidene)hydrazine (0.1 M) and DIPEA (0.2 M) in CH_2Cl_2 was passed through the column reactor (Omnifit® column, 0.79 cm i.d. × 15.0 cm length), packed with activated MnO_2 (12.0 g), at a flow rate of 4.5 mL min^{-1}. When the FlowIR® showed that the intensity of the diazo peak was stable, 75 mL of the output (1.5 equiv. with respect to the hydrazone) was directly added into the reaction flask (over 17 min) containing the copper acetylide and the reaction mixture further stirred at r.t. for 10 min. The mixture was then filtered through a pad of Celite®, eluting with EtOAc, and the filtrate evaporated under

reduced pressure. The residue was purified by silica gel column chromatography (eluent: hexane) to provide allene **12f** (0.88 g, 3.66 mmol, 73%) as a yellow oil.

Monitoring of the reaction mixture by FlowIR® indicated that negligible amounts of the diazo compound remained at the end of the reaction (**Figure 3**). A maximum absorption intensity of 0.01 A.U. at 2039 cm^{-1} was detected by the end of addition of the diazo compound, decreasing back to background levels after further stirring for 10 min (in comparison, a typical ~0.1 M solution of diazo compound generated using this flow oxidation procedure has an absorption intensity of ~0.2 A.U.).

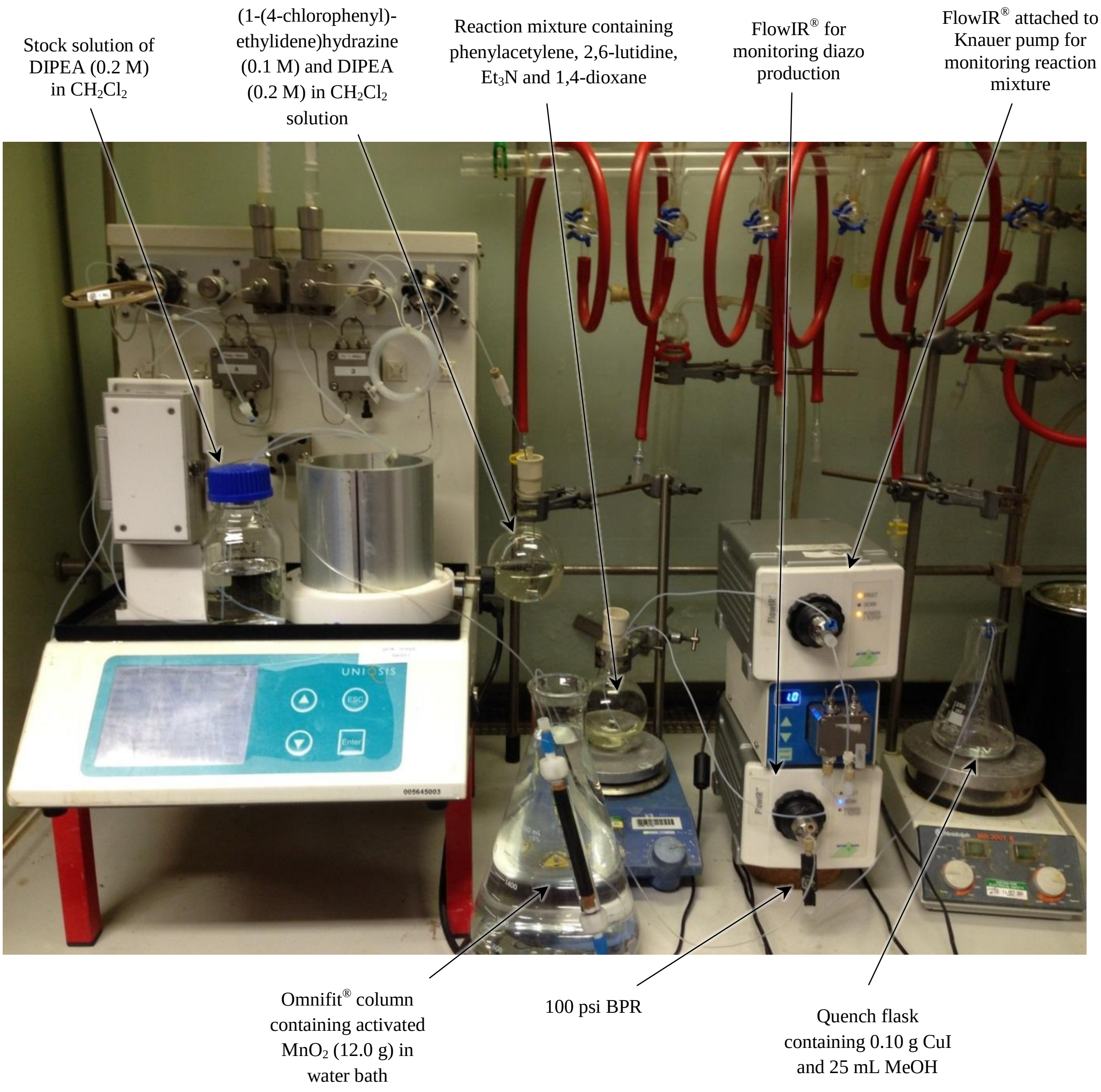

Figure 2: Reaction set-up for synthesis of allene **12f** on 5 mmol scale.

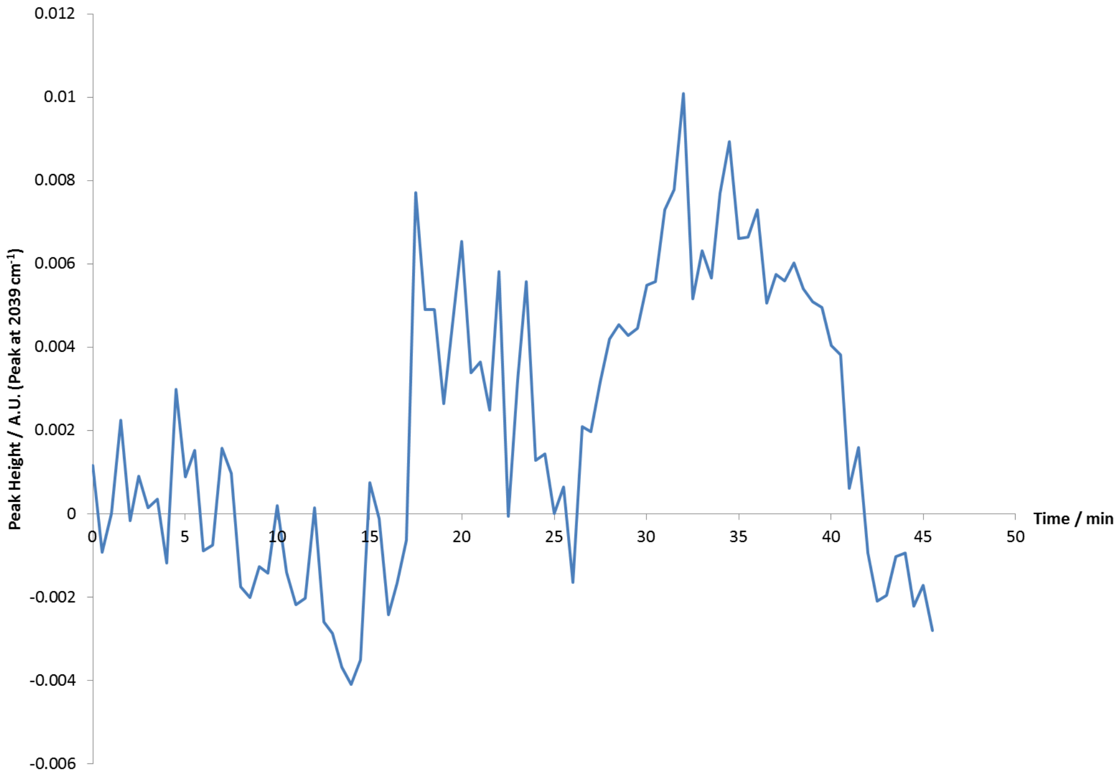

Figure 3: Monitoring of the diazo compound during the course of the reaction – addition of diazo compound was started at 15 min and terminated at 32 min.

4-(4-chlorophenyl)buta-2,3-dien-1-ol (4a):

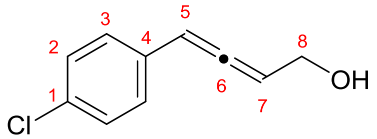

Isolated as a colourless oil (33.0 mg, 0.183 mmol, 91%) after silica gel column chromatography (eluent: 20% EtOAc/hexane), following the general procedure for allene formation with aldehyde-derived hydrazones.

^{1}H NMR (600 MHz, $CDCl_3$): δ 7.28 (d, J = 8.6 Hz, 2 H, H2), 7.23 (d, J = 8.6 Hz, 2 H, H3), 6.29 (dt, J = 6.0, 2.9 Hz, 1 H, H5), 5.80 (q, J = 6.0 Hz, 1 H, H7), 4.27 (br s, 2 H, H8), 1.80 (br s, 1 H, OH).
^{13}C NMR (150 MHz, $CDCl_3$): δ 204.4 (C6), 132.9 (C1), 132.4 (C4), 128.9 (C2), 128.1 (C3), 96.4 (C5), 96.3 (C7), 60.3 (C8).
FTIR (ν_{max}, cm^{-1}): 3330 (br m, OH), 2872 (w), 1951 (m, C=C=C), 1490 (s), 1428 (m), 1390 (m), 1352 (m), 1260 (w), 1198 (w), 1089 (s), 1012 (s), 872 (s), 830 (s).
HRMS (ESI+): calculated for $C_{10}H_{10}OCl$ $[M+H]^+$ 181.0415, found 181.0411.
R$_f$ = 0.31 (20% EtOAc/hexane).

4-(4-bromophenyl)buta-2,3-dien-1-ol (4b):

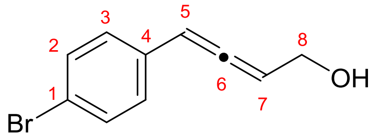

Isolated as a colourless oil (44.9 mg, 0.199 mmol, 99%) after silica gel column chromatography (eluent: 25% EtOAc/hexane), following the general procedure for allene formation with aldehyde-derived hydrazones.

^{1}H NMR (600 MHz, $CDCl_3$): δ 7.42 (d, J = 8.4 Hz, 2 H, H2), 7.16 (d, J = 8.4 Hz, 2 H, H3), 6.25 (dt, J = 6.0, 2.9 Hz, 1 H, H5), 5.77 (q, J = 6.0 Hz, 1 H, H7), 4.25 (br s, 2 H, H8), 1.84 (br s, 1 H, OH).
^{13}C NMR (150 MHz, $CDCl_3$): δ 204.5 (C6), 133.0 (C1), 131.9 (C2), 128.5 (C3), 121.0 (C4), 96.44 (C5/C7), 96.37 (C5/C7), 60.3 (C8).
FTIR (ν_{max}, cm^{-1}): 3344 (br m, OH), 2876 (w), 1950 (m, C=C=C), 1698 (m), 1588 (m), 1488 (s), 1428 (m), 1388 (m), 1264 (m), 1204 (m), 1175 (m), 1103 (m), 1069 (s), 1041 (m), 1008 (s), 873 (m), 828 (s).
HRMS (ESI+): calculated for $C_{10}H_{10}OBr$ $[M+H]^+$ 224.9910, found 224.9901.
R$_f$ = 0.27 (25% EtOAc/hexane).

4-(3-methoxyphenyl)buta-2,3-dien-1-ol (4c):

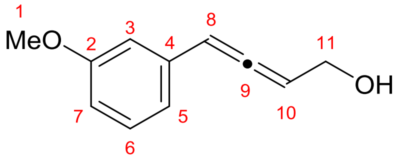

Isolated as a colourless oil (32.6 mg, 0.185 mmol, 93%) after silica gel column chromatography (eluent: 25% EtOAc/hexane), following the general procedure for allene formation with aldehyde-derived hydrazones. Data is consistent with a reported example.[10]

^{1}**H NMR (600 MHz, CDCl$_3$):** δ 7.23 (t, *J* = 7.9 Hz, 1 H, H6), 6.90 (d, *J* = 7.9 Hz, 1 H, H7), 6.85 (m, 1 H, H3), 6.77 (dd, *J* = 8.9, 2.4 Hz, 1 H, H5), 6.29 (dt, *J* = 6.0, 2.9 Hz, 1 H, H8), 5.78 (q, *J* = 6.0 Hz, 1 H, H10), 4.25 (br s, 2 H, H11), 3.80 (s, 3 H, H1), 1.94 (br s, 1 H, OH).
^{13}**C NMR (150 MHz, CDCl$_3$):** δ 204.4 (C9), 159.9 (C2), 135.3 (C4), 129.7 (C6), 119.5 (C7), 112.9 (C5), 112.2 (C3), 97.2 (C8), 96.0 (C10), 60.4 (C11), 55.3 (C1).
FTIR (ν_{max}, cm^{-1}): 3360 (br m, OH), 2940 (m), 2836 (w), 1950 (m, C=C=C), 1598 (s), 1584 (s), 1490 (s), 1466 (s), 1438 (m), 1408 (m), 1317 (m), 1265 (s), 1154 (s), 1111 (w), 1041 (s), 1011 (m), 875 (m).
HRMS (ESI+): calculated for $C_{11}H_{12}O_2Na$ $[M+Na]^+$ 199.0730, found 199.0720.
R$_f$ = 0.20 (25% EtOAc/hexane).

***N*-(4-(4-chlorophenyl)buta-2,3-dien-1-yl)-4-methylbenzenesulfonamide (4d):**

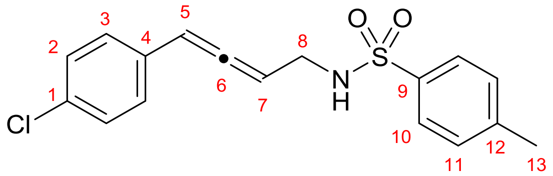

Isolated as an off-white amorphous solid (62.3 mg, 0.187 mmol, 93%) after silica gel column chromatography (eluent: 20% EtOAc/hexane), following the general procedure for allene formation with aldehyde-derived hydrazones.

^{1}**H NMR (400 MHz, CDCl$_3$):** δ 7.75 (d, *J* = 8.2 Hz, 2 H, H10), 7.25 (d, *J* = 8.2 Hz, 2 H, H11), 7.21 (d, *J* = 8.5 Hz, 2 H, H2), 7.11 (d, *J* = 8.5 Hz, 2 H, H3), 6.15 (dt, *J* = 6.2, 3.2 Hz, 1 H, H5), 5.52 (q, *J* = 6.2 Hz, 1 H, H7), 5.10 (t, *J* = 6.0 Hz, 1 H, NH), 3.68 (m, 2 H, H8), 2.40 (s, 3 H, H13).
^{13}**C NMR (100 MHz, CDCl$_3$):** δ 204.9 (C6), 143.7 (C9), 137.0 (C12), 133.1 (C1), 132.0 (C4), 129.8 (C11), 128.9 (C2), 128.2 (C3), 127.2 (C10), 96.9 (C5), 92.5 (C7), 41.7 (C8), 21.6 (C13).
FTIR (ν_{max}, cm^{-1}): 3276 (w, NH), 2924 (w), 1957 (w, C=C=C), 1723 (w), 1597 (w), 1491 (m), 1408 (w), 1323 (m), 1265 (w), 1155 (s), 1090 (s), 1013 (m), 874 (m), 834 (m), 812 (s).
HRMS (ESI+): calculated for $C_{17}H_{16}NO_2SClNa$ $[M+Na]^+$ 356.0482, found 356.0477.
R$_f$ = 0.24 (20% EtOAc/hexane).

***N*-(4-(4-bromophenyl)buta-2,3-dien-1-yl)-4-methylbenzenesulfonamide (4e):**

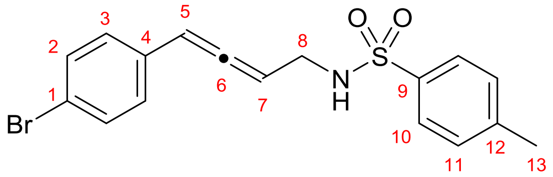

Isolated as an off-white amorphous solid (69.9 mg, 0.185 mmol, 92%) after silica gel column chromatography (eluent: 25% EtOAc/hexane) and trituration with Et_2O, following the general procedure for allene formation with aldehyde-derived hydrazones.

^{1}**H NMR (600 MHz, CDCl$_3$):** δ 7.74 (d, *J* = 8.2 Hz, 2 H, H10), 7.39 (d, *J* = 8.4 Hz, 2 H, H2), 7.27 (d, *J* = 8.2 Hz, 2 H, H11), 7.05 (d, *J* = 8.4 Hz, 2 H, H3), 6.15 (dt, *J* = 6.0, 3.1 Hz, 1 H, H5), 5.53 (q, *J* = 6.0 Hz, 1 H, H7), 4.69 (t, *J* = 5.7 Hz, 1 H, NH), 3.73 – 3.67 (m, 2 H, H8), 2.42 (s, 3 H, H13).
^{13}**C NMR (150 MHz, CDCl$_3$):** δ 204.9 (C6), 143.8 (C9), 137.0 (C12), 132.4 (C1), 131.9 (C2), 129.9 (C11), 128.6 (C3), 127.3 (C10), 121.4 (C4), 97.2 (C5), 92.7 (C7), 41.6 (C8), 21.7 (C13).

FTIR (ν_{max}, cm^{-1}): 3229 (m, NH), 1948 (w, C=C=C), 1597 (w), 1486 (m), 1454 (m), 1423 (m), 1346 (m), 1318 (m), 1305 (m), 1289 (m), 1235 (w), 1155 (s), 1124 (m), 1089 (m), 1067 (m), 1058 (m), 1008 (m), 916 (m), 877 (m), 828 (m), 817 (s), 809 (m).
HRMS (ESI+): calculated for $C_{17}H_{17}NO_2SBr$ $[M+H]^+$ 378.0158, found 378.0150.
R_f = 0.31 (25% EtOAc/hexane).

***N*-(4-(3-methoxyphenyl)buta-2,3-dien-1-yl)-4-methylbenzenesulfonamide (4f):**

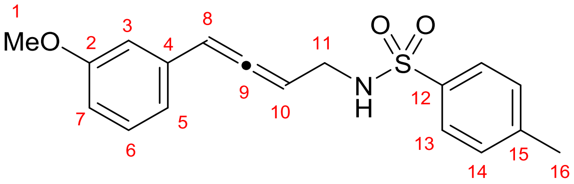

Isolated as a colourless oil (53.5 mg, 0.162 mmol, 81%) after silica gel column chromatography (eluent: 25% EtOAc/hexane), following the general procedure for allene formation with aldehyde-derived hydrazones.

^{1}H NMR (600 MHz, $CDCl_3$): δ 7.76 (d, *J* = 8.2 Hz, 2 H, H13), 7.28 (d, *J* = 8.2 Hz, 2 H, H14), 7.21 (t, *J* = 7.8 Hz, 1 H, H6), 6.81 (d, *J* = 7.8 Hz, 1 H, H7), 6.79 – 6.76 (m, 2 H, H3 and H5), 6.18 (dt, *J* = 6.0, 3.0 Hz, 1 H, H8), 5.53 (q, *J* = 6.0 Hz, 1 H, H10), 4.96 (t, *J* = 6.0 Hz, 1 H, NH), 3.80 (s, 3 H, H1), 3.69 (td, *J* = 6.0, 3.0 Hz, 2 H, H11), 2.42 (s, 3 H, H16).
^{13}C NMR (150 MHz, $CDCl_3$): δ 204.8 (C9), 159.9 (C2), 143.6 (C12), 136.8 (C15), 134.7 (C4), 129.8 (C14), 129.7 (C6), 127.2 (C13), 119.7 (C7), 113.2 (C5), 112.2 (C3), 97.8 (C8), 92.1 (C10), 55.3 (C1), 41.7 (C11), 21.6 (C16).
FTIR (ν_{max}, cm^{-1}): 3275 (w, NH), 1954 (w, C=C=C), 1598 (m), 1490 (m), 1454 (m), 1438 (m), 1321 (m), 1291 (m), 1265 (m), 1153 (s), 1092 (m), 1040 (m), 876 (m), 836 (m), 813 (m).
HRMS (ESI+): calculated for $C_{18}H_{19}NO_3SNa$ $[M+Na]^+$ 352.0978, found 352.0961.
R_f = 0.24 (25% EtOAc/hexane).

4-(4-hydroxybuta-1,2-dien-1-yl)benzonitrile (4g):

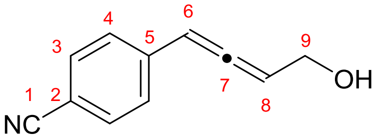

Isolated as an amorphous white solid (28.4 mg, 0.166 mmol, 83%) after silica gel column chromatography (eluent: 35% EtOAc/hexane), following the general procedure for allene formation with aldehyde-derived hydrazones.

^{1}H NMR (400 MHz, $CDCl_3$): δ 7.58 (d, *J* = 8.3 Hz, 2 H, H3), 7.38 (d, *J* = 8.3 Hz, 2 H, H4), 6.33 (dt, *J* = 6.1, 2.9 Hz, 1 H, H6), 5.87 (q, *J* = 6.0 Hz, 1 H, H8), 4.33 – 4.27 (m, 2 H, H9), 1.65 (t, *J* = 5.9 Hz, 1 H, OH).
^{13}C NMR (100 MHz, $CDCl_3$): δ 205.8 (C7), 139.3 (C5), 132.6 (C3), 127.4 (C4), 119.1 (C1), 110.6 (C2), 96.9 (C8), 96.5 (C6), 60.1 (C9).
FTIR (ν_{max}, cm^{-1}): 3406 (br m, OH), 2875 (w), 2227 (s, C≡N), 1948 (m, C=C=C), 1605 (s), 1505 (m), 1415 (m), 1202 (w), 1175 (m), 1108 (m), 1016 (s), 876 (m), 844 (s).
HRMS (ESI+): calculated for $C_{11}H_{10}NO$ $[M+H]^+$ 172.0757, found 172.0753.
R_f = 0.22 (35% EtOAc/hexane).

(*E*)-6-phenylhexa-2,3,5-trien-1-ol (4h):

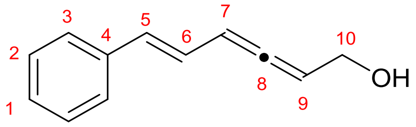

Isolated as a yellow oil (16.4 mg, 0.095 mmol, 48%) after silica gel column chromatography (eluent: 25% EtOAc/hexane), following the general procedure for allene formation with aldehyde-derived hydrazones.

^{1}H NMR (600 MHz, $CDCl_3$): δ 7.38 (d, *J* = 7.6 Hz, 2 H, H3), 7.31 (t, *J* = 7.6 Hz, 2 H, H2), 7.23 (t, *J* = 7.6 Hz, 1 H, H1), 6.61 (dd, *J* = 15.7, 10.3 Hz, 1 H, H6), 6.53 (d, *J* = 15.7 Hz, 1 H, H5), 6.17 (ddd, *J* = 10.3, 5.7, 2.8 Hz, 1 H, H7), 5.63 (q, *J* = 5.7 Hz, 1 H, H9), 4.21 (dt, *J* = 5.7, 2.8 Hz, 2 H, H10), 1.71 (br s, 1 H, OH).
^{13}C NMR (150 MHz, $CDCl_3$): δ 207.2 (C8), 137.1 (C4), 131.4 (C5), 128.7 (C2), 127.7 (C1), 126.4 (C3), 124.1 (C6), 97.4 (C7), 93.7 (C9), 60.6 (C10).
FTIR (ν_{max}, cm^{-1}): 3340 (m, OH), 3027 (w), 2929 (w), 1941 (m, C=C=C), 1597 (w), 1494 (w), 1450 (m), 1071 (m), 1009 (s), 964 (s), 877 (w).
HRMS (ESI+): calculated for $C_{12}H_{13}O$ $[M+H]^+$ 173.0961, found 173.0958.
$\boldsymbol{R_f}$ = 0.27 (25% EtOAc/hexane).

1-chloro-4-(3-cyclopentylpropa-1,2-dien-1-yl)benzene (4i):

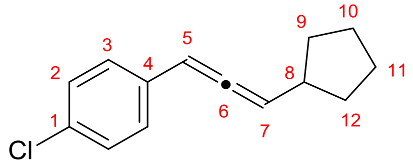

Isolated as a colourless oil (38.6 mg, 0.176 mmol, 88%) after silica gel column chromatography (eluent: hexane), following the general procedure for allene formation with aldehyde-derived hydrazones.

^{1}H NMR (600 MHz, $CDCl_3$): δ 7.25 (d, *J* = 8.6 Hz, 2 H, H2), 7.20 (d, *J* = 8.6 Hz, 2 H, H3), 6.10 (dd, *J* = 6.4, 2.8 Hz, 1 H, H5), 5.63 (t, *J* = 6.4 Hz, 1 H, H7), 2.64 – 2.55 (m, 1 H, H8), 1.88 – 1.81 (m, 2 H, H9a and H12a), 1.72 – 1.54 (m, 4 H, H10 and H11), 1.49 – 1.41 (m, 2 H, H9b and H12b).
^{13}C NMR (150 MHz, $CDCl_3$): δ 204.3 (C6), 133.9 (C1), 132.3 (C4), 128.8 (C2), 127.8 (C3), 100.6 (C7), 94.6 (C5), 39.3 (C8), 33.0 (C9/C12), 32.9 (C9/C12), 25.05 (C10/C11), 25.04 (C10/C11).
FTIR (ν_{max}, cm^{-1}): 2953 (m), 2868 (w), 1950 (w, C=C=C), 1491 (s), 1453 (w), 1387 (w), 1092 (m), 1013 (w), 877 (w), 833 (m).
HRMS (ESI+): calculated for $C_{14}H_{16}Cl$ $[M+H]^+$ 219.0935, found 219.0928.
$\boldsymbol{R_f}$ = 0.68 (hexane).

1-chloro-4-(3-cyclopropylpropa-1,2-dien-1-yl)benzene (4j):

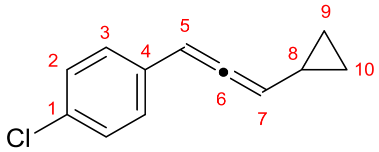

Isolated as a colourless oil (38.2 mg, 0.200 mmol, 99%) after silica gel column chromatography (eluent: hexane), following the general procedure for allene formation with aldehyde-derived hydrazones.

^{1}H NMR (600 MHz, CDCl$_3$): δ 7.26 (d, *J* = 8.5 Hz, 2 H, H2), 7.21 (d, *J* = 8.5 Hz, 2 H, H3), 6.17 (d, *J* = 6.4 Hz, 1 H, H5), 5.45 (t, *J* = 6.4 Hz, 1 H, H7), 1.38 – 1.33 (m, 1 H, H8), 0.80 – 0.74 (m, 2 H, H9a and H10a), 0.50 – 0.40 (m, 2 H, H9b and H10b).
^{13}C NMR (150 MHz, CDCl$_3$): δ 205.1 (C6), 133.6 (C1), 132.5 (C4), 128.8 (C2), 128.0 (C3), 100.1 (C7), 95.5 (C5), 9.5 (C8), 7.2 (C9/C10), 7.1 (C9/C10).
FTIR (ν_{max}, cm^{-1}): 3083 (w), 3005 (w), 1950 (w, C=C=C), 1490 (s), 1398 (w), 1252 (w), 1091 (s), 1048 (w), 1013 (m), 929 (w), 875 (m), 832 (s).
HRMS (ESI+): calculated for $C_{12}H_{12}Cl$ [M+H]$^+$ 191.0622, found 191.0614.
R$_f$ = 0.60 (hexane).

1-(5-bromopenta-1,2-dien-1-yl)-4-chlorobenzene (4k):

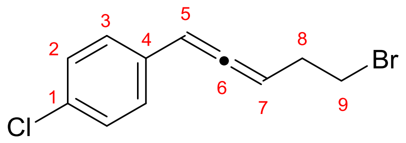

Isolated as a colourless oil (42.2 mg, 0.164 mmol, 82%) after silica gel column chromatography (eluent: hexane), following the general procedure for allene formation with aldehyde-derived hydrazones.

^{1}H NMR (600 MHz, CDCl$_3$): δ 7.27 (d, *J* = 8.6 Hz, 2 H, H2), 7.24 (d, *J* = 8.6 Hz, 2 H, H3), 6.18 (dt, *J* = 6.6, 2.8 Hz, 1 H, H5), 5.62 (q, *J* = 6.6 Hz, 1 H, H7), 3.48 (td, *J* = 6.6, 1.2 Hz, 2 H, H9), 2.75 – 2.64 (m, 2 H, H8).
^{13}C NMR (150 MHz, CDCl$_3$): δ 206.0 (C6), 132.81 (C1/C4), 132.76 (C1/C4), 128.9 (C2), 128.2 (C3), 95.1 (C5), 93.0 (C7), 32.2 (C8), 31.8 (C9).
FTIR (ν_{max}, cm^{-1}): 2967 (w), 1950 (w, C=C=C), 1727 (m), 1589 (m), 1491 (s), 1402 (m), 1267 (m), 1208 (m), 1090 (s), 1014 (m), 873 (m), 832 (s).
HRMS (ESI+): calculated for $C_{11}H_{11}BrCl$ [M+H]$^+$ 256.9727, found 256.9720.
R$_f$ = 0.35 (hexane).

1-bromo-4-(5-bromopenta-1,2-dien-1-yl)benzene (4l):

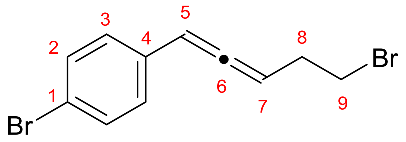

Isolated as a colourless oil (56.2 mg, 0.186 mmol, 93%) after silica gel column chromatography (eluent: hexane), following the general procedure for allene formation with aldehyde-derived hydrazones.

^{1}H NMR (600 MHz, CDCl$_3$): δ 7.42 (d, *J* = 8.5 Hz, 2 H, H2), 7.18 (d, *J* = 8.5 Hz, 2 H, H3), 6.16 (dt, *J* = 6.6, 2.8 Hz, 1 H, H5), 5.61 (q, *J* = 6.6 Hz, 1 H, H7), 3.48 (td, *J* = 6.6, 1.1 Hz, 2 H, H9), 2.75 – 2.64 (m, 2 H, H8).
^{13}C NMR (150 MHz, CDCl$_3$): δ 205.9 (C6), 133.2 (C1), 131.8 (C2), 128.5 (C3), 120.8 (C4), 95.2 (C5), 93.1 (C7), 32.1 (C8), 31.8 (C9).
FTIR (ν_{max}, cm^{-1}): 2963 (w), 1949 (w, C=C=C), 1700 (w), 1588 (w), 1487 (s), 1429 (w), 1386 (w), 1265 (m), 1207 (m), 1102 (w), 1069 (s), 1009 (s), 964 (w), 928 (w), 871 (s), 828 (s).
HRMS (ESI+): calculated for $C_{11}H_{11}Br_2$ [M+H]$^+$ 300.9222, found 300.9209.
R$_f$ = 0.27 (hexane).

1-chloro-4-(3-phenylpropa-1,2-dien-1-yl)benzene (4m):

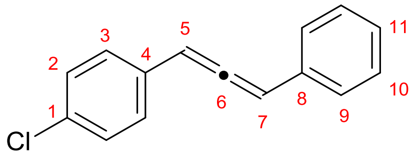

Isolated as a yellow oil (40.4 mg, 0.183 mmol, 92%) after silica gel column chromatography (eluent: hexane), following the general procedure for allene formation with aldehyde-derived hydrazones. Data is consistent with a reported example.[11]

^{1}H NMR (600 MHz, CDCl$_3$): δ 7.37 – 7.23 (m, 9 H, ArH), 6.61 (d, *J* = 6.6 Hz, 1 H, H5/H7), 6.56 (d, *J* = 6.6 Hz, 1 H, H5/H7).
^{13}C NMR (150 MHz, CDCl$_3$): δ 208.0 (C6), 133.4 (C1/C4/C8), 133.1 (C1/C4/C8), 132.3 (C1/C4/C8), 129.0 (CH), 128.9 (CH), 128.3 (CH), 127.7 (C11), 127.2 (CH), 99.0 (C5/C7), 97.7 (C5/C7).
FTIR (ν_{max}, cm^{-1}): 3030 (w), 1937 (w, C=C=C), 1597 (w), 1489 (s), 1458 (w), 1421 (w), 1386 (w), 1254 (w), 1193 (w), 1091 (s), 1013 (s), 964 (w), 913 (m), 878 (s), 832 (s).
HRMS (ESI+): calculated for $C_{15}H_{12}Cl$ $[M+H]^+$ 227.0622, found 227.0614.
R$_f$ = 0.45 (hexane)

1-chloro-4-(7-iodohepta-1,2-dien-1-yl)benzene (4n):

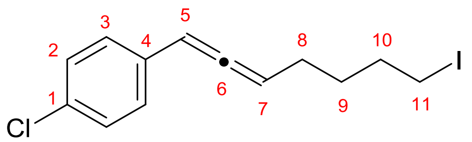

Isolated as a colourless oil (50.1 mg, 0.151 mmol, 75%) after silica gel column chromatography (eluent: hexane), following the general procedure for allene formation with aldehyde-derived hydrazones.

^{1}H NMR (600 MHz, CDCl$_3$): δ 7.27 (d, *J* = 8.5 Hz, 2 H, H2), 7.20 (d, *J* = 8.5 Hz, 2 H, H3), 6.11 (dt, *J* = 6.6, 3.0 Hz, 1 H, H5), 5.57 (q, *J* = 6.6 Hz, 1 H, H7), 3.19 (t, *J* = 7.0 Hz, 2 H, H11), 2.20 – 2.12 (m, 2 H, H8), 1.94 – 1.86 (m, 2 H, H10), 1.65 – 1.54 (m, 2 H, H9).
^{13}C NMR (150 MHz, CDCl$_3$): δ 205.4 (C6), 133.5 (C1), 132.4 (C4), 128.9 (C2), 127.9 (C3), 94.9 (C7), 94.3 (C5), 33.0 (C10), 29.9 (C9), 27.7 (C8), 6.6 (C11).
FTIR (ν_{max}, cm^{-1}): 2934 (w), 1949 (w, C=C=C), 1723 (m), 1703 (m), 1588 (m), 1490 (s), 1455 (m), 1427 (m), 1402 (m), 1265 (m), 1207 (m), 1169 (m), 1090 (s), 1013 (s), 878 (m), 820 (s).
HRMS (ESI+): calculated for $C_{13}H_{15}ClI$ $[M+H]^+$ 332.9901, found 332.9894.
R$_f$ = 0.33 (hexane).

1-chloro-4-(3-(cyclohex-1-en-1-yl)propa-1,2-dien-1-yl)benzene (4o):

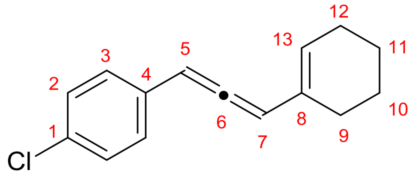

Isolated as a colourless oil (29.0 mg, 0.126 mmol, 63%) after silica gel column chromatography (eluent: hexane), following the general procedure for allene formation with aldehyde-derived hydrazones.

^{1}H NMR (600 MHz, CDCl$_3$): δ 7.26 (d, *J* = 8.6 Hz, 2 H, H2), 7.22 (d, *J* = 8.6 Hz, 2 H, H3), 6.35 (br d, *J* = 6.4 Hz, 1 H, H7), 6.26 (d, *J* = 6.4 Hz, 1 H, H5), 5.78 (br s, 1 H, H13), 2.17 – 2.12 (m, 2 H, H12), 2.12 – 2.06 (m, 1 H, H9a), 2.02 – 1.94 (m, 1 H, H9b), 1.68 – 1.58 (m, 4 H, H10 and H11).
^{13}C NMR (150 MHz, CDCl$_3$): δ 206.7 (C6), 133.5 (C1), 132.6 (C4), 131.7 (C8), 128.9 (C2), 128.0 (C3), 127.7 (C13), 102.1 (C5), 96.8 (C7), 26.1 (C9/C12), 25.9 (C9/C12), 22.6 (C10/C11), 22.5 (C10/C11).
FTIR (ν_{max}, cm^{-1}): 2928 (m), 2858 (w), 2833 (w), 1932 (w, C=C=C), 1703 (w), 1592 (w), 1490 (s), 1448 (w), 1434 (w), 1386 (w), 1348 (w), 1250 (w), 1136 (w), 1092 (s), 1013 (s), 917 (w), 884 (m), 846 (s), 831 (s).
HRMS (ESI+): calculated for $C_{15}H_{16}Cl$ [M+H]$^+$ 231.0935, found 231.0928.
R$_f$ = 0.53 (hexane).

7-(4-chlorophenyl)hepta-5,6-dienenitrile (4p):

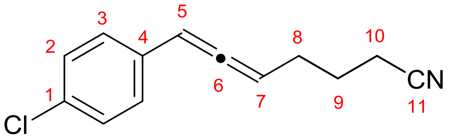

Isolated as a colourless oil (40.7 mg, 0.187 mmol, 93%) after silica gel column chromatography (eluent: 15% EtOAc/hexane), following the general procedure for allene formation with aldehyde-derived hydrazones.

^{1}H NMR (600 MHz, CDCl$_3$): δ 7.27 (d, *J* = 8.6 Hz, 2 H, H2), 7.19 (d, *J* = 8.6 Hz, 2 H, H3), 6.16 (dt, *J* = 6.4, 3.1 Hz, 1 H, H5), 5.59 (q, *J* = 6.4 Hz, 1 H, H7), 2.40 (t, *J* = 7.1 Hz, 2 H, H10), 2.33 – 2.26 (m, 2 H, H8), 1.90 – 1.79 (m, 2 H, H9).
^{13}C NMR (150 MHz, CDCl$_3$): δ 205.5 (C6), 133.0 (C1), 132.7 (C4), 129.0 (C2), 127.9 (C3), 119.4 (C11), 95.1 (C5), 93.6 (C7), 27.4 (C8), 24.6 (C9), 16.7 (C10).
FTIR (ν_{max}, cm^{-1}): 2937 (w), 2247 (w, C≡N), 1950 (w, C=C=C), 1732 (w), 1591 (w), 1499 (s), 1424 (w), 1390 (w), 1260 (w), 1174 (w), 1089 (s), 1013 (m), 880 (m), 832 (s).
HRMS (ESI+): calculated for $C_{13}H_{13}NCl$ [M+H]$^+$ 218.0731, found 218.0722.
R$_f$ = 0.25 (15% EtOAc/hexane).

***tert*-butyl (4-(4-chlorophenyl)buta-2,3-dien-1-yl)carbamate (4q):**

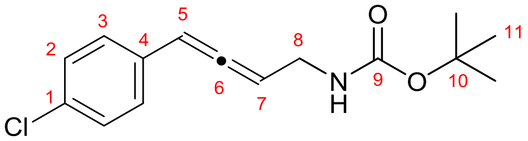

Isolated as a colourless oil (51.8 mg, 0.185 mmol, 93%) after silica gel column chromatography (eluent: 15% EtOAc/hexane), following the general procedure for allene formation with aldehyde-derived hydrazones.

^{1}H NMR (600 MHz, CDCl$_3$): δ 7.25 (d, *J* = 8.5 Hz, 2 H, H2), 7.20 (d, *J* = 8.5 Hz, 2 H, H3), 6.23 (dt, *J* = 6.4, 3.2 Hz, 1 H, H5), 5.64 (q, *J* = 6.4 Hz, 1 H, H7), 4.74 (br s, 1 H, NH), 3.92 – 3.72 (m, 2 H, H8), 1.39 (s, 9 H, H11).
^{13}C NMR (150 MHz, CDCl$_3$): δ 204.7 (C6), 155.8 (C9), 132.9 (C4), 132.6 (C1), 128.9 (C2), 128.2 (C3), 96.6 (C5), 94.1 (C7), 79.7 (C10), 39.1 (C8), 28.5 (C11).
FTIR (ν_{max}, cm^{-1}): 3344 (w, NH), 2978 (w), 2932 (w), 1953 (w, C=C=C), 1690 (s, C=O), 1490 (s), 1455 (w), 1430 (w), 1391 (m), 1366 (m), 1273 (m), 1248 (s), 1162 (s), 1091 (m), 1051 (w), 1013 (m), 952 (w), 912 (w), 860 (w), 831 (m).
HRMS (ESI+): calculated for $C_{15}H_{18}NO_2ClNa$ [M+Na]$^+$ 302.0918, found 302.0907.

R_f = 0.33 (15% EtOAc/hexane).

5-(4-chlorophenyl)penta-3,4-dien-1-ol (4r):

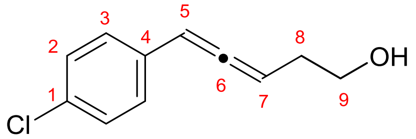

Isolated as a colourless oil (35.2 mg, 0.181 mmol, 90%) after silica gel column chromatography (eluent: 25% EtOAc/hexane), following the general procedure for allene formation with aldehyde-derived hydrazones.

^{1}H NMR (600 MHz, $CDCl_3$): δ 7.26 (d, J = 8.6 Hz, 2 H, H2), 7.21 (d, J = 8.6 Hz, 2 H, H3), 6.14 (dt, J = 6.6, 2.9 Hz, 1 H, H5), 5.61 (q, J = 6.6 Hz, 1 H, H7), 3.77 (t, J = 6.2 Hz, 2 H, H9), 2.43 – 2.35 (m, 2 H, H8), 1.69 (br s, 1 H, OH).
^{13}C NMR (150 MHz, $CDCl_3$): δ 205.9 (C6), 133.1 (C1), 132.6 (C4), 128.9 (C2), 127.9 (C3), 94.3 (C5), 92.0 (C7), 62.0 (C9), 32.1 (C8).
FTIR (ν_{max}, cm^{-1}): 3319 (br w, OH), 2284 (w), 1952 (w, C=C=C), 1491 (s), 1390 (w), 1090 (m), 1048 (m), 1013 (m), 877 (m), 834 (m).
HRMS (ESI+): calculated for $C_{11}H_{12}OCl$ $[M+H]^+$ 195.0571, found 195.0564.
R_f = 0.22 (25% EtOAc/hexane).

5-(4-chlorophenyl)penta-3,4-dien-2-ol (4s):

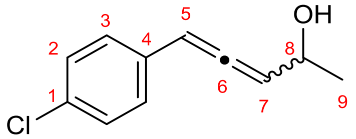

Isolated as an inseparable mixture of diastereomers (1:1) as a colourless oil (30.2 mg, 0.165 mmol, 83%) after silica gel column chromatography (eluent: 20% EtOAc/hexane), following the general procedure for allene formation with aldehyde-derived hydrazones.

^{1}H NMR (600 MHz, $CDCl_3$): δ 7.27 (two superimposed d, J = 8.5 Hz, 2 H, H2), 7.21 (two d, J = 8.5 Hz, 2 H, H3), 6.28 – 6.25 (m, 1 H, H5), 5.74 (two t, J = 6.0 Hz, 1 H, H7), 4.52 – 4.44 (m, 1 H, H8), 1.83 (two superimposed br s, 1 H, OH), 1.38 (two d, J = 6.4 Hz, 3 H, H9).
^{13}C NMR (150 MHz, $CDCl_3$): δ 203.40 and 203.37 (C6), 132.9 (C1), 132.63 and 132.56 (C4), 129.0 (C2), 128.04 and 128.03 (C3), 101.38 and 101.37 (C7), 96.8 and 96.6 (C5), 66.26 and 66.00 (C8), 23.79 and 23.71 (C9).
FTIR (ν_{max}, cm^{-1}): 3358 (br w, OH), 2978 (w), 1951 (w, C=C=C), 1701 (m), 1590 (m), 1490 (m), 1402 (m), 1376 (m), 1265 (m), 1207 (m), 1172 (m), 1090 (s), 1013 (s), 924 (m), 878 (m), 821 (s).
HRMS (ESI+): calculated for $C_{11}H_{12}OCl$ $[M+H]^+$ 195.0571, found 195.0565.
R_f = 0.25 (20% EtOAc/hexane).

4-(4-chlorophenyl)-1-phenylbuta-2,3-dien-1-ol (4t):

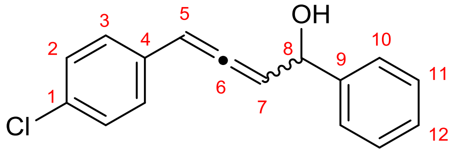

Isolated as a separable diastereomers (1:1) as off-white amorphous solids (A: 23.7 mg, 0.092 mmol; B: 26.3 mg, 0.102 mmol; combined yield 97%) after silica gel column

chromatography (eluent: 15% EtOAc/hexane), following the general procedure for allene formation with aldehyde-derived hydrazones.

Diastereomer A:
^{1}H NMR (600 MHz, $CDCl_3$): δ 7.44 (d, *J* = 7.3 Hz, 2 H, H10), 7.39 (t, *J* = 7.3 Hz, 2 H, H11), 7.32 (t, *J* = 7.3 Hz, 1 H, H12), 7.28 (d, *J* = 8.5 Hz, 2 H, H2), 7.24 (d, *J* = 8.5 Hz, 2 H, H3), 6.33 (dd, *J* = 6.2, 2.5 Hz, 1 H, H5), 5.90 (t, *J* = 6.2 Hz, 1 H, H7), 5.39 – 5.35 (m, 1 H, H8), 2.19 (br d, *J* = 3.7 Hz, 1 H, OH).
^{13}C NMR (150 MHz, $CDCl_3$): δ 203.8 (C6), 142.8 (C9), 133.1 (C4), 132.4 (C1), 129.0 (C2), 128.8 (C11), 128.21 (C3), 128.17 (C12), 126.2 (C10), 100.6 (C7), 97.4 (C5), 72.3 (C8).
FTIR (ν_{max}, cm^{-1}): 3295 (br m, OH), 3062 (w), 1950 (w, C=C=C), 1499 (s), 1450 (m), 1430 (w), 1192 (w), 1090 (m), 1013 (s), 879 (m), 841 (m).
HRMS (ESI+): calculated for $C_{16}H_{14}OCl$ $[M+H]^+$ 257.0728, found 257.0717.
R_f = 0.25 (15% EtOAc/hexane).

Diastereomer B:
^{1}H NMR (600 MHz, $CDCl_3$): δ 7.44 (d, *J* = 7.3 Hz, 2 H, H10), 7.38 (t, *J* = 7.3 Hz, 2 H, H11), 7.32 (t, *J* = 7.3 Hz, 1 H, H12), 7.26 (d, *J* = 8.5 Hz, 2 H, H2), 7.18 (d, *J* = 8.5 Hz, 2 H, H3), 6.33 (dd, *J* = 6.3, 2.2 Hz, 1 H, H4), 5.89 (t, *J* = 6.3 Hz, 1 H, H7), 5.42 – 5.38 (m, 1 H, H8), 2.20 (br d, *J* = 6.6 Hz, 1 H, OH).
^{13}C NMR (150 MHz, $CDCl_3$): δ 204.1 (C6), 142.9 (C9), 133.1 (C4), 132.4 (C1), 129.0 (C2), 128.8 (C11), 128.17 (C3), 128.16 (C12), 126.1 (C10), 100.5 (C7), 97.1 (C5), 72.5 (C8).
FTIR (ν_{max}, cm^{-1}): 3390 (br w, OH), 3066 (w), 3033 (w), 1949 (w, C=C=C), 1490 (s), 1455 (m), 1426 (w), 1386 (w), 1254 (m), 1197 (w), 1109 (w), 1090 (s), 1045 (m), 1028 (w), 1012 (m), 922 (w), 886 (m), 840 (s), 828 (m), 814 (w).
HRMS (ESI+): calculated for $C_{16}H_{14}OCl$ $[M+H]^+$ 257.0728, found 257.0717.
R_f = 0.21 (15% EtOAc/hexane).

5-(4-chlorophenyl)-2-methylpenta-3,4-dien-2-ol (4u):

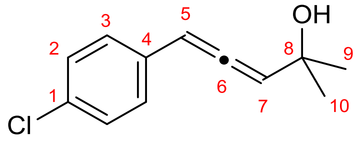

Isolated as a colourless oil (34.0 mg, 0.163 mmol, 82%) after silica gel column chromatography (eluent: 20% EtOAc/hexane), following the general procedure for allene formation with aldehyde-derived hydrazones.

^{1}H NMR (600 MHz, $CDCl_3$): δ 7.27 (d, *J* = 8.5 Hz, 2 H, H2), 7.21 (d, *J* = 8.5 Hz, 2 H, H3), 6.28 (d, *J* = 6.4 Hz, 1 H, H5), 5.80 (d, *J* = 6.4 Hz, 1 H, H7), 1.83 (br s, 1 H, OH), 1.429 (s, 3 H, H9/H10), 1.426 (s, 3 H, H9/H10).
^{13}C NMR (150 MHz, $CDCl_3$): δ 202.2 (C6), 132.9 (C4), 132.7 (C1), 129.0 (C2), 127.9 (C3), 105.6 (C7), 97.1 (C5), 70.4 (C8), 30.3 (C9/C10), 30.2 (C9/C10).
FTIR (ν_{max}, cm^{-1}): 3368 (br w, OH), 2976 (m), 2931 (w), 1951 (w, C=C=C), 1700 (m), 1592 (w), 1491 (s), 1426 (w), 1375 (m), 1149 (s), 1092 (s), 1014 (s), 967 (m), 878 (m), 834 (s).
HRMS (ESI+): calculated for $C_{12}H_{14}OCl$ $[M+H]^+$ 209.0728, found 209.0720.
R_f = 0.26 (20% EtOAc/hexane).

2-(((4-(4-chlorophenyl)buta-2,3-dien-1-yl)oxy)methyl)-3-phenyloxirane (4v):

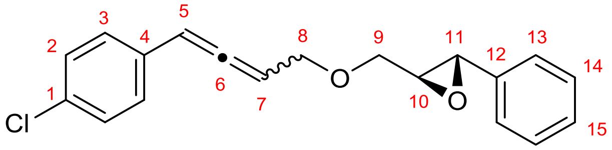

Isolated as an inseparable mixture of diastereomers (1:1) as a colourless oil (55.4 mg, 0.177 mmol, 89%) after silica gel column chromatography (eluent: 10% EtOAc/hexane), following the general procedure for allene formation with aldehyde-derived hydrazones.

^{1}H NMR (600 MHz, $CDCl_3$): δ 7.37 – 7.33 (m, 2 H, H14), 7.33 – 7.29 (m, 1 H, H15), 7.29 – 7.25 (m, 4 H, H2 and H13), 7.23 (two superimposed d, J = 8.3 Hz, 2 H, H3), 6.26 – 6.22 (m, 1 H, H5), 5.72 (two superimposed q, J = 6.5 Hz, 1 H, H7), 4.26 – 4.22 (m, 2 H, H8), 3.89 (two superimposed ddd, J = 11.5, 8.9, 3.0 Hz, 1 H, H9a), 3.80 (two superimposed dd, J = 7.5, 3.0 Hz, 1 H, H11), 3.68 – 3.66 (m, 1 H, H9b), 3.25 – 3.22 (m, 1 H, H10).
^{13}C NMR (150 MHz, $CDCl_3$): δ 206.16 and 206.13 (C6), 136.87 and 136.86 (C12), 132.89 and 132.88 (C4), 132.45 and 132.44 (C1), 128.94 and 128.93 (C2), 128.6 (C14), 128.4 (C15), 128.16 and 128.15 (C3), 125.81 and 125.80 (C13), 95.0 (C5), 92.9 (C7), 69.92 and 69.91 (C9), 69.03 and 69.01 (C8), 61.1 (C10), 56.04 and 56.01 (C11).
FTIR (ν_{max}, cm^{-1}): 2990 (w), 2857 (w), 1951 (w, C=C=C), 1490 (m), 1462 (w), 1431 (w), 1391 (w), 1352 (w), 1308 (w), 1242 (w), 1201 (w), 1090 (s), 1013 (m), 969 (w), 873 (s), 832 (s).
HRMS (ESI+): calculated for $C_{19}H_{17}O_2ClNa$ $[M+Na]^+$ 335.0809, found 335.0804.
R_f = 0.30 (10% EtOAc/hexane).

3-(4-chloro)phenylpropa-1,2-dienylferrocene (4w):

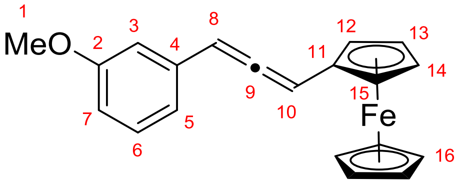

Isolated as a red oil (61.0 mg, 0.185 mmol, 92%) after silica gel column chromatography (eluent: 4% EtOAc/hexane), following the general procedure for allene formation with aldehyde-derived hydrazones.

^{1}H NMR (600 MHz, $CDCl_3$): δ 7.27 (t, J = 8.1 Hz, 1 H, H6), 6.98 (d, J = 8.1 Hz, 1 H, H5), 6.94 (s, 1 H, H3), 6.81 (d, J = 8.1 Hz, 1 H, H7), 6.34 (d, J = 6.4 Hz, 1 H, H10), 6.31 (d, J = 6.4 Hz, 1 H, H8), 4.36 (s, 1 H, H12/H15), 4.32 (s, 1 H, H12/H15), 4.23 (s, 2 H, H13 and H14), 4.21 (s, 5 H, H16), 3.83 (s, 3 H, H1).
^{13}C NMR (150 MHz, $CDCl_3$): δ 206.2 (C9), 160.0 (C2), 135.8 (C4), 129.7 (C6), 119.6 (C5), 112.9 (C7), 112.1 (C3), 97.2 (C8), 95.2 (C10), 79.7 (C11), 69.4 (C16), 68.8 (C13/C14), 68.7 (C13/C14), 67.7 (C12/C15), 67.1 (C12/C15), 55.3 (C1).
FTIR (ν_{max}, cm^{-1}): 3086 (w), 2939 (w), 2834 (w), 1936 (w, C=C=C), 1595 (m), 1581 (m), 1488 (m), 1464 (m), 1452 (m), 1435 (m), 1410 (w), 1316 (w), 1291 (m), 1264 (s), 1224 (m), 1152 (m), 1105 (m), 1044 (s), 1000 (m), 924 (w), 873 (m), 817 (s).
HRMS (ESI+): calculated for $C_{20}H_{18}OFe$ $[M]^+$ 330.0702, found 330.0687.
R_f = 0.41 (4% EtOAc/hexane).

1-chloro-4-(4,4-diethoxybuta-1,2-dien-1-yl)benzene (4x):

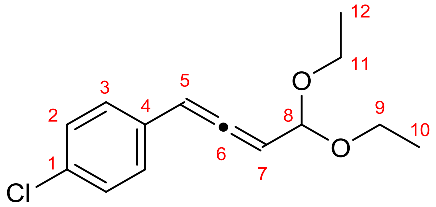

Isolated as an off-white amorphous solid (47.8 mg, 0.199 mmol, 99%) after silica gel column chromatography (eluent: 4% EtOAc/hexane), following the general procedure for allene formation with aldehyde-derived hydrazones.

^{1}H NMR (600 MHz, $CDCl_3$): δ 7.27 (d, J = 8.6 Hz, 2 H, H2), 7.22 (d, J = 8.6 Hz, 2 H, H3), 6.28 (dd, J = 6.1, 1.5 Hz, 1 H, H5), 5.66 (t, J = 6.1 Hz, 1 H, H7), 5.06 (dd, J = 6.1, 1.5 Hz, 1 H, H8), 3.71 (dq overlaps with 3.68 peak, J = 9.5, 7.1 Hz, 1 H, H9a/H11a), 3.68 (dq overlaps with 3.71 peak, J = 9.5, 7.1 Hz, 1 H, H9a/H11a), 3.58 (two superimposed tq, J = 9.5, 7.1 Hz, 2 H, H9b and H11b), 1.24 (t, J = 7.1 Hz, 3 H, H10/H12), 1.23 (t, J = 7.1 Hz, 3 H, H10/H12).
^{13}C NMR (150 MHz, $CDCl_3$): δ 205.8 (C6), 133.0 (C4), 132.3 (C1), 128.9 (C2), 128.2 (C3), 100.2 (C8), 96.0 (C5), 95.4 (C7), 61.7 (C9/C11), 61.5 (C9/C11), 15.31 (C10/C12), 15.28 (C10/C12).
FTIR (ν_{max}, cm^{-1}): 2976 (w), 2881 (w), 1956 (w, C=C=C), 1491 (m), 1432 (w), 1388 (w), 1330 (w), 1090 (s), 1053 (s), 1013 (m), 872 (w), 833 (m).
HRMS (ESI+): calculated for $C_{14}H_{17}OClNa$ $[M+Na]^+$ 275.0809, found 275.0800.
R$_f$ = 0.31 (4% EtOAc/hexane).

(8*R*,9*S*,10*R*,13*S*,14*S*,17*R*)-17-(3-(4-chlorophenyl)propa-1,2-dien-1-yl)-17-hydroxy-13-methyl-1,2,6,7,8,9,10,11,12,13,14,15,16,17-tetradecahydro-3*H*-cyclopenta[*a*]phenanthren-3-one (9):

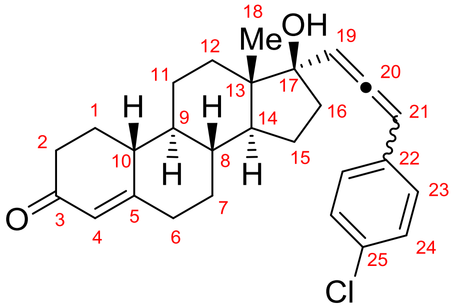

Isolated as separable diastereomers (1.5:1) as off-white amorphous solids (A: 33.6 mg, 0.079 mmol; B: 20.0 mg, 0.047 mmol; combined yield 63%) after silica gel column chromatography (diastereomer A – eluent: 40% EtOAc/hexane, then 80% Et_2O/hexane; diastereomer B – eluent: 40% EtOAc/hexane), following the general procedure for allene formation with aldehyde-derived hydrazones.

Diastereomer A:
^{1}H NMR (600 MHz, $CDCl_3$): δ 7.27 (d, J = 8.6 Hz, 2 H, H24), 7.23 (d, J = 8.6 Hz, 2 H, H23), 6.30 (d, J = 6.2 Hz, 1 H, H21), 5.84 (s, 1 H, H4), 5.80 (d, J = 6.2 Hz, 1 H, H19), 2.51 – 2.40 (m, 2 H, steroidal H/OH), 2.31 – 2.24 (m, 3 H, steroidal H/OH), 2.14 – 2.09 (m, 1 H, steroidal H/OH), 2.05 – 1.99 (m, 1 H, steroidal H/OH), 1.94 – 1.83 (m, 3 H, steroidal H/OH), 1.67 – 1.45 (m, 4 H, steroidal H/OH), 1.42 – 1.25 (m, 5 H, steroidal H/OH), 1.11 – 1.03 (m, 1 H, steroidal H/OH), 0.99 (s, 3 H, H18), 0.91 – 0.84 (m, 1 H, steroidal H/OH).
^{13}C NMR (150 MHz, $CDCl_3$): δ 202.6 (C20), 200.0 (C3), 166.7 (C5), 133.0 (C22), 132.8 (C25), 129.0 (C24), 128.0 (C23), 124.7 (C4), 102.8 (C21), 97.2 (C19), 83.6 (C17), 49.5 (CH),

48.9 (C14), 47.1 (C13), 42.7 (C10), 41.2 (CH), 36.72 (CH_2), 36.65 (CH_2), 35.6 (CH_2), 32.4 C12), 31.0 (CH_2), 26.8 (CH_2), 26.3 (CH_2), 23.5 (CH_2), 14.2 (C18).
FTIR (ν_{max}, cm^{-1}): 3409 (br w, OH), 2932 (m), 2867 (m), 1945 (w, C=C=C), 1660 (s, C=O), 1491 (m), 1452 (w), 1428 (w), 1362 (w), 1332 (w), 1261 (w), 1208 (w), 1130 (w), 1091 (w), 1063 (w), 1013 (m), 968 (w), 910 (w), 883 (w), 837 (w).
HRMS (ESI+): calculated for $C_{27}H_{32}OCl$ $[M+H]^+$ 423.2085, found 423.2069.
R_f = 0.29 (40% EtOAc/hexane); 0.36 (80% Et_2O/hexane).

Diastereomer B:
^{1}H NMR (600 MHz, $CDCl_3$): δ 7.25 (d, *J* = 8.5 Hz, 2 H, H24), 7.19 (d, *J* = 8.5 Hz, 2 H, H23), 6.30 (d, *J* = 6.3 Hz, 1 H, H21), 5.85 (s, 1 H, H4), 5.81 (d, *J* = 6.3 Hz, 1 H, H19), 2.48 – 2.40 (m, 2 H, steroidal H), 2.33 – 2.23 (m, 3 H, steroidal H), 2.16 – 2.09 (m, 2 H, steroidal H), 1.95 – 1.87 (m, 2 H, steroidal H), 1.84 (br s, 1 H, OH), 1.79 – 1.74 (m, 1 H, steroidal H), 1.69 – 1.64 (m, 1 H, steroidal H), 1.61 – 1.55 (m, 1 H, steroidal H), 1.53 – 1.47 (m, 2 H, steroidal H), 1.42 – 1.25 (m, 4 H, steroidal H), 0.99 (s, 3 H, H18), 0.89 – 0.80 (m, 2 H, steroidal H).
^{13}C NMR (150 MHz, $CDCl_3$): δ 203.1 (C20), 200.0 (C3), 166.5 (C5), 133.0 (C22), 132.8 (C25), 128.9 (C24), 128.0 (C23), 124.8 (C4), 102.2 (C21), 97.0 (C19), 83.4 (C17), 49.8 (CH), 48.7 (C14), 46.8 (C13), 42.7 (C10), 41.1 (CH), 36.7 (CH_2), 36.4 (CH_2), 35.5 (CH_2), 32.2 (C12), 30.9 (CH_2), 26.8 (CH_2), 26.2 (CH_2), 23.2 (CH_2), 14.1 (C18).
FTIR (ν_{max}, cm^{-1}): 3421 (br w, OH), 2932 (m), 2866 (m), 1946 (w, C=C=C), 1660 (s, C=O), 1491 (m), 1451 (w), 1427 (w), 1362 (w), 1332 (w), 1261 (w), 1208 (w), 1131 (w), 1091 (m), 1060 (w), 1013 (m), 967 (w), 883 (w), 836 (w).
HRMS (ESI+): calculated for $C_{27}H_{32}OCl$ $[M+H]^+$ 423.2085, found 423.2069.
R_f = 0.23 (40% EtOAc/hexane).

4-(4-((1*R*)-(6-methoxyquinolin-4-yl)((2*S*)-5-vinylquinuclidin-2-yl)methoxy)buta-1,2-dien-1-yl)benzonitrile (11):

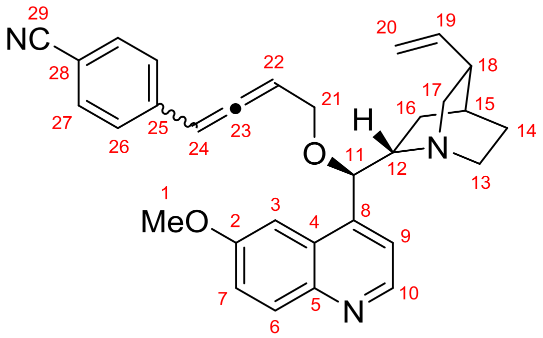

Isolated as an inseparable mixture of diastereomers (1:1) as a brown film (78.3 mg, 0.164 mmol, 82%) after silica gel column chromatography (eluent: 30% EtOAc/60% hexane/10% Et_3N), following the general procedure for allene formation with aldehyde-derived hydrazones.

^{1}H NMR (600 MHz, $CDCl_3$): δ 8.75 – 8.68 (m, 1 H, H10), 8.01 (m, 1 H, H6), 7.57 – 7.20 (m, 7 H, ArH), 6.21 (m, 1 H, H24), 5.82 – 5.72 (m, 1 H, H22), 5.66 (m, 1 H, H19), 5.23 (br s, 1 H, H11), 4.95 – 4.80 (m, 2 H, H20), 4.16 – 3.95 (m, 2 H, H21), 3.93 – 3.84 (m, 3 H, H1), 3.37 – 3.21 (m, 1 H, H13a), 3.12 – 2.98 (m, 2 H, H12 and H17a), 2.71 – 2.53 (m, 2 H, H13b and H17b), 2.28 – 2.16 (m, 1 H, H18), 1.80 – 1.32 (m, 5 H, H14, H15 and H16).
^{13}C NMR (150 MHz, $CDCl_3$): δ 207.2 and 206.8 (C23), 157.9 (C2), 147.6 (C10), 144.6 (C8), 144.3 (C5), 141.9 (C19), 139.08 and 139.05 (C25), 132.44 and 132.38 (C27), 131.9 (C6), 127.32 and 127.22 (C26), 127.19 (C4), 121.7 and 121.6 (C7), 119.5 – 118.5 (br, C9), 119.0 (C29), 114.4 (C20), 110.34 and 110.29 (C28), 101.2 (br, C3), 95.5 and 95.2 (C24), 93.8 and

93.5 (C22), 77.5 – 77.0 (br, obscured by $CDCl_3$ peak, C11), 66.4 and 66.3 (C21), 60.0 (C12), 57.3 (C17), 55.79 and 55.71 (C1), 43.4 (C13), 40.01 and 39.95 (C18), 27.87 and 27.83 (C15), 27.78 and 27.6 (C14), 24.0 – 20.0 (br, C16).
FTIR (ν_{max}, cm^{-1}): 2935 (m), 2865 (w), 2225 (m, C≡N), 1950 (w, C=C=C), 1620 (s), 1605 (m), 1507 (s), 1472 (m), 1454 (m), 1432 (m), 1355 (w), 1301 (w), 1259 (m), 1240 (s), 1227 (s), 1174 (w), 1133 (m), 1076 (s), 1030 (s), 992 (w), 910 (s), 875 (m), 843 (s), 831 (s).
HRMS (ESI+): calculated for $C_{31}H_{32}N_3O_2$ $[M+H]^+$ 478.2489, found 478.2481.
R_f = 0.28 (30% EtOAc/60% hexane/10% Et_3N).

4-methyl-*N*-(4-phenylpenta-2,3-dien-1-yl)benzenesulfonamide (12a):

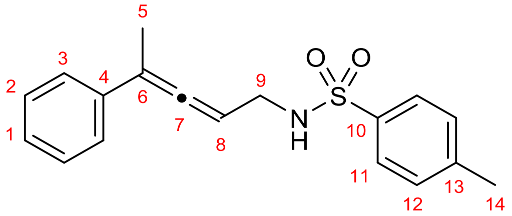

Isolated as a colourless oil (41.7 mg, 0.133 mmol, 67%) after silica gel column chromatography (eluent: 20% EtOAc/hexane), following the general procedure for allene formation with ketone-derived hydrazones.

^{1}H NMR (600 MHz, $CDCl_3$): δ 7.74 (d, *J* = 8.2 Hz, 2 H, H11), 7.31 (m, 4 H, H2 and H3), 7.27 (d, *J* = 8.2 Hz, 2 H, H12), 7.25 – 7.21 (m, 1 H, H1), 5.42 (tq, *J* = 5.9, 2.9 Hz, 1 H, H8), 4.66 (t, *J* = 5.9 Hz, 1 H, NH), 3.67 (t, *J* = 5.9 Hz, 2 H, H9), 2.41 (s, 3 H, H14), 2.05 (d, *J* = 2.9 Hz, 3 H, H5).
^{13}C NMR (150 MHz, $CDCl_3$): δ 203.6 (C7), 143.6 (C4), 137.0 (C10), 136.2 (C13), 129.9 (C12), 128.5 (C2/C3), 127.31 (C1), 127.26 (C11), 125.9 (C2/C3), 104.4 (C6), 90.0 (C8), 42.1 (C9), 21.7 (C14), 17.1 (C5).
FTIR (ν_{max}, cm^{-1}): 3292 (br w, NH), 2924 (w), 1950 (w, C=C=C), 1723 (w), 1681 (w), 1598 (w), 1494 (w), 1447 (w), 1327 (m), 1267 (w), 1155 (s), 1091 (m), 909 (m), 813 (m).
HRMS (ESI+): calculated for $C_{18}H_{19}NO_2SNa$ $[M+Na]^+$ 336.1029, found 336.1022.
R_f = 0.26 (20% EtOAc/hexane).

***tert*-butyl (4-phenylpenta-2,3-dien-1-yl)carbamate (12b):**

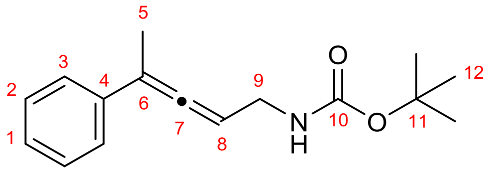

Isolated as a colourless oil (38.2 mg, 0.147 mmol, 74%) after silica gel column chromatography (eluent: 10% EtOAc/hexane), following the general procedure for allene formation with ketone-derived hydrazones.

^{1}H NMR (600 MHz, $CDCl_3$): δ 7.40 (d, *J* = 7.6 Hz, 2 H, H3), 7.32 (t, *J* = 7.6 Hz, 2 H, H2), 7.22 (t, *J* = 7.6 Hz, 1 H, H1), 5.56 – 5.48 (m, 1 H, H8), 4.69 (s, 1 H, NH), 3.82 (br s, 2 H, H9), 2.12 (d, *J* = 2.9 Hz, 3 H, H5), 1.42 (s, 9 H, H12).
^{13}C NMR (150 MHz, $CDCl_3$): δ 203.5 (C7), 155.8 (C10), 136.8 (C4), 128.5 (C2), 127.0 (C1), 125.9 (C3), 103.6 (C6), 91.5 (C8), 79.5 (C11), 39.5 (C9), 28.5 (C12), 17.2 (C5).
FTIR (ν_{max}, cm^{-1}): 3354 (br w, NH), 2978 (w), 2930 (w), 1952 (w, C=C=C), 1690 (s, C=O), 1598 (w), 1495 (m), 1446 (w), 1391 (w), 1366 (m), 1247 (s), 1164 (s), 1067 (w), 1050 (w), 1026 (w), 941 (w), 909 (w), 860 (w).

HRMS (ESI+): calculated for $C_{16}H_{21}NO_2Na$ $[M+Na]^+$ 282.1465, found 282.1454.
$\boldsymbol{R_f}$ = 0.32 (10% EtOAc/hexane).

(5-methoxypenta-2,3-dien-2-yl)benzene (12c):

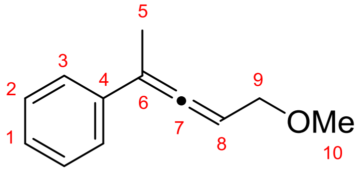

Isolated as a colourless oil (22.8 mg, 0.131 mmol, 65%) after silica gel column chromatography (eluent: 5% EtOAc/hexane), following the general procedure for allene formation with ketone-derived hydrazones.

^{1}H NMR (600 MHz, $CDCl_3$): δ 7.42 (d, *J* = 7.3 Hz, 2 H, H3), 7.33 (t, *J* = 7.3 Hz, 2 H, H2), 7.22 (t, *J* = 7.3 Hz, 1 H, H1), 5.55 (tq, *J* = 6.8, 2.9 Hz, 1 H, H8), 4.05 (d, *J* = 6.8 Hz, 2 H, H9), 3.39 (s, 3 H, H10), 2.14 (d, *J* = 2.9 Hz, 3 H, H5).
^{13}C NMR (150 MHz, $CDCl_3$): δ 205.2 (C7), 136.8 (C4), 128.5 (C2), 126.9 (C1), 125.9 (C3), 101.5 (C6), 90.5 (C8), 70.8 (C9), 57.9 (C10), 17.2 (C5).
FTIR (ν_{max}, cm^{-1}): 2982 (w), 2927 (w), 2817 (w), 1952 (w, C=C=C), 1741 (w), 1598 (w), 1494 (m), 1445 (m), 1351 (w), 1262 (w), 1185 (w), 1099 (s), 1067 (m), 1027 (w), 957 (w), 913 (m).
HRMS (ESI+): calculated for $C_{12}H_{15}O$ $[M+H]^+$ 175.1117, found 175.1112.
$\boldsymbol{R_f}$ = 0.33 (5% EtOAc/hexane).

7-phenylocta-5,6-dienenitrile (12d):

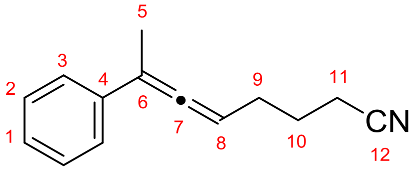

Isolated as a colourless oil (29.0 mg, 0.147 mmol, 73%) after silica gel column chromatography (eluent: 10% EtOAc/hexane), following the general procedure for allene formation with ketone-derived hydrazones.

^{1}H NMR (600 MHz, $CDCl_3$): δ 7.39 (d, *J* = 7.4 Hz, 2 H, H3), 7.33 (t, *J* = 7.4 Hz, 2 H, H2), 7.22 (t, *J* = 7.4 Hz, 1 H, H1), 5.46 (tq, *J* = 7.1, 2.9 Hz, 1 H, H8), 2.40 (t, *J* = 7.2 Hz, 2 H, H11), 2.27 (q, *J* = 7.1 Hz, 2 H, H9), 2.11 (d, *J* = 2.9 Hz, 3 H, H5), 1.90 – 1.78 (m, 2 H, H10).
^{13}C NMR (150 MHz, $CDCl_3$): δ 204.5 (C7), 137.1 (C4), 128.5 (C2), 126.9 (C1), 125.7 (C3), 119.7 (C12), 101.9 (C6), 91.1 (C8), 27.8 (C9), 24.7 (C10), 17.3 (C5), 16.6 (C11).
FTIR (ν_{max}, cm^{-1}): 2940 (w), 2247 (w, C≡N), 1951 (w, C=C=C), 1727 (w), 1685 (w), 1598 (w), 1493 (s), 1445 (s), 1371 (w), 1288 (w), 1264 (w), 1183 (w), 1067 (m), 1026 (m), 912 (w), 844 (w).
HRMS (ESI+): calculated for $C_{14}H_{16}N$ $[M+H]^+$ 198.1277, found 198.1270.
$\boldsymbol{R_f}$ = 0.33 (10% EtOAc/hexane).

tert-butyl (4-(4-chlorophenyl)penta-2,3-dien-1-yl)carbamate (12e):

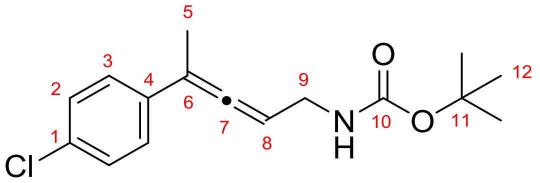

Isolated as a colourless oil (52.4 mg, 0.178 mmol, 89%) after silica gel column chromatography (eluent: 15% EtOAc/hexane), following the general procedure for allene formation with ketone-derived hydrazones.

^{1}H NMR (600 MHz, CDCl$_3$): δ 7.30 (d, *J* = 8.6 Hz, 2 H, H2), 7.26 (d, *J* = 8.6 Hz, 2 H, H3), 5.53 – 5.47 (m, 1 H, H8), 4.69 (br s, 1 H, NH), 3.85 – 3.73 (m, 2 H, H9), 2.07 (d, *J* = 2.9 Hz, 3 H, H5), 1.40 (s, 9 H, H12).
^{13}C NMR (150 MHz, CDCl$_3$): δ 203.5 (C7), 155.8 (C10), 135.4 (C4), 132.7 (C1), 128.5 (C3), 127.2 (C2), 102.8 (C6), 92.0 (C8), 79.6 (C11), 39.4 (C9), 28.5 (C12), 17.2 (C5).
FTIR (ν_{max}, cm^{-1}): 3348 (br w, NH), 2978 (w), 2931 (w), 1954 (w, C=C=C), 1690 (s, C=O), 1490 (s), 1455 (w), 1392 (m), 1366 (m), 1249 (m), 1165 (s), 1095 (m), 1063 (w), 1012 (m), 941 (w), 860 (w), 831 (w).
HRMS (ESI+): calculated for $C_{16}H_{20}NO_2ClNa$ $[M+Na]^+$ 316.1075, found 316.1067.
R_f = 0.41 (15% EtOAc/hexane).

1-chloro-4-(4-phenylbuta-2,3-dien-2-yl)benzene (12f):

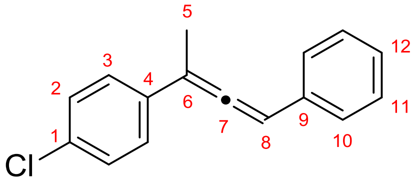

Isolated as a yellow oil (52.4 mg, 0.178 mmol, 89%) after silica gel column chromatography (eluent: hexane), following the general procedure for allene formation with ketone-derived hydrazones. On scale-up using 5 mmol of phenylacetylene: isolated as a yellow oil (0.88 g, 3.66 mmol, 73%). Data is consistent with a reported example.[12]

^{1}H NMR (600 MHz, CDCl$_3$): δ 7.38 (d, *J* = 8.6 Hz, 2 H, H2), 7.34 – 7.31 (m, 4 H, H10 and H11), 7.29 (d, *J* = 8.6 Hz, 2 H, H3), 7.26 – 7.21 (m, 1 H, H12), 6.49 (q, *J* = 2.9 Hz, 1 H, H8), 2.21 (d, *J* = 2.9 Hz, 3 H, H5).
^{13}C NMR (150 MHz, CDCl$_3$): δ 206.9 (C7), 135.0 (C4), 134.3 (C9), 132.9 (C1), 128.9 (C10/C11), 128.7 (C3), 127.3 (C12), 127.2 (C2), 127.1 (C10/C11), 103.9 (C6), 97.1 (C8), 16.90 (C5).
FTIR (ν_{max}, cm^{-1}): 3028 (w), 1934 (w, C=C=C), 1689 (w), 1597 (w), 1489 (s), 1449 (w), 1408 (w), 1257 (w), 1198 (w), 1093 (s), 1062 (w), 1012 (s), 913 (w), 833 (s), 818 (s).
HRMS (ESI+): calculated for $C_{16}H_{14}Cl$ $[M+H]^+$ 241.0779, found 241.0770.
R_f = 0.39 (hexane).

3-(3-(4-chlorophenyl)buta-1,2-dien-1-yl)thiophene (12g):

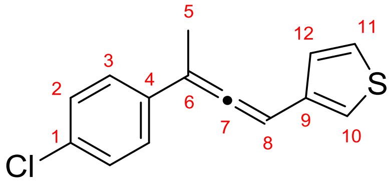

Isolated as a yellow oil (26.3 mg, 0.107 mmol, 53%) after silica gel column chromatography (eluent: hexane), following the general procedure for allene formation with ketone-derived hydrazones.

^{1}H NMR (600 MHz, $CDCl_3$): δ 7.36 (d, *J* = 8.6 Hz, 2 H, H2), 7.28 (d, *J* = 8.6 Hz, 2 H, H3), 7.27 (dd, *J* = 5.0, 2.9 Hz, 1 H, H11), 7.14 (dd, *J* = 2.9, 1.2 Hz, 1 H, H10), 7.05 (dd, *J* = 5.0, 1.2 Hz, 1 H, H12), 6.55 (q, *J* = 2.9 Hz, 1 H, H8), 2.18 (d, *J* = 2.9 Hz, 3 H, H5).
^{13}C NMR (150 MHz, $CDCl_3$): δ 207.0 (C7), 135.6 (C9), 135.2 (C4), 132.9 (C1), 128.7 (C3), 127.3 (C2), 126.5 (C12), 126.2 (C11), 121.3 (C10), 103.0 (C6), 91.6 (C8), 17.1 (C5).
FTIR (ν_{max}, cm^{-1}): 3103 (w), 2986 (w), 1940 (w, C=C=C), 1688 (w), 1591 (w), 1489 (s), 1440 (w), 1402 (w), 1370 (w), 1235 (w), 1180 (w), 1146 (w), 1092 (s), 1062 (w), 1012 (s), 858 (w), 834 (s), 820 (s).
HRMS (ESI+): calculated for $C_{14}H_{12}SCl$ $[M+H]^+$ 247.0343, found 247.0335.
R$_f$ = 0.52 (hexane).

(3-cyclopropylpropa-1,2-diene-1,1-diyl)dibenzene (12h):

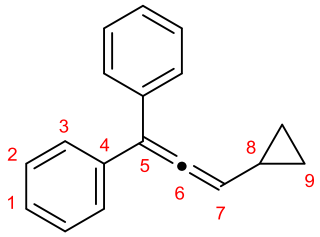

Isolated as a yellow oil (36.2 mg, 0.156 mmol, 78%) after silica gel column chromatography (eluent: hexane), following a modified version of the general procedure for allene formation with ketone-derived hydrazones, utilising THF instead of CH_2Cl_2 as solvent for the diazo generation and with pumping of the diazo compound over 8 min (2.0 equiv. with respect to hydrazone).

^{1}H NMR (600 MHz, $CDCl_3$): δ 7.38 – 7.32 (m, 8 H, H2 and H3), 7.29 – 7.26 (m, 2 H, H1), 5.56 (d, *J* = 7.6 Hz, 1 H, H7), 1.48 – 1.38 (m, 1 H, H8), 0.84 – 0.75 (m, 2 H, H9a), 0.53 – 0.45 (m, 2 H, H9b).
^{13}C NMR (150 MHz, $CDCl_3$): δ 205.4 (C6), 137.3 (C4), 128.54 (C2/C3), 128.46 (C2/C3), 127.2 (C1), 111.4 (C5), 99.0 (C7), 9.9 (C8), 7.2 (C9).
FTIR (ν_{max}, cm^{-1}): 3080 (w), 3057 (w), 3005 (w), 1943 (w, C=C=C), 1662 (w), 1597 (m), 1492 (s), 1443 (m), 1277 (w), 1177 (w), 1156 (w), 1073 (w), 1048 (w), 1020 (m), 965 (w), 937 (m), 921 (w), 901 (m), 850 (w), 807 (w).
HRMS (ESI+): calculated for $C_{18}H_{17}$ $[M+H]^+$ 233.1325, found 233.1317.
R$_f$ = 0.25 (hexane).

tert-butyl (4,4-diphenylbuta-2,3-dien-1-yl)carbamate (12i):

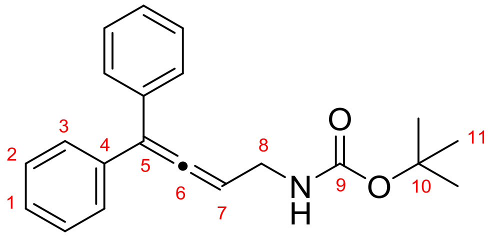

Isolated as a yellow oil (55.8 mg, 0.174 mmol, 87%) after silica gel column chromatography (eluent: 15% EtOAc/hexane), following a modified version of the general procedure for allene formation with ketone-derived hydrazones, utilising THF instead of CH_2Cl_2 as solvent for the diazo generation and with pumping of the diazo compound over 8 min (2.0 equiv. with respect to hydrazone).

^{1}H NMR (600 MHz, $CDCl_3$): δ 7.36 – 7.32 (m, 8 H, H2 and H3), 7.31 – 7.26 (m, 2 H, H1), 5.75 (t, J = 5.2 Hz, 1 H, H7), 4.73 (br s, 1 H, NH), 3.90 (br s, 2 H, H8), 1.41 (s, 9 H, H11).
^{13}C NMR (150 MHz, $CDCl_3$): δ 204.8 (C6), 155.8 (C9), 136.5 (C4), 128.6 (C2/C3), 128.5 (C2/C3), 127.6 (C1), 112.7 (C5), 93.0 (C7), 79.7 (C10), 39.4 (C8), 28.5 (C11).
FTIR (ν_{max}, cm^{-1}): 3348 (br w, NH), 2976 (w), 2931 (w), 1946 (w, C=C=C), 1693 (s, C=O), 1598 (w), 1492 (s), 1453 (m), 1391 (w), 1366 (m), 1340 (w), 1273 (w), 1247 (m), 1166 (s), 1074 (w), 1051 (w), 1031 (w), 921 (w), 862 (w).
HRMS (ESI+): calculated for $C_{21}H_{23}NO_2Na$ $[M+Na]^+$ 344.1621, found 344.1612.
R_f = 0.37 (15% EtOAc/hexane).

4,4-diphenylbuta-2,3-dien-1-ol (12j):

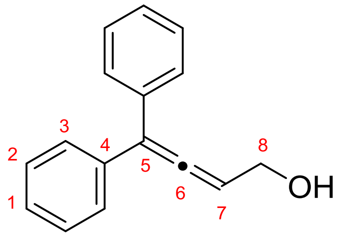

Isolated as a yellow oil (35.7 mg, 0.161 mmol, 80%) after silica gel column chromatography (eluent: 25% EtOAc/hexane), following a modified version of the general procedure for allene formation with ketone-derived hydrazones, utilising THF instead of CH_2Cl_2 as solvent for the diazo generation and with pumping of the diazo compound over 8 min (2.0 equiv. with respect to hydrazone). Data is consistent with a reported example.[13]

^{1}H NMR (600 MHz, $CDCl_3$): δ 7.38 – 7.33 (m, 8 H, H2 and H3), 7.32 – 7.28 (m, 2 H, H1), 5.91 (t, J = 5.8 Hz, 1 H, H7), 4.32 (d, J = 5.8 Hz, 2 H, H8), 1.64 (br s, 1 H, OH).
^{13}C NMR (150 MHz, $CDCl_3$): δ 204.5 (C6), 136.5 (C4), 128.64 (C2/C3), 128.58 (C2/C3), 127.7 (C1), 112.7 (C5), 95.3 (C7), 60.8 (C8).
FTIR (ν_{max}, cm^{-1}): 3326 (br m, OH), 3057 (w), 2869 (w), 1942 (w, C=C=C), 1597 (m), 1492 (s), 1452 (m), 1443 (m), 1075 (m), 1013 (s), 923 (w), 902 (w).
HRMS (ESI+): calculated for $C_{16}H_{15}O$ $[M+H]^+$ 223.1117, found 223.1109.
R_f = 0.30 (25% EtOAc/hexane).

***tert*-butyl (4-(4-(trifluoromethyl)phenyl)octa-2,3,7-trien-1-yl)carbamate (12k):**

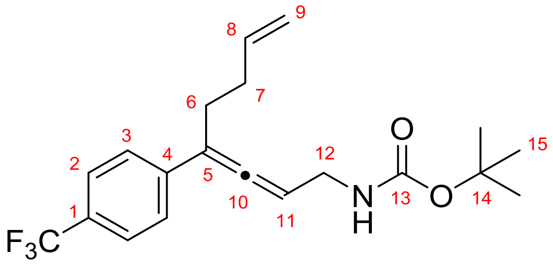

Isolated as a colourless oil (42.4 mg, 0.115 mmol, 58%) after silica gel column chromatography (eluent: 15% EtOAc/hexane), following the general procedure for allene formation with ketone-derived hydrazones.

^{1}H NMR (600 MHz, $CDCl_3$): δ 7.55 (d, *J* = 8.2 Hz, 2 H, H2), 7.49 (d, *J* = 8.2 Hz, 2 H, H3), 5.88 (ddt, *J* = 16.8, 10.2, 6.5 Hz, 1 H, H8), 5.67 – 5.62 (m, 1 H, H11), 5.09 – 5.01 (m, 2 H, H9a and H9b), 4.72 (br s, 1 H, NH), 3.88 (dt, *J* = 15.7, 5.5 Hz, 1 H, H12a/H12b), 3.79 (dt, *J* = 15.7, 5.5 Hz, 1 H, H12a/H12b), 2.61 – 2.48 (m, 2 H, H6), 2.33 – 2.28 (m, 2 H, H7), 1.40 (s, 9 H, H15).
^{13}C NMR (150 MHz, $CDCl_3$): δ 203.9 (C10), 155.8 (C13), 140.4 (C4), 137.9 (C8), 129.0 (q, *J* = 32.4 Hz, C1), 126.4 (C3), 125.4 (q, *J* = 3.8 Hz, C2), 124.37 (q, *J* = 271.7 Hz, C1), 115.5 (C9), 107.6 (C5), 94.2 (C11), 79.7 (C14), 39.2 (C12), 31.9 (C7), 29.2 (C6), 28.5 (C15).
^{19}F NMR (376 MHz, $CDCl_3$): -62.5 (s).
FTIR (ν_{max}, cm^{-1}): 3351 (br w, OH), 2979 (w), 2931 (w), 1949 (w, C=C=C), 1694 (m, C=O), 1616 (w), 1504 (m), 1422 (w), 1393 (w), 1367 (w), 1325 (s), 1277 (w), 1249 (m), 1164 (s), 1123 (s), 1069 (m), 1015 (w), 914 (w), 845 (w).
HRMS (ESI+): calculated for $C_{20}H_{24}F_3NO_2Na$ $[M+Na]^+$ 390.1651, found 390.1641.
R_f = 0.42 (15% EtOAc/hexane).

2-(8-bromo-4-(4-methoxyphenyl)octa-4,5-dien-1-yl)-5,5-dimethyl-1,3-dioxane (12l):

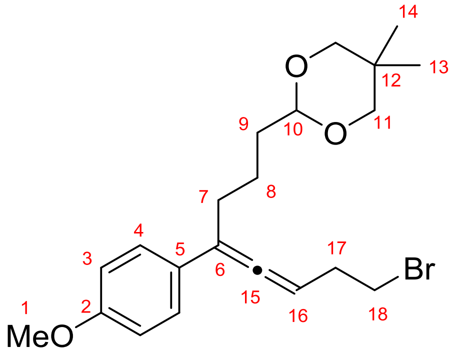

Isolated as a yellow oil (45.3 mg, 0.111 mmol, 55%) after silica gel column chromatography (eluent: 5% → 10% Et_2O/hexane), following the general procedure for allene formation with ketone-derived hydrazones.

^{1}H NMR (400 MHz, $CDCl_3$): δ 7.32 (d, *J* = 8.8 Hz, 2 H, H4), 6.85 (d, *J* = 8.8 Hz, 2 H, H3), 5.54 – 5.47 (m, 1 H, H16), 4.44 (t, *J* = 4.7 Hz, 1 H, H10), 3.80 (s, 3 H, H1), 3.60 (d, *J* = 10.9 Hz, 2 H, H11a/H11b), 3.46 (t, *J* = 7.1 Hz, 2 H, H18), 3.41 (d, *J* = 10.9 Hz, 2 H, H11a/H11b), 2.65 (q, *J* = 7.1 Hz, 2 H, H17), 2.46 – 2.39 (m, 2 H, H7), 1.77 – 1.62 (m, 4 H, H8 and H9), 1.19 (s, 3 H, H13/H14), 0.71 (s, 3 H, H13/H14).

^{13}C NMR (100 MHz, $CDCl_3$): δ 204.2 (C15), 158.7 (C2), 128.9 (C5), 127.3 (C4), 114.0 (C3), 106.4 (C6), 102.2 (C10), 92.0 (C16), 77.4 (C11), 55.4 (C1), 34.7 (C7), 32.9 (C17), 32.3 (C18), 30.3 (C9), 30.0 (C12), 23.2 (C13/C14), 22.6 (C8), 22.0 (C13/C14).
FTIR (ν_{max}, cm^{-1}): 2953 (m), 2836 (w), 1943 (w, C=C=C), 1607 (m), 1578 (w), 1510 (s), 1464 (m), 1394 (m), 1363 (w), 1292 (m), 1248 (s), 1177 (m), 1131 (s), 1097 (m), 1037 (m), 970 (w), 924 (w), 886 (w), 833 (w).
HRMS (ESI+): calculated for $C_{21}H_{30}O_3Br$ $[M+H]^+$ 409.1373, found 409.1362.
R_f = 0.31 (15% Et_2O/hexane).

4. Deuteration experiment

1-chloro-4-(3-cyclopropylpropa-1,2-dien-1-yl-3-*d*)benzene ([D]-4j):

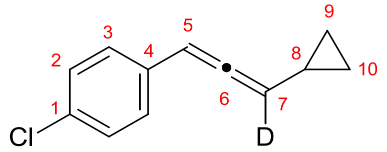

The same procedure for the synthesis of allene **4j** was utilised with a minor modification to the protocol: CD_3OD (25 µL, 0.6 mmol, 3.0 equiv.) was added to the mixture of alkyne, Et_3N and 1,4-dioxane prior to pre-mixing for 10 min. A 37:63 mixture of allenes [D]-**4j** and **4j** was obtained as a colourless oil (35.5 mg, 0.186 mmol, combined yield 93%) after silica gel column chromatography (eluent: hexane).

^{1}H NMR (600 MHz, $CDCl_3$): δ 7.26 (d, J = 8.6 Hz, 2 H, H2), 7.21 (d, J = 8.6 Hz, 2 H, H3), 6.20 – 6.13 (m, 1 H, H5), 5.44 (dd, J = 7.5, 6.6 Hz, 0.63 H, H7 of **4j**), 1.44 – 1.28 (m, 1 H, H8), 0.81 – 0.73 (m, 2 H, H9a and H10a), 0.50 – 0.40 (m, 2 H, H9b and H10b).
^{13}C NMR (150 MHz, $CDCl_3$): δ 205.0 (C6), 133.6 (C1), 132.4 (C4), 128.8 (C2), 127.9 (C3), 100.1 (C7), 95.5 (C5 of [D]-**4j**), 95.4 (C5 of **4j**), 9.6 (C8 of **4j**), 9.5 (C8 of [D]-**4j**), 7.21 (C9/C10 of **4j**), 7.19 (C9/C10 of [D]-**4j**), 7.12 (C9/C10 of **4j**), 7.10 (C9/C10 of [D]-**4j**).
HRMS (ESI+): calculated for $C_{12}H_{11}DCl$ $[M+H]^+$ 192.0685, found 192.0677.
R_f = 0.60 (hexane).

5. NMR spectra

4-(4-chlorophenyl)buta-2,3-dien-1-ol (4a):

^{1}H NMR, 600 MHz, $CDCl_3$:

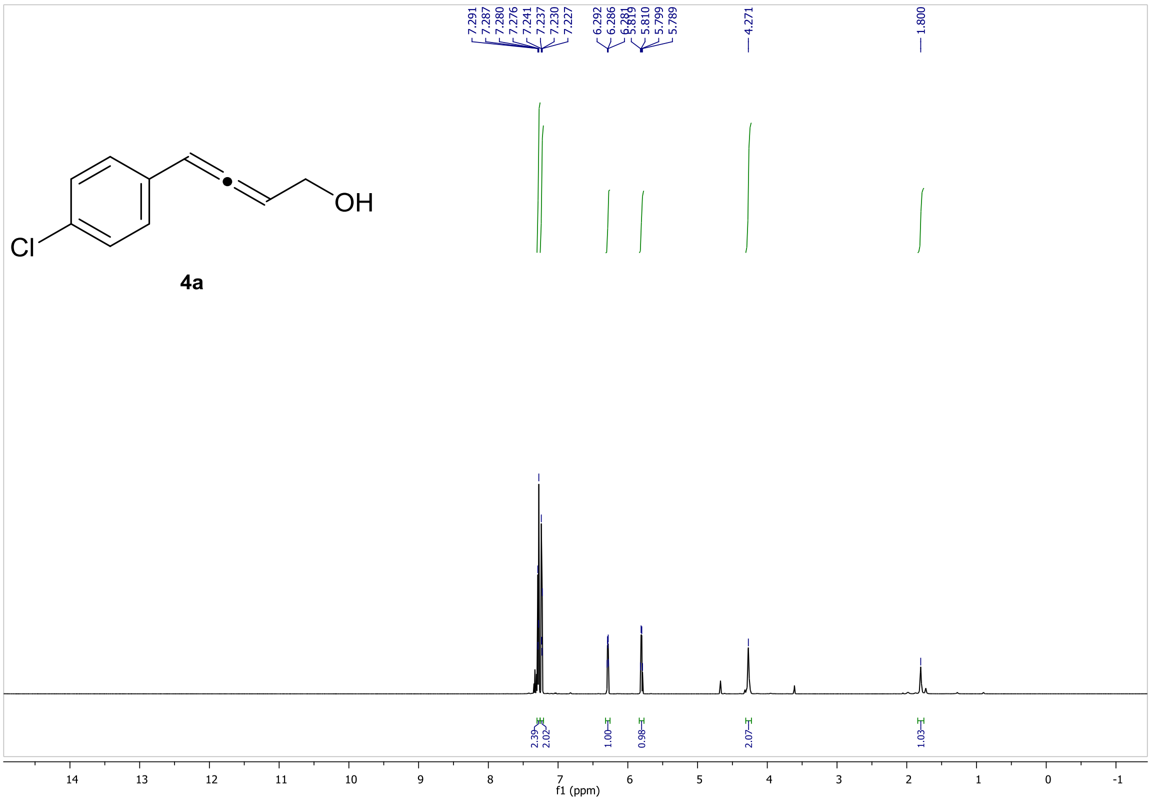

^{13}C NMR, 150 MHz, $CDCl_3$:

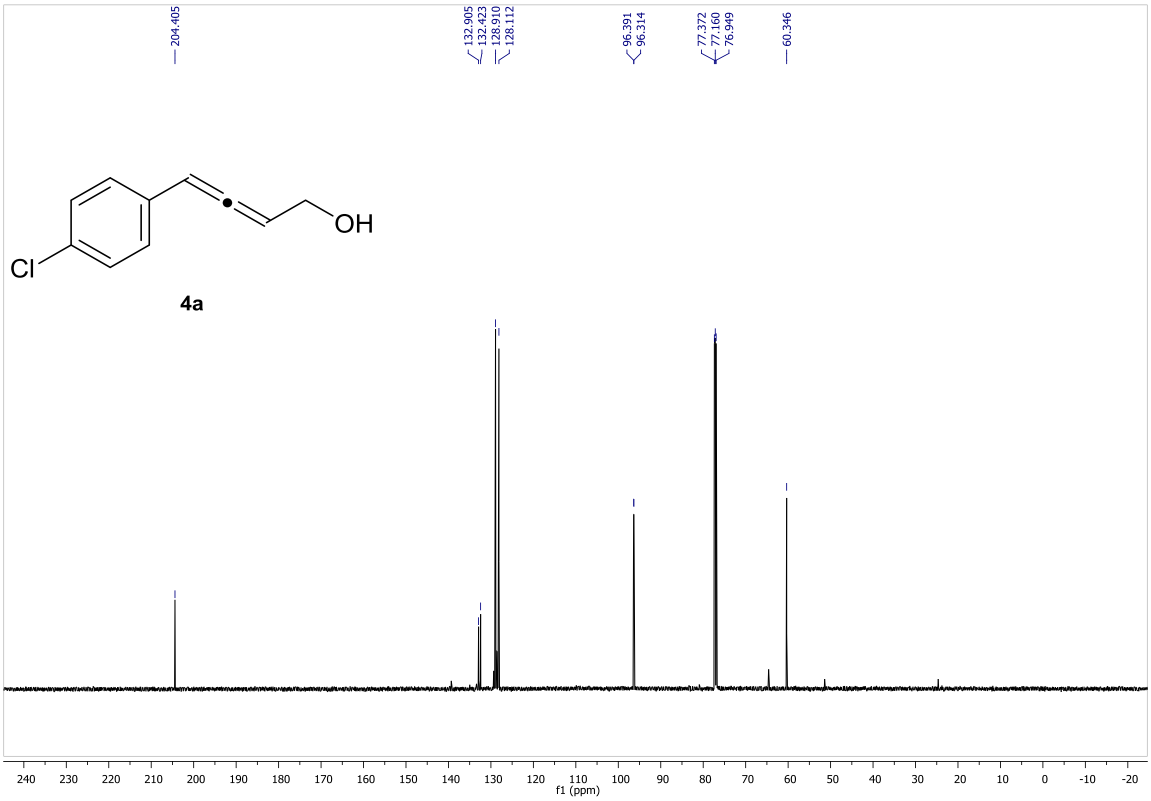

4-(4-bromophenyl)buta-2,3-dien-1-ol (4b):

^{1}H NMR, 600 MHz, $CDCl_3$:

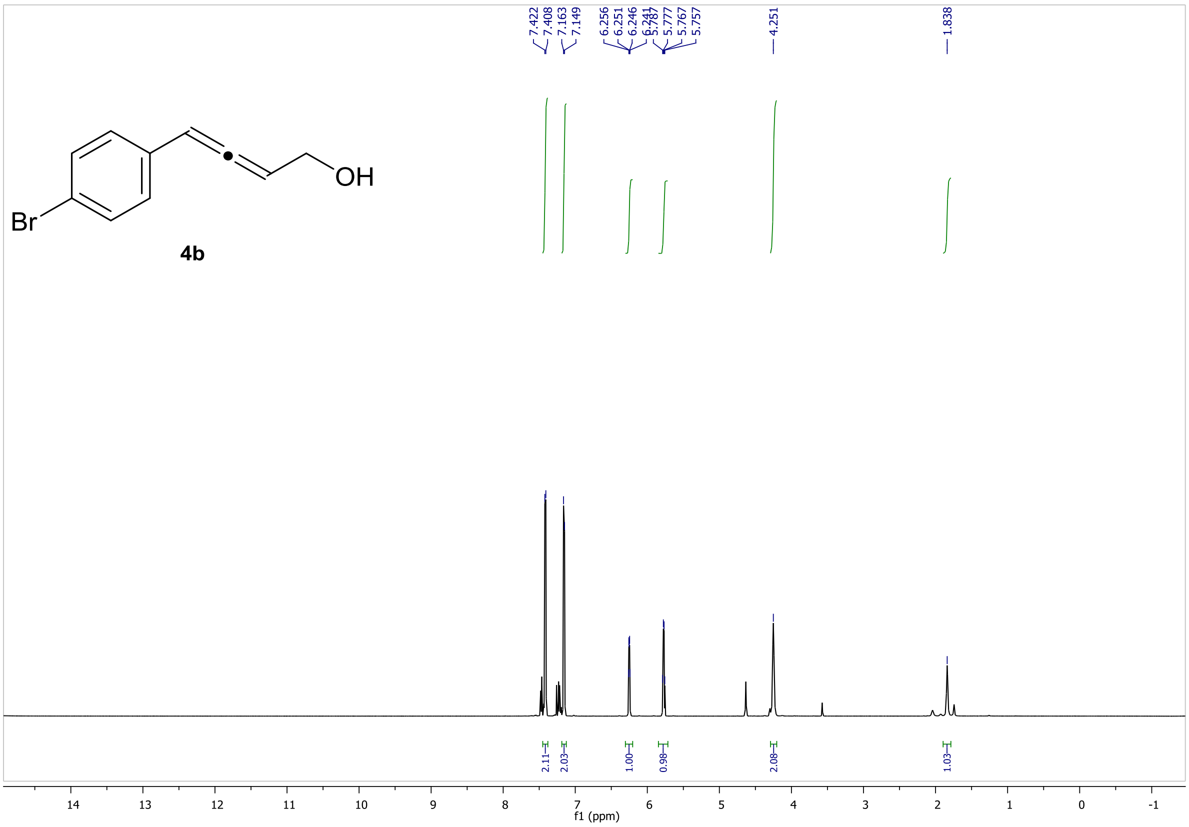

^{13}C NMR, 150 MHz, $CDCl_3$:

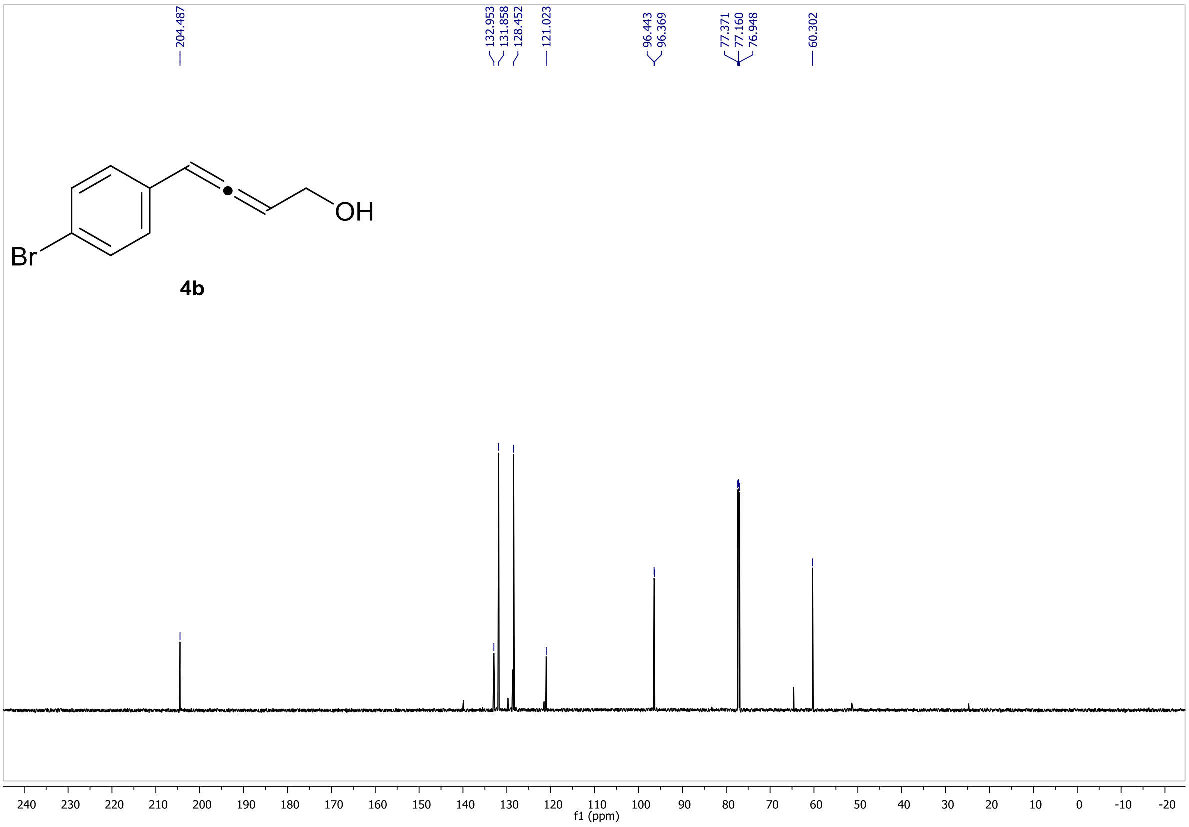

4-(3-methoxyphenyl)buta-2,3-dien-1-ol (4c):

^{1}H NMR, 600 MHz, $CDCl_3$:

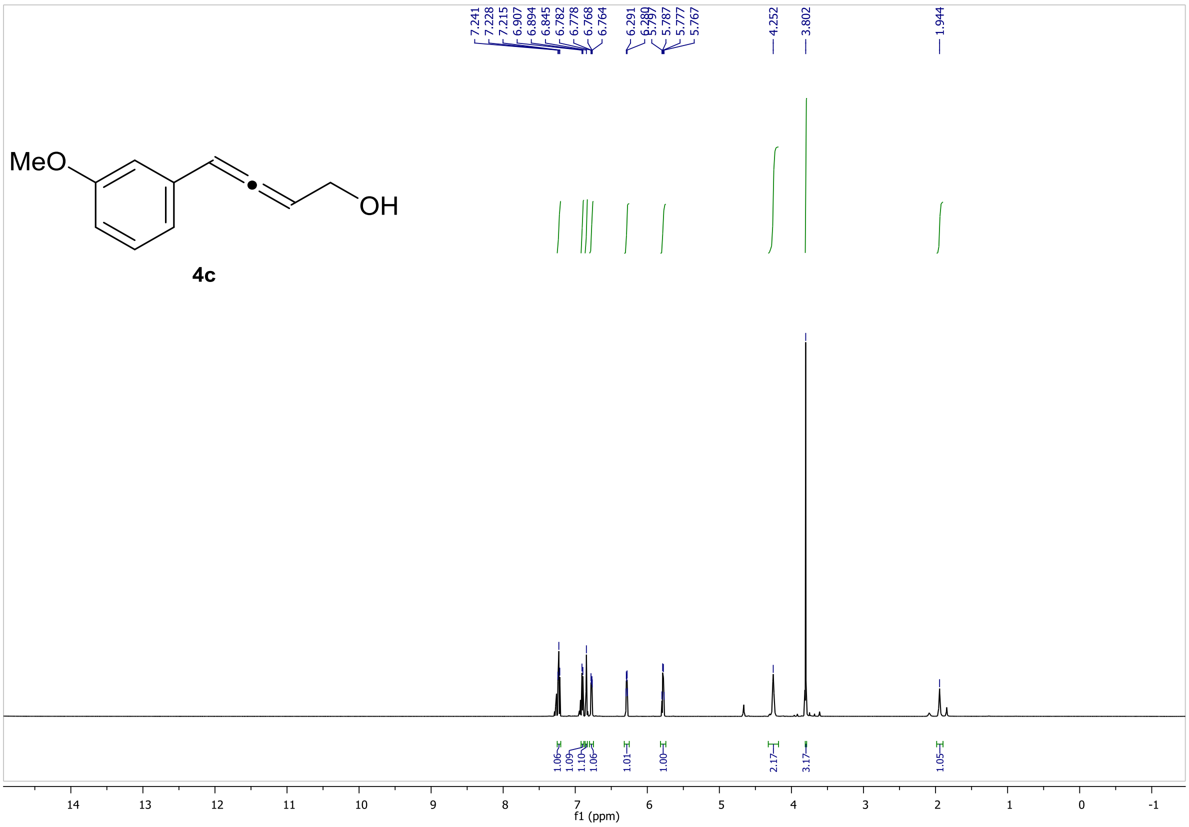

^{13}C NMR, 150 MHz, $CDCl_3$:

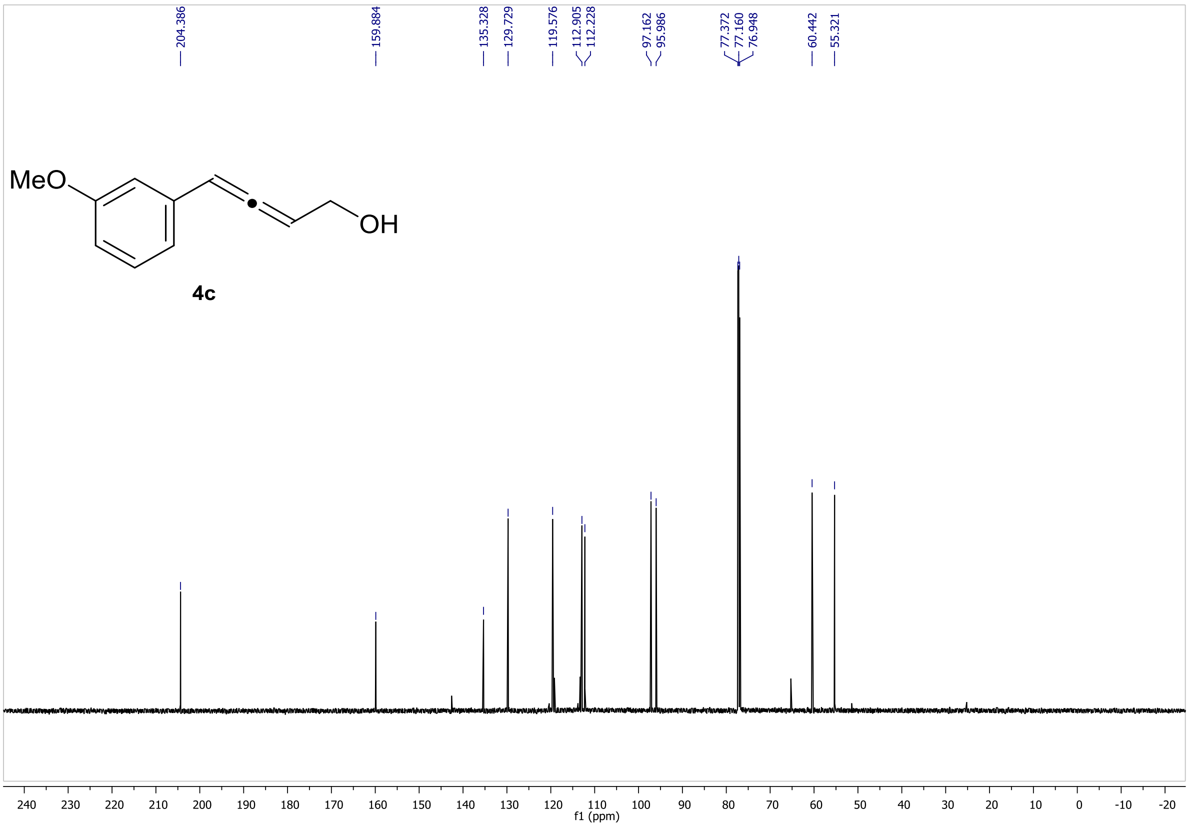

N-(4-(4-chlorophenyl)buta-2,3-dien-1-yl)-4-methylbenzenesulfonamide (4d):

^{1}H NMR, 400 MHz, $CDCl_3$:

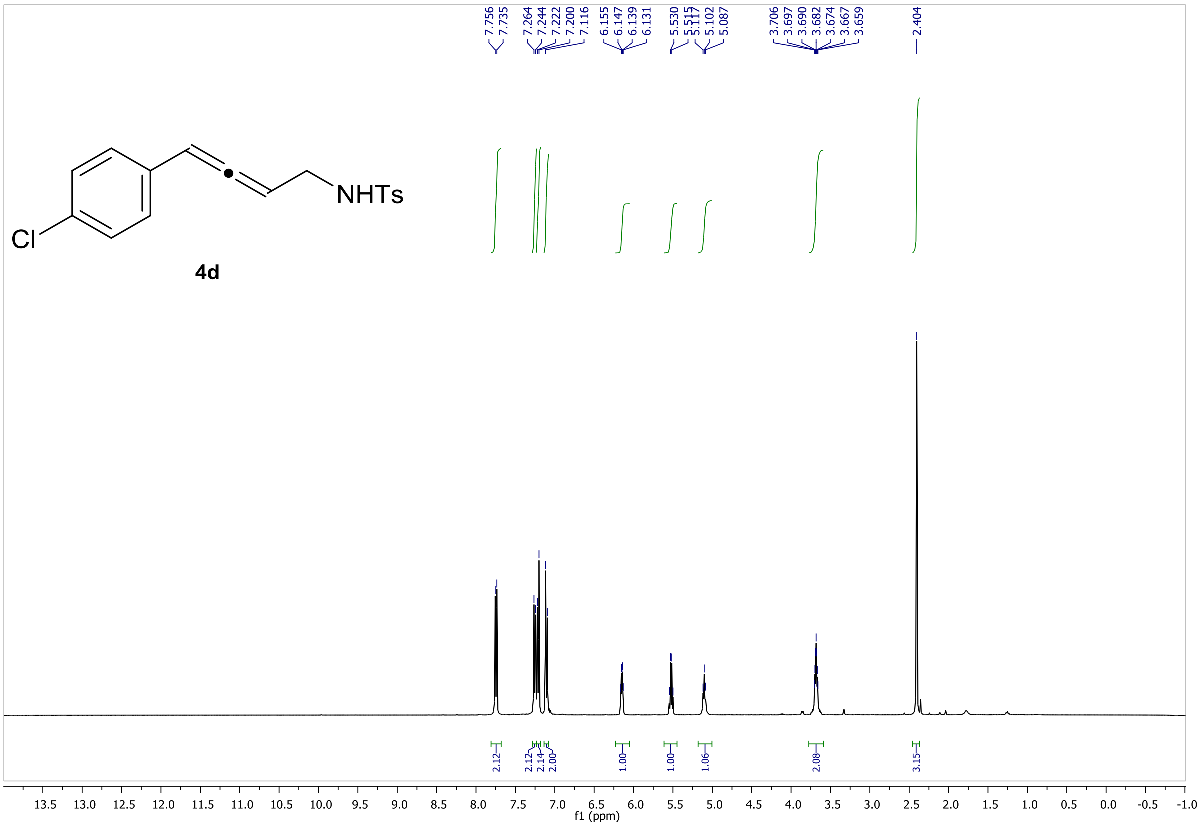

^{13}C NMR, 100 MHz, $CDCl_3$:

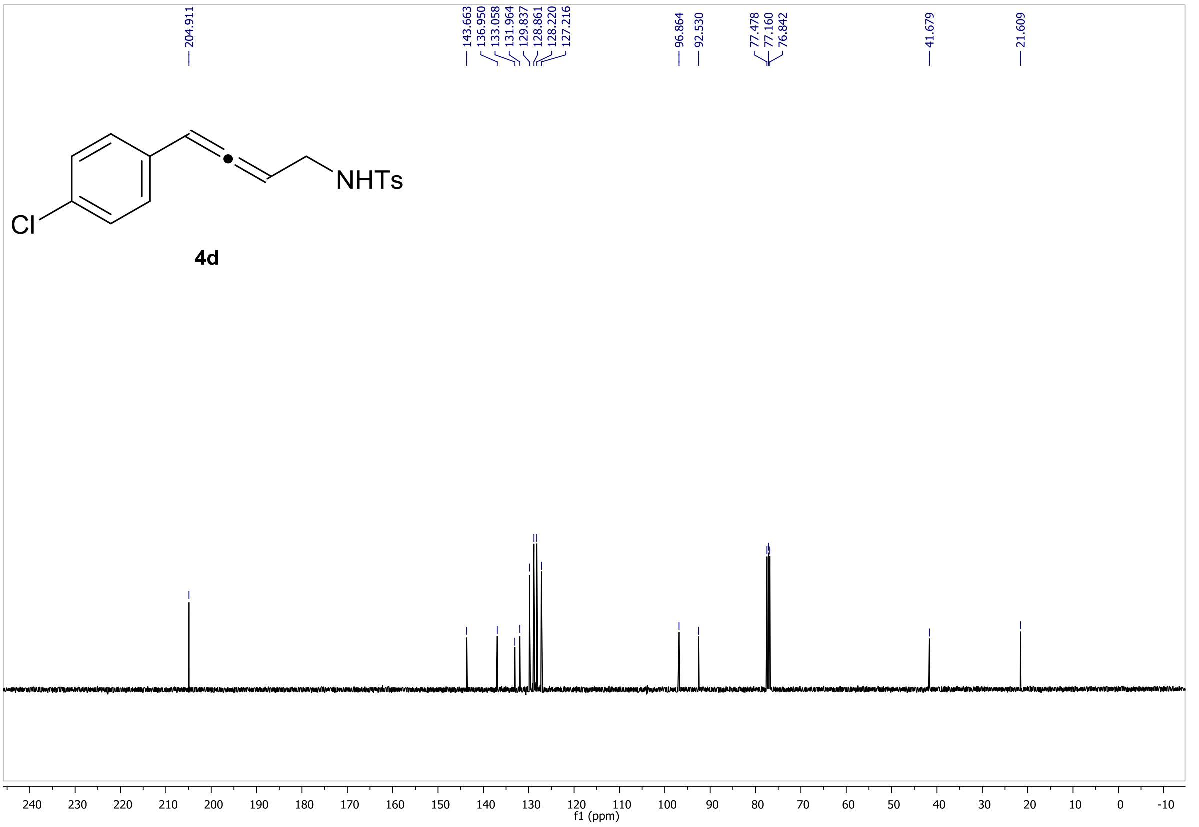

N-(4-(4-bromophenyl)buta-2,3-dien-1-yl)-4-methylbenzenesulfonamide (4e):

^{1}H NMR, 600 MHz, $CDCl_3$:

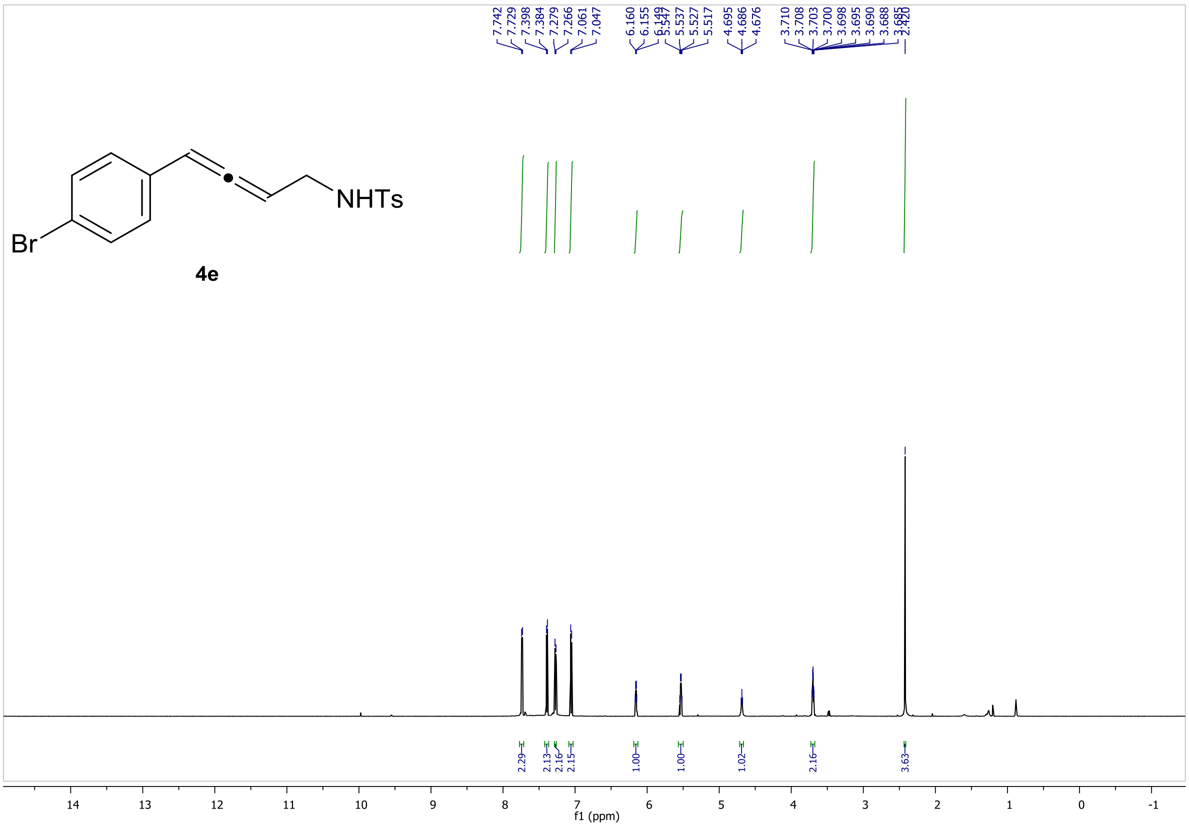

^{13}C NMR, 150 MHz, $CDCl_3$:

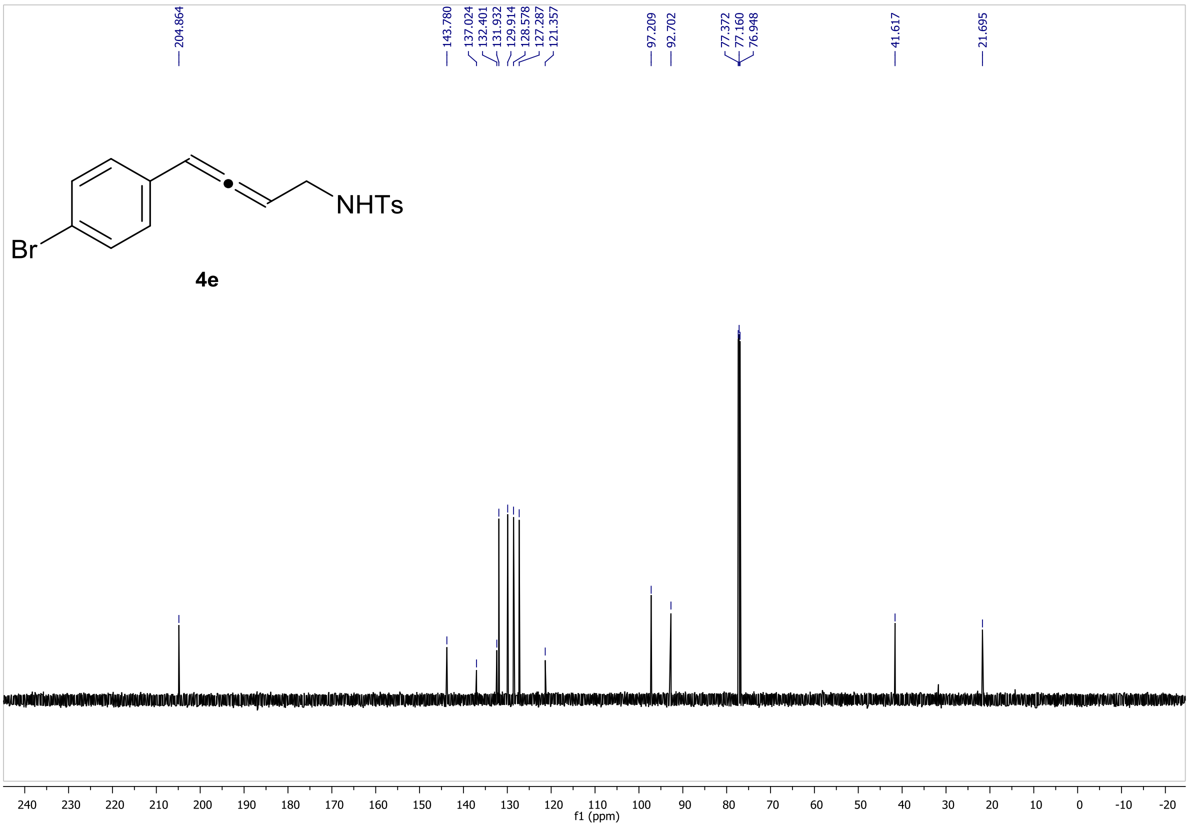

***N*-(4-(3-methoxyphenyl)buta-2,3-dien-1-yl)-4-methylbenzenesulfonamide (4f):**

^{1}H NMR, 600 MHz, $CDCl_3$:

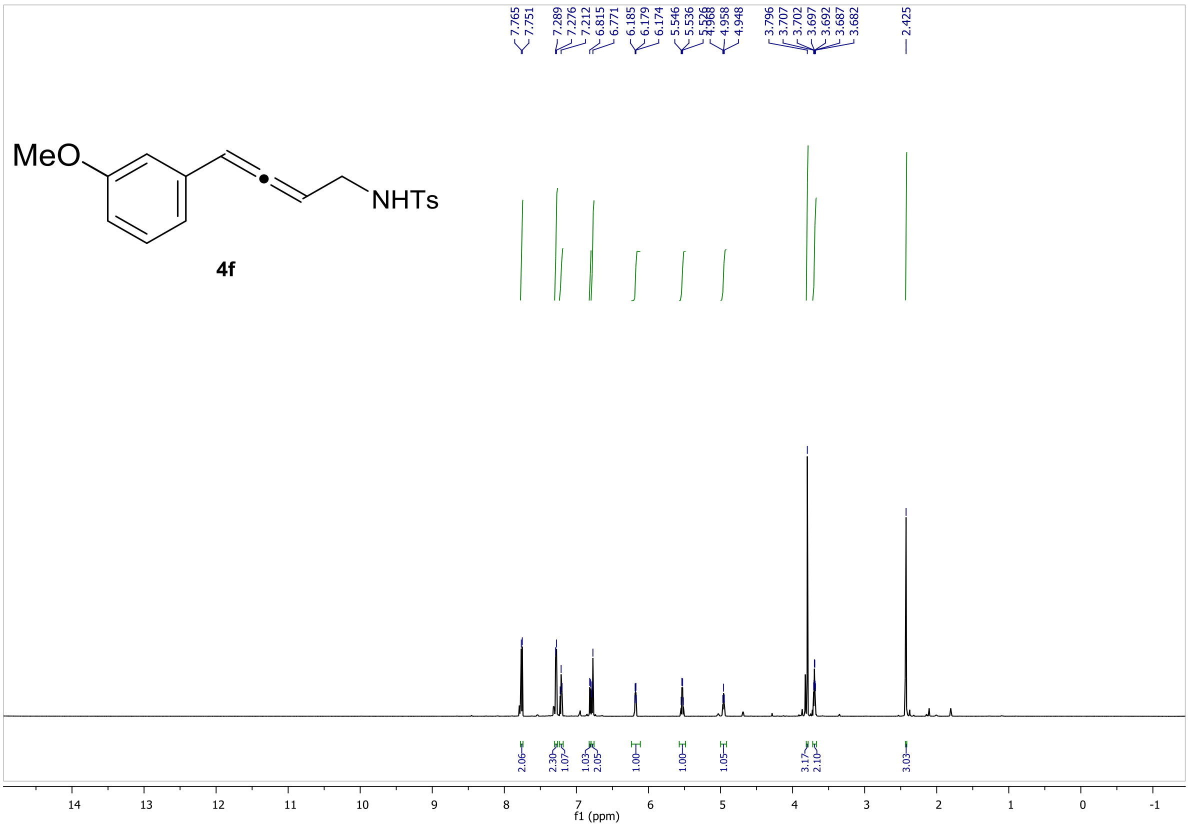

^{13}C NMR, 150 MHz, $CDCl_3$:

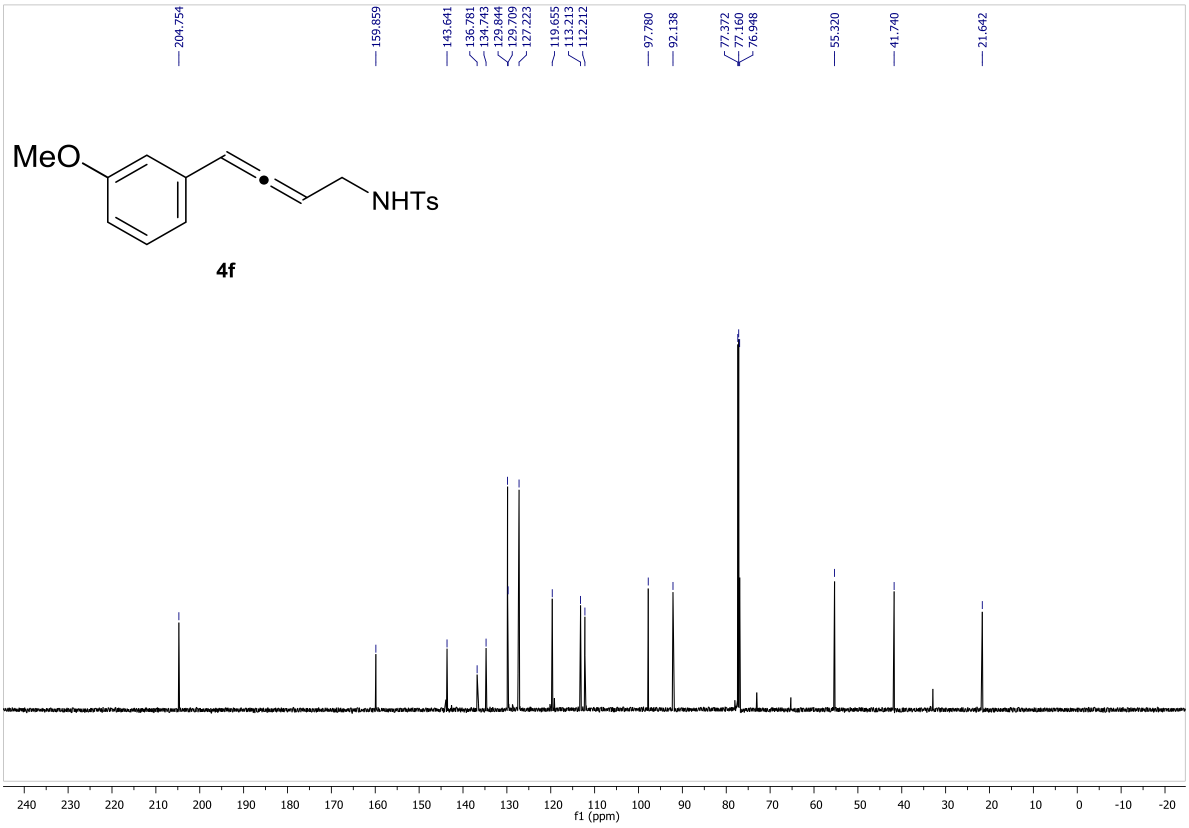

4-(4-hydroxybuta-1,2-dien-1-yl)benzonitrile (4g):

^{1}H NMR, 400 MHz, $CDCl_3$:

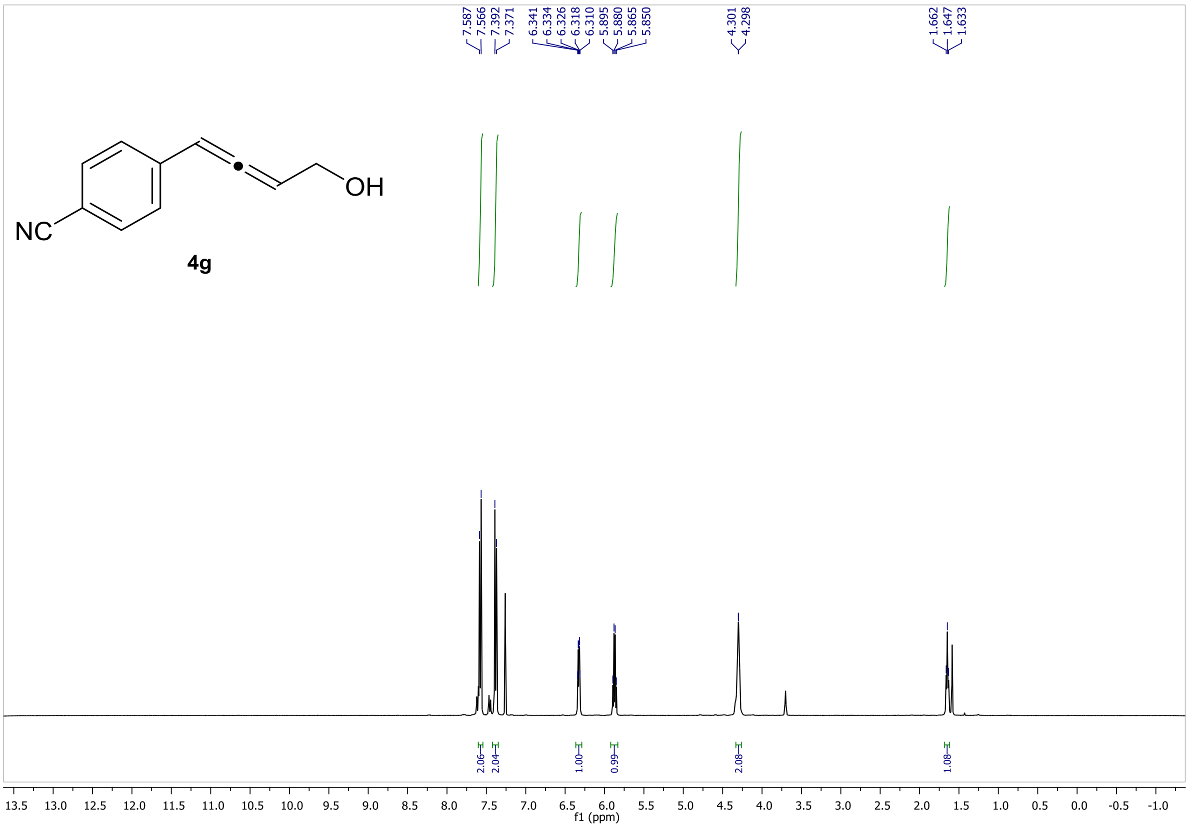

^{13}C NMR, 100 MHz, $CDCl_3$:

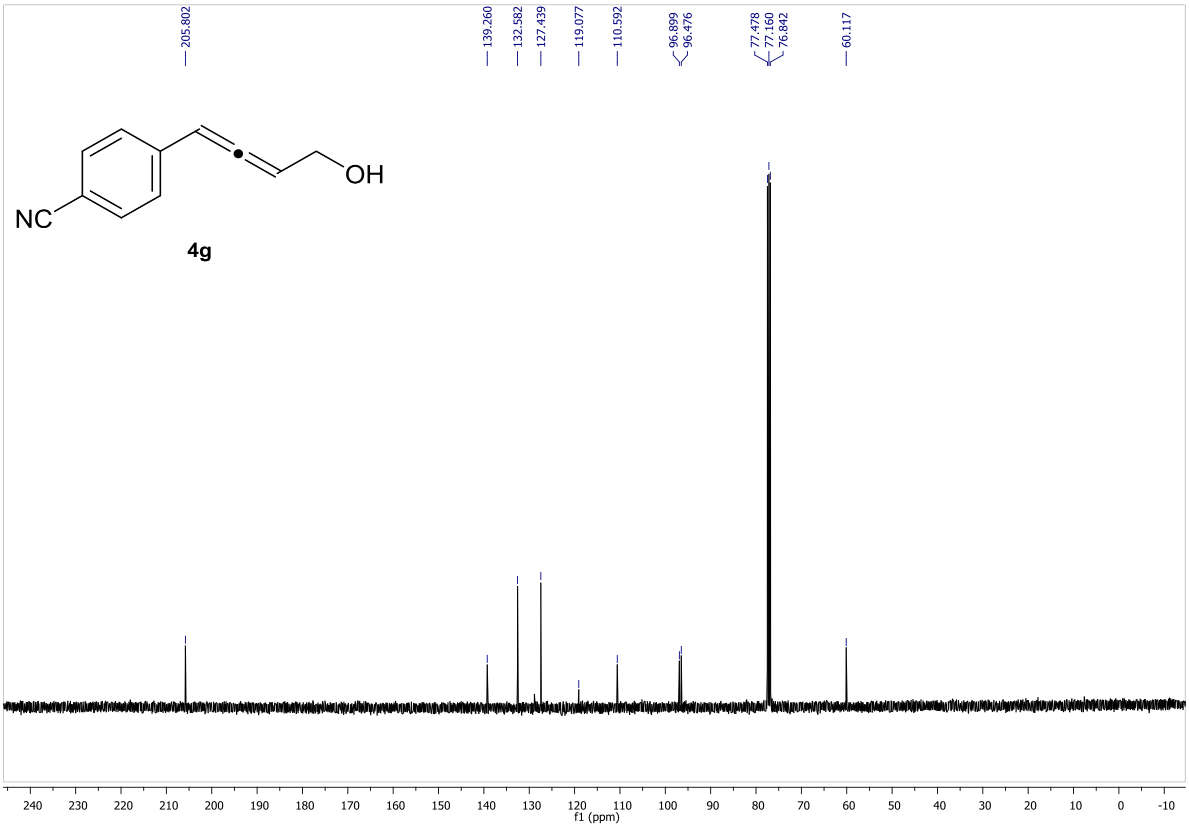

(*E*)-6-phenylhexa-2,3,5-trien-1-ol (4h):

^{1}H NMR, 600 MHz, CDCl$_3$:

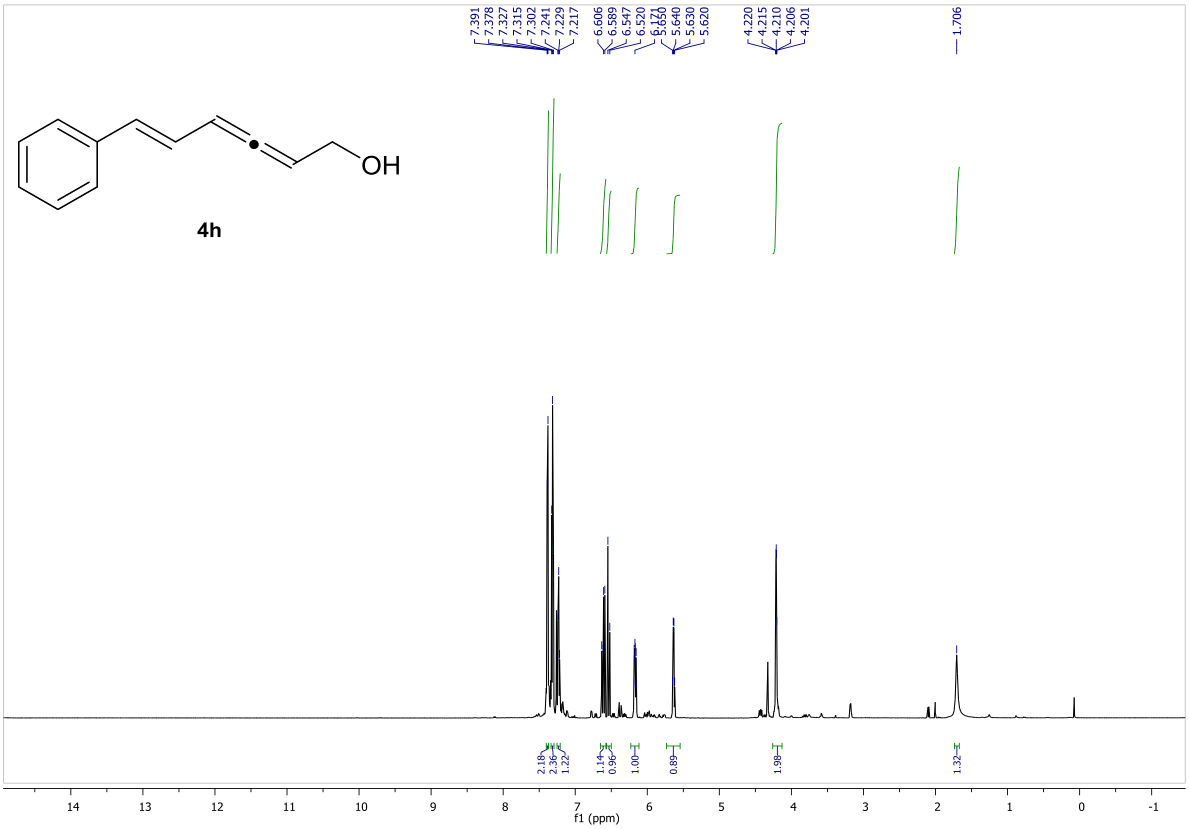

^{13}C NMR, 150 MHz, CDCl$_3$:

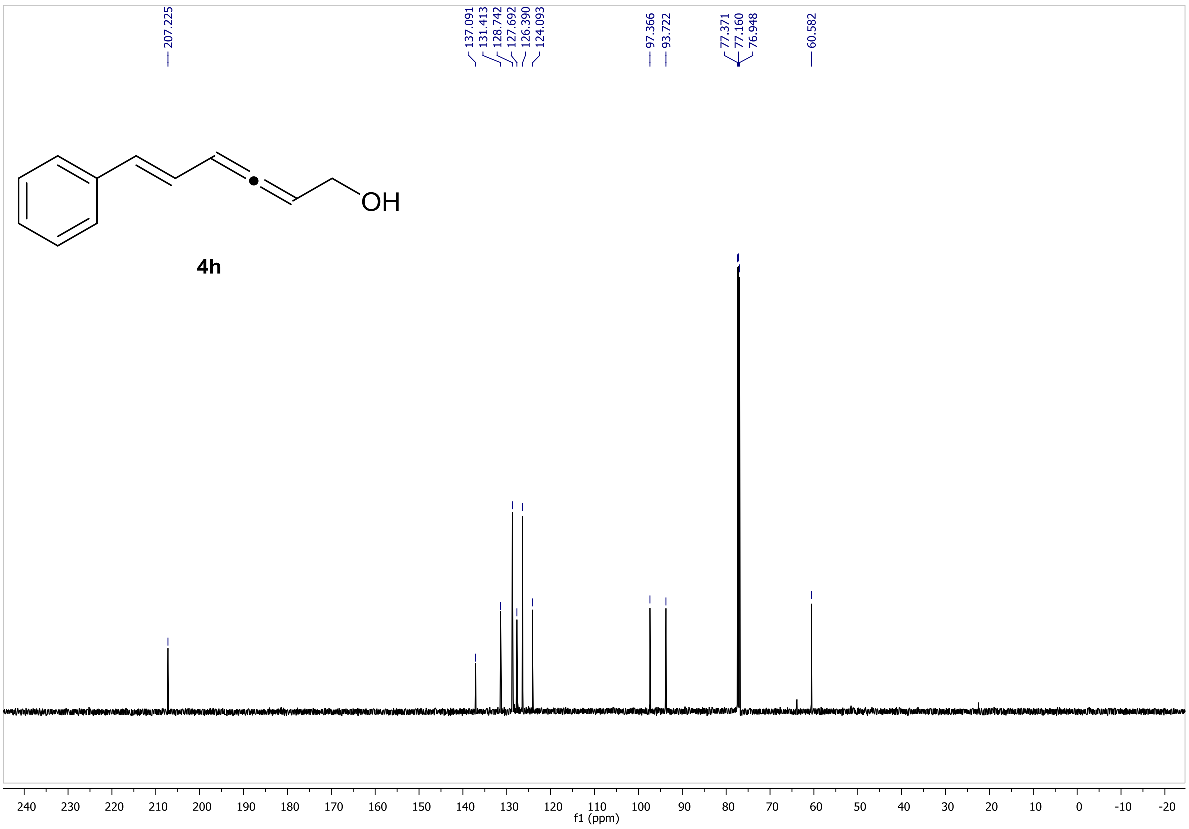

1-chloro-4-(3-cyclopentylpropa-1,2-dien-1-yl)benzene (4i):

^{1}H NMR, 600 MHz, $CDCl_3$:

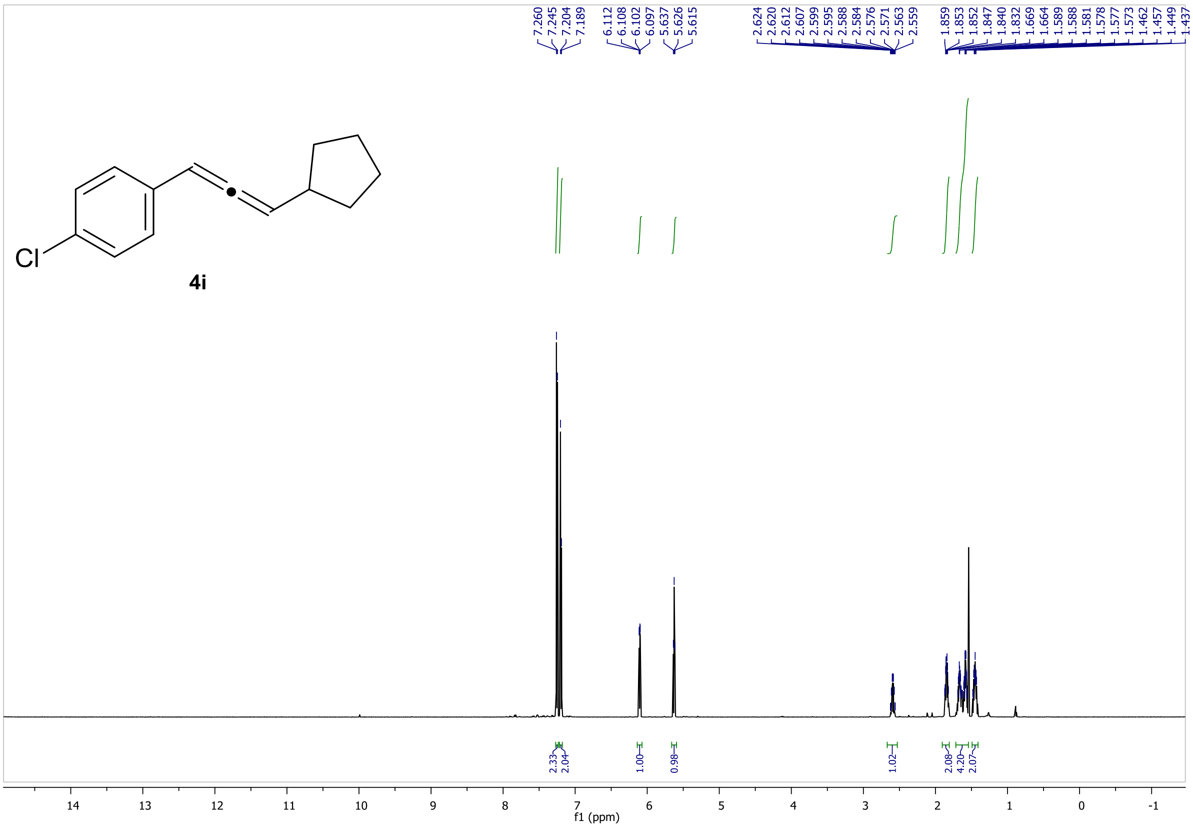

^{13}C NMR, 150 MHz, $CDCl_3$:

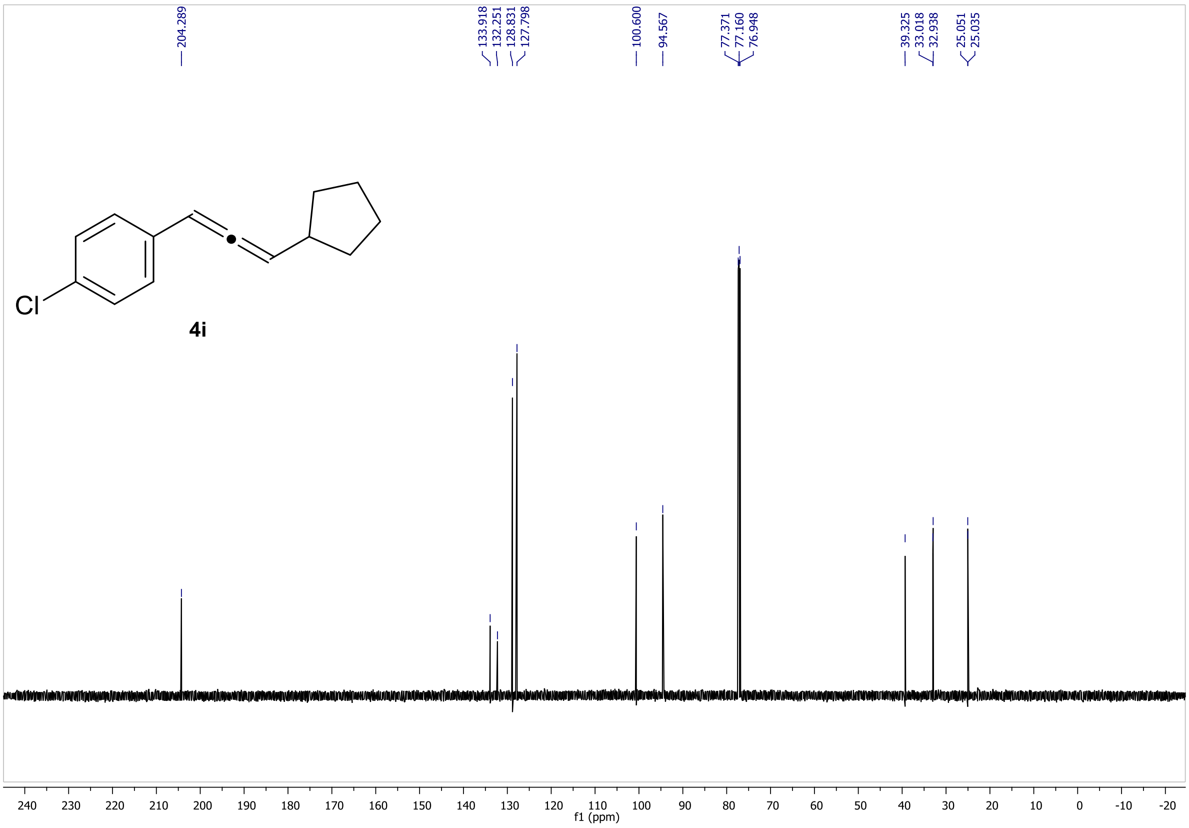

1-chloro-4-(3-cyclopropylpropa-1,2-dien-1-yl)benzene (4j):

^{1}H NMR, 600 MHz, $CDCl_3$:

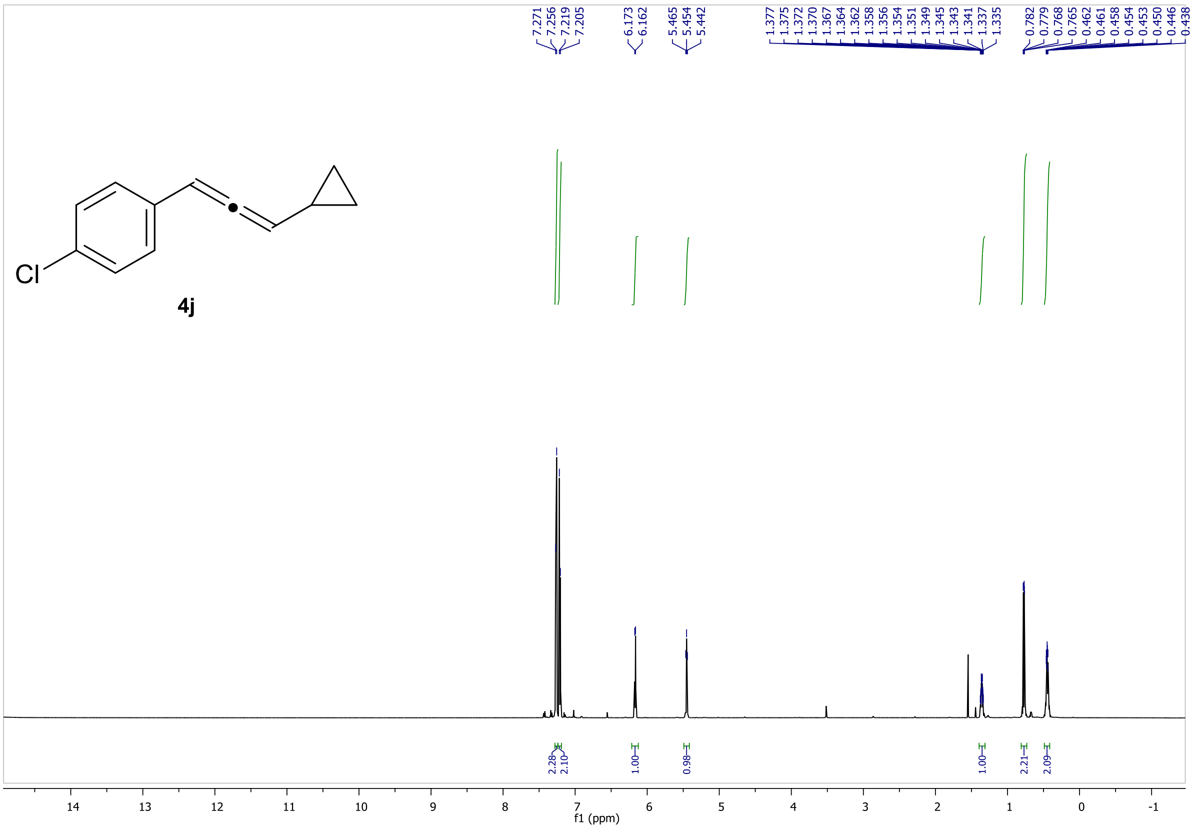

^{13}C NMR, 150 MHz, $CDCl_3$:

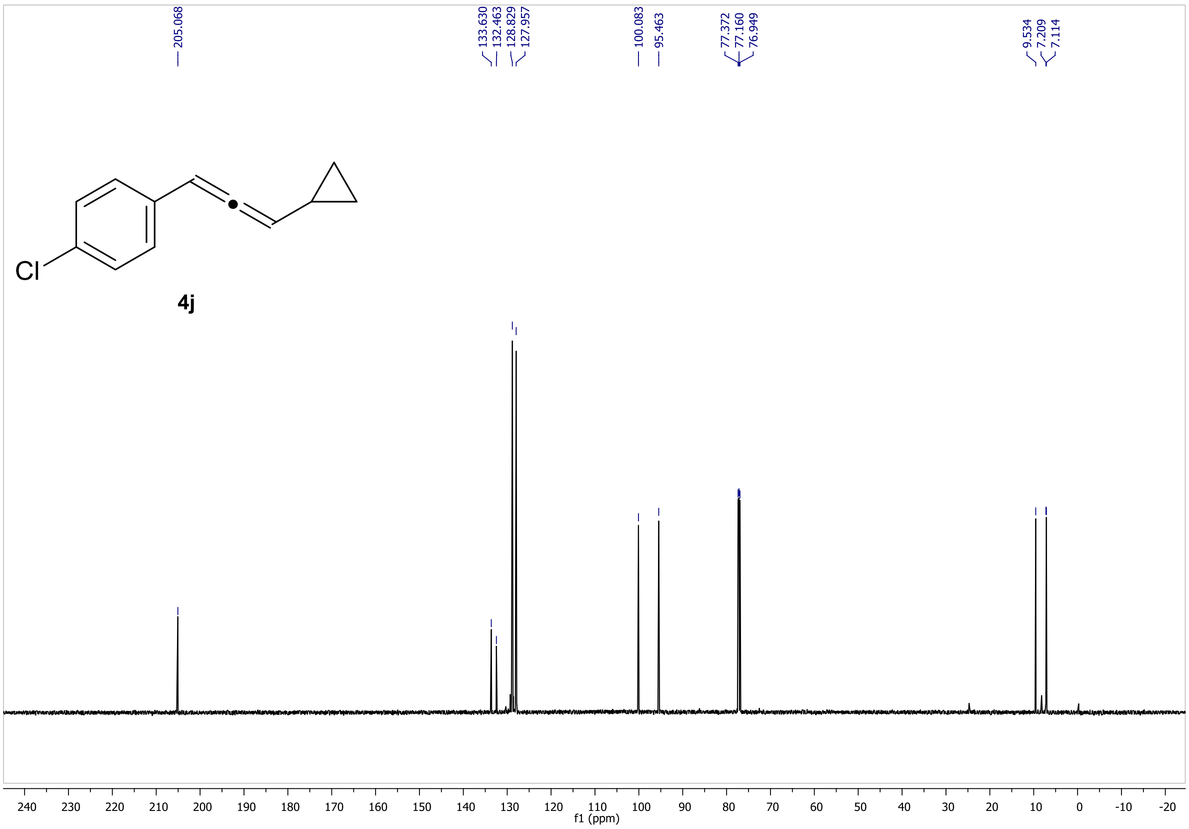

1-(5-bromopenta-1,2-dien-1-yl)-4-chlorobenzene (4k):

^{1}H NMR, 600 MHz, $CDCl_3$:

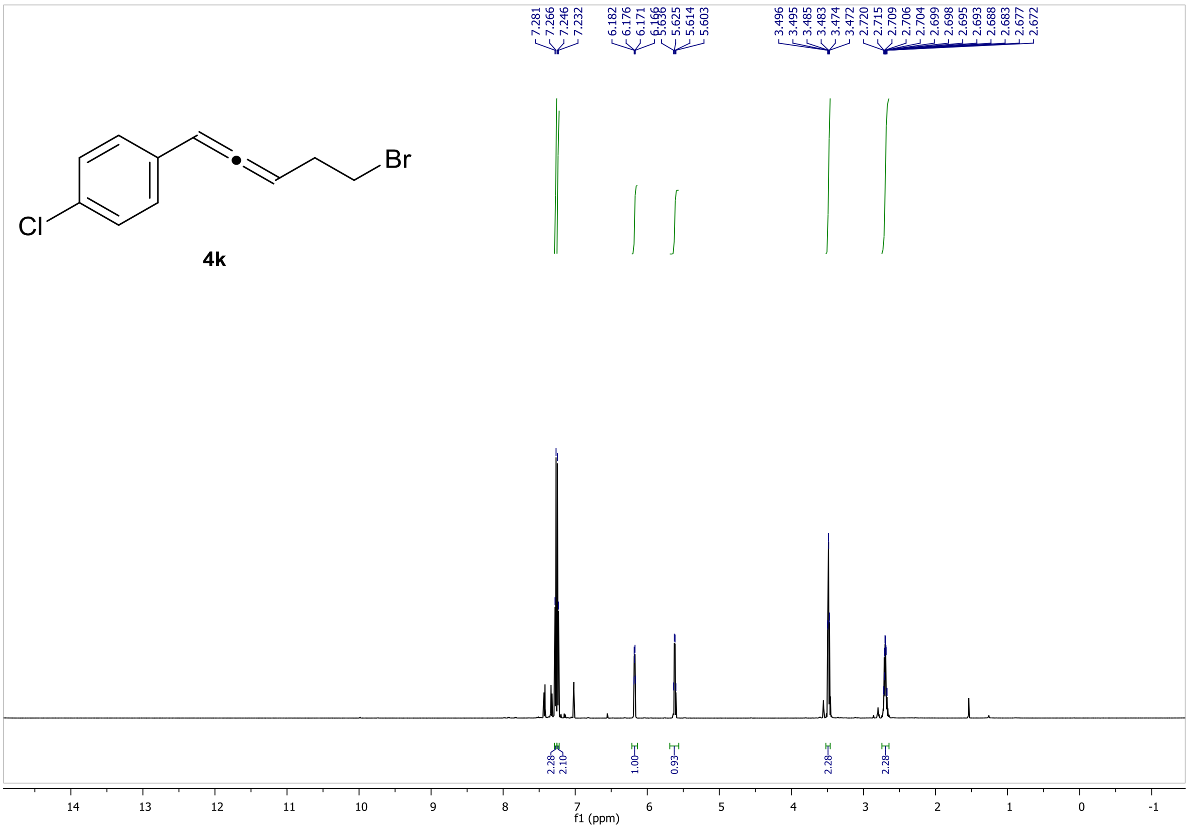

^{13}C NMR, 150 MHz, $CDCl_3$:

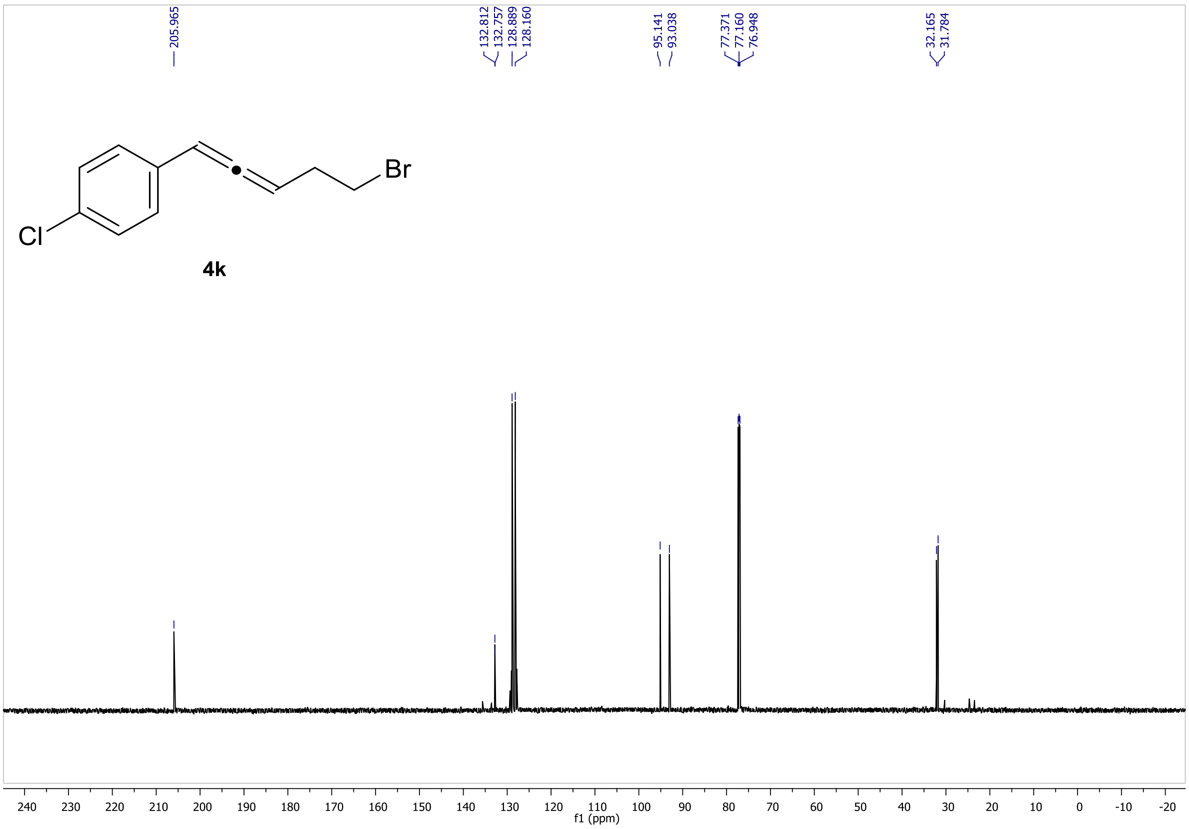

1-bromo-4-(5-bromopenta-1,2-dien-1-yl)benzene (4l):

^{1}H NMR, 600 MHz, CDCl$_3$:

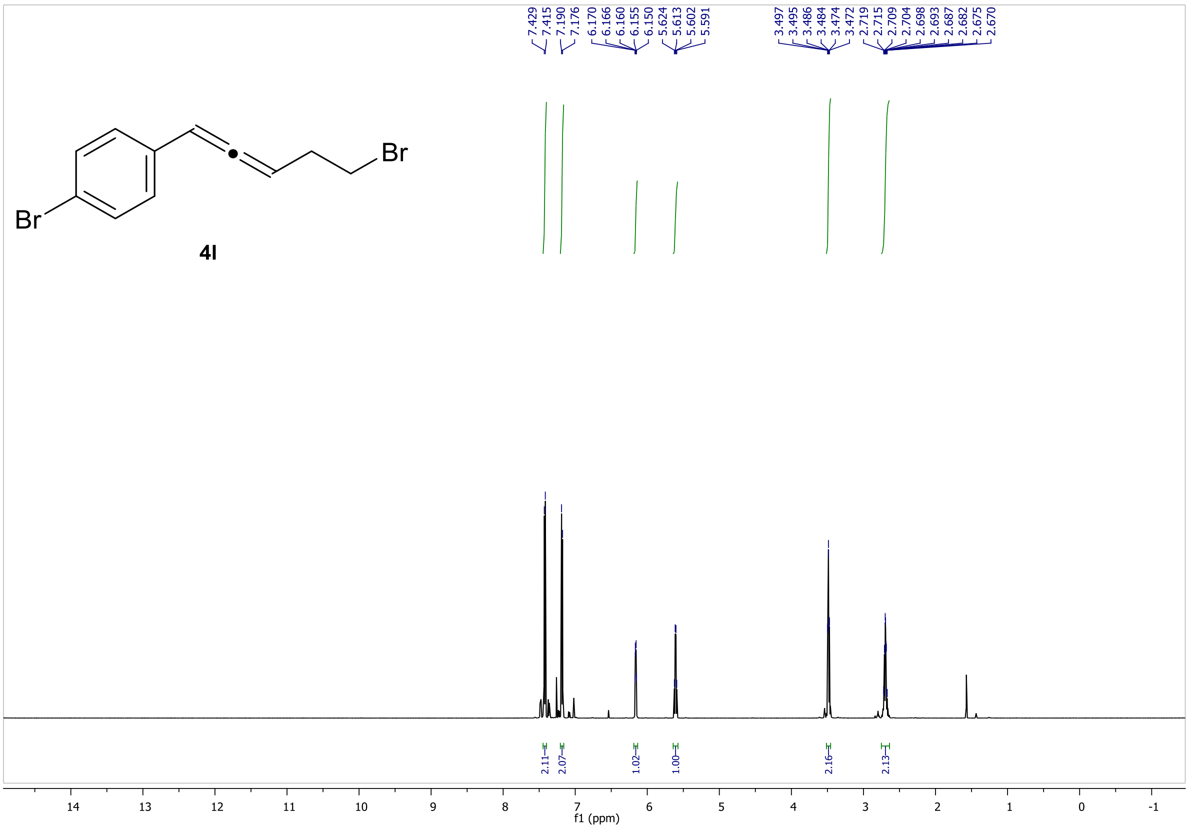

^{13}C NMR, 150 MHz, CDCl$_3$:

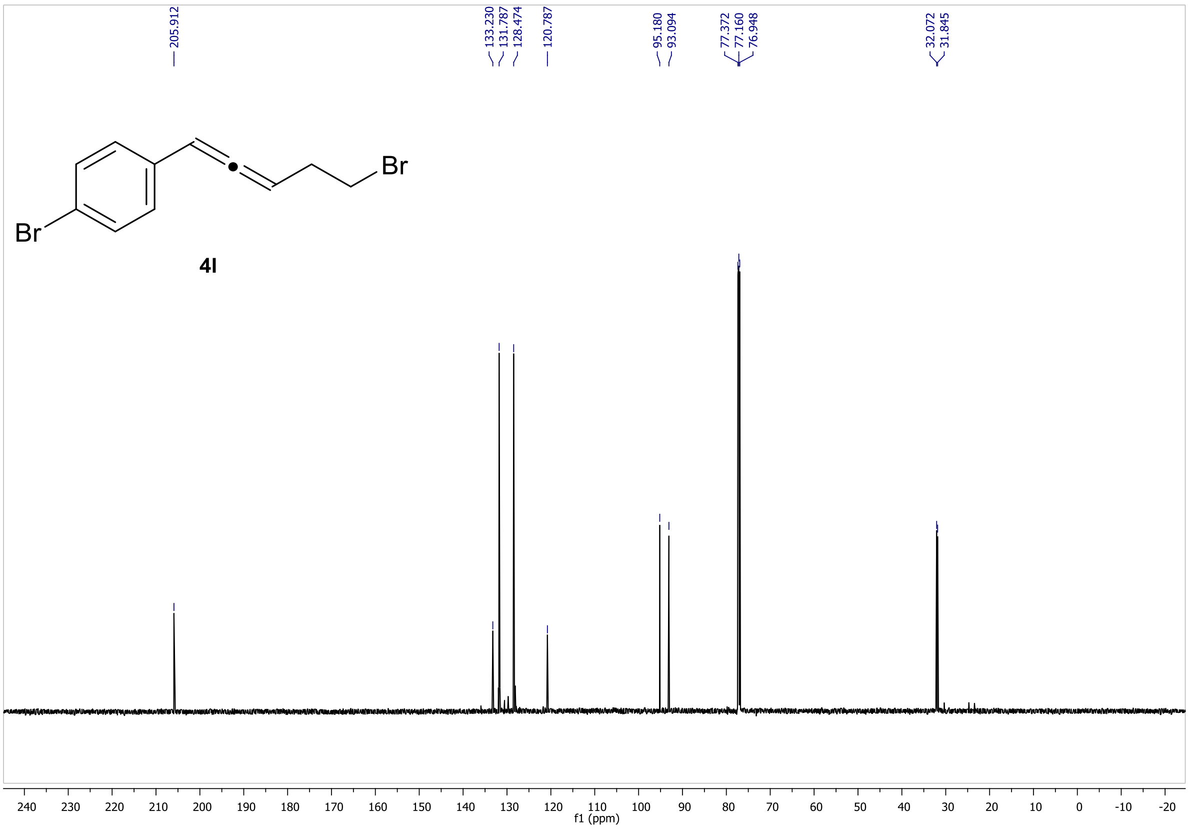

1-chloro-4-(3-phenylpropa-1,2-dien-1-yl)benzene (4m):

^{1}H NMR, 600 MHz, $CDCl_3$:

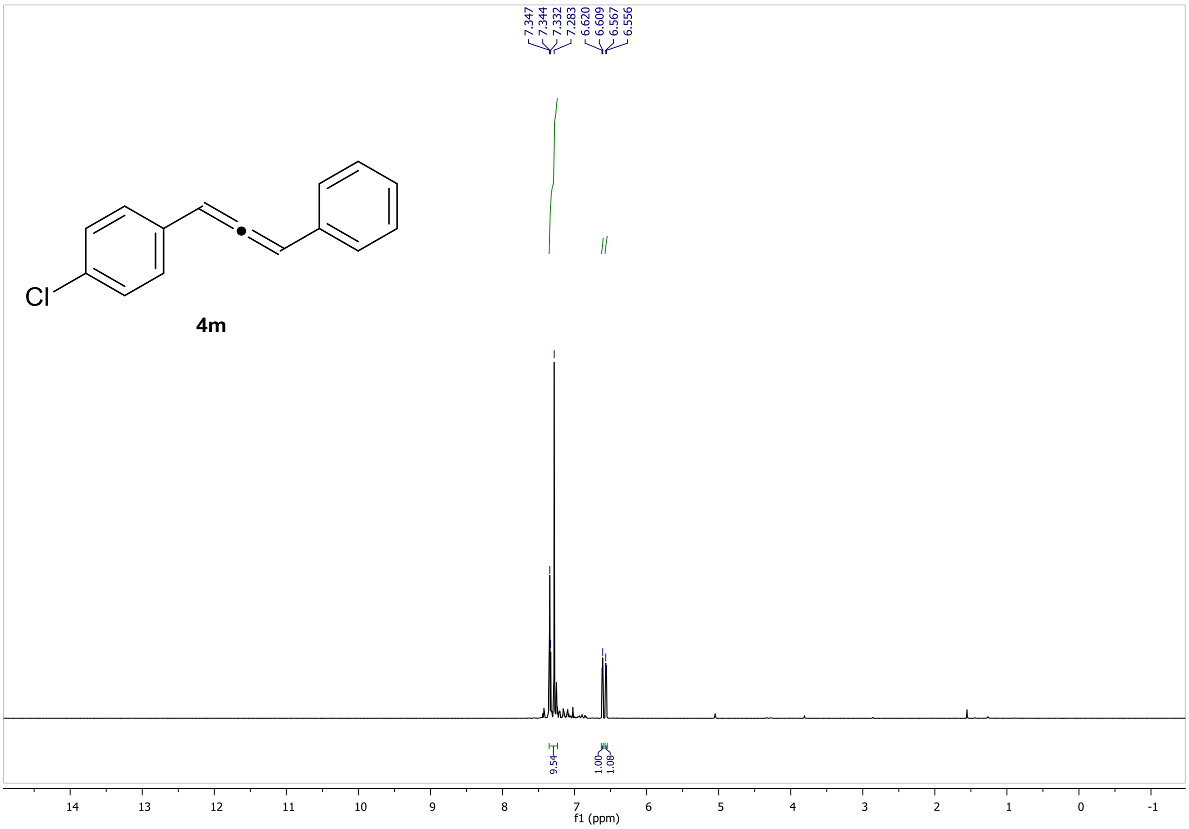

^{13}C NMR, 150 MHz, $CDCl_3$:

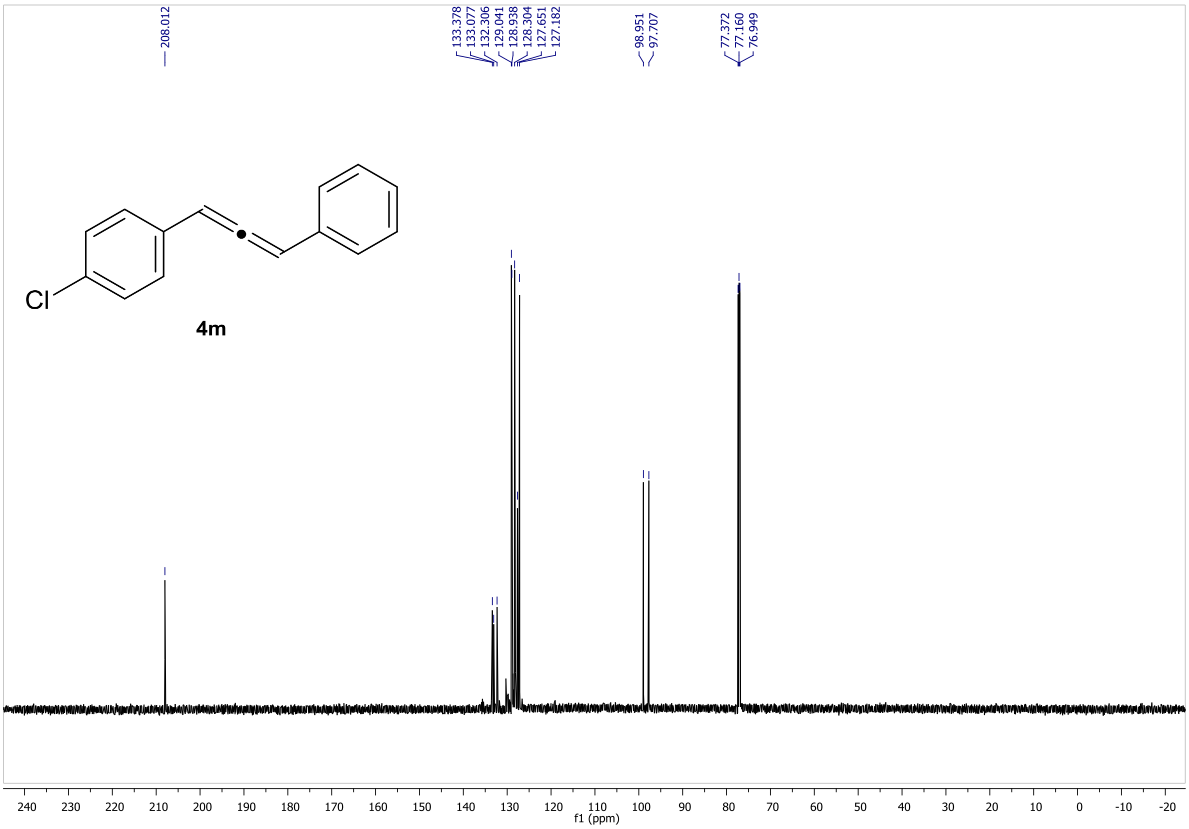

1-chloro-4-(7-iodohepta-1,2-dien-1-yl)benzene (4n):

^{1}H NMR, 600 MHz, $CDCl_3$:

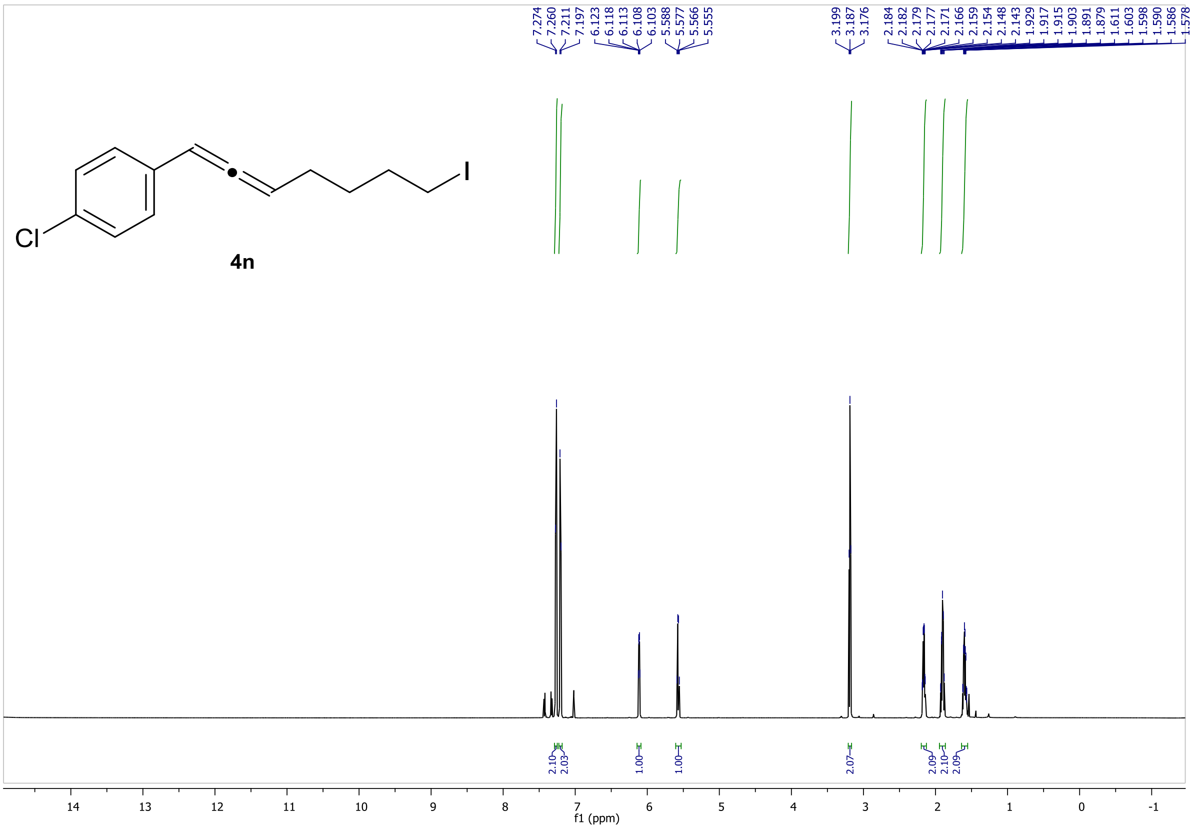

^{13}C NMR, 150 MHz, $CDCl_3$:

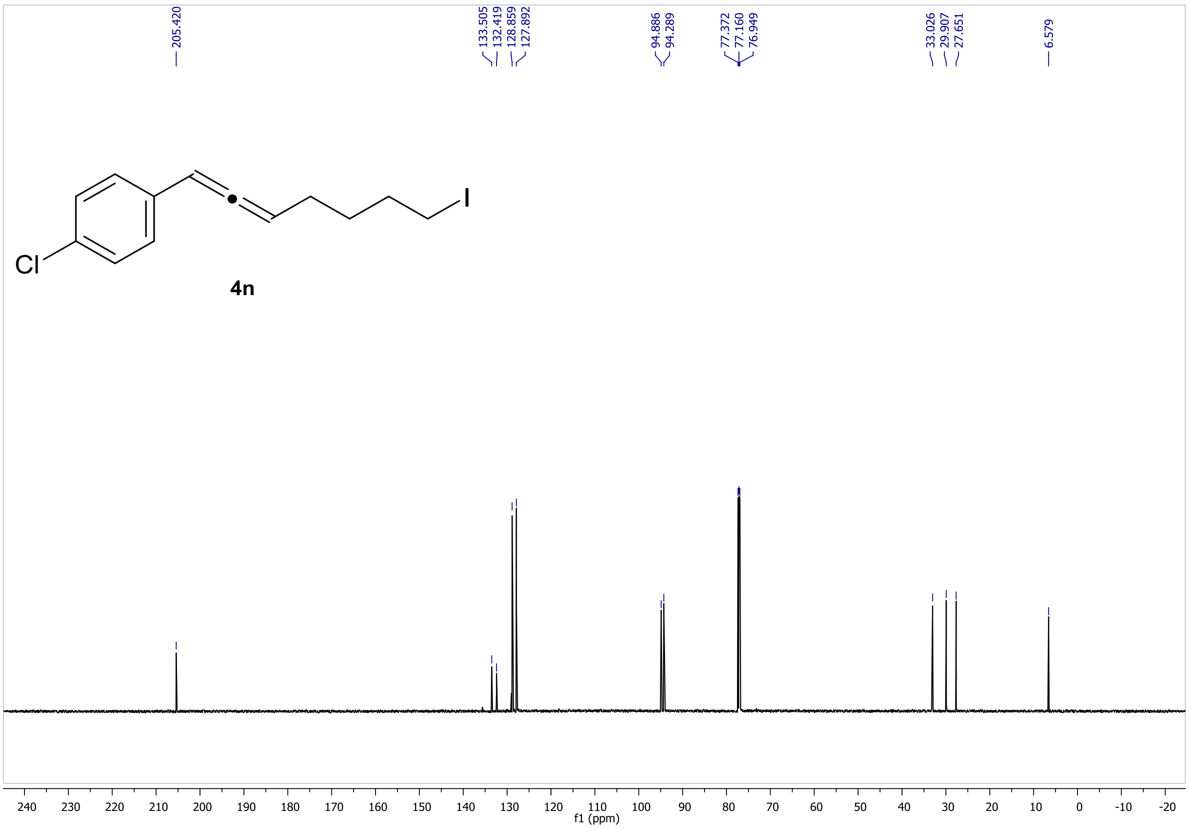

1-chloro-4-(3-(cyclohex-1-en-1-yl)propa-1,2-dien-1-yl)benzene (4o):

^{1}H NMR, 600 MHz, $CDCl_3$:

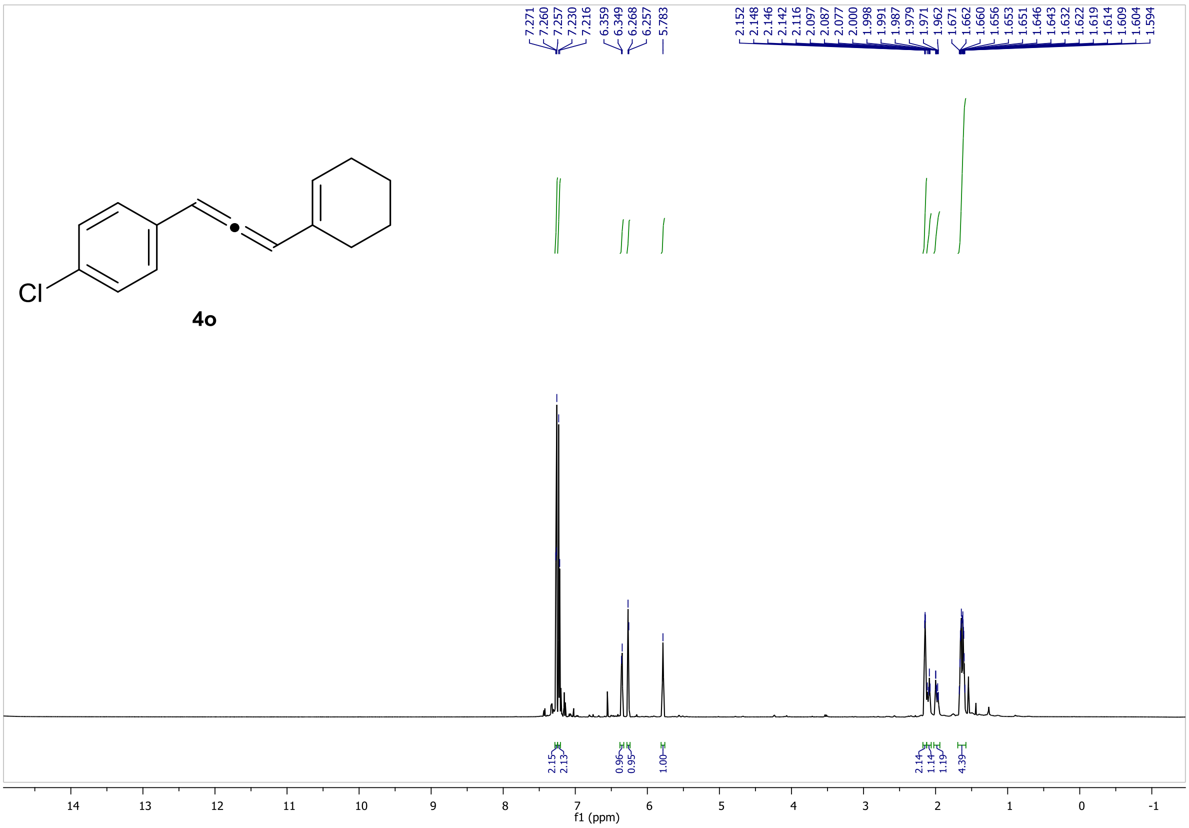

^{13}C NMR, 150 MHz, $CDCl_3$:

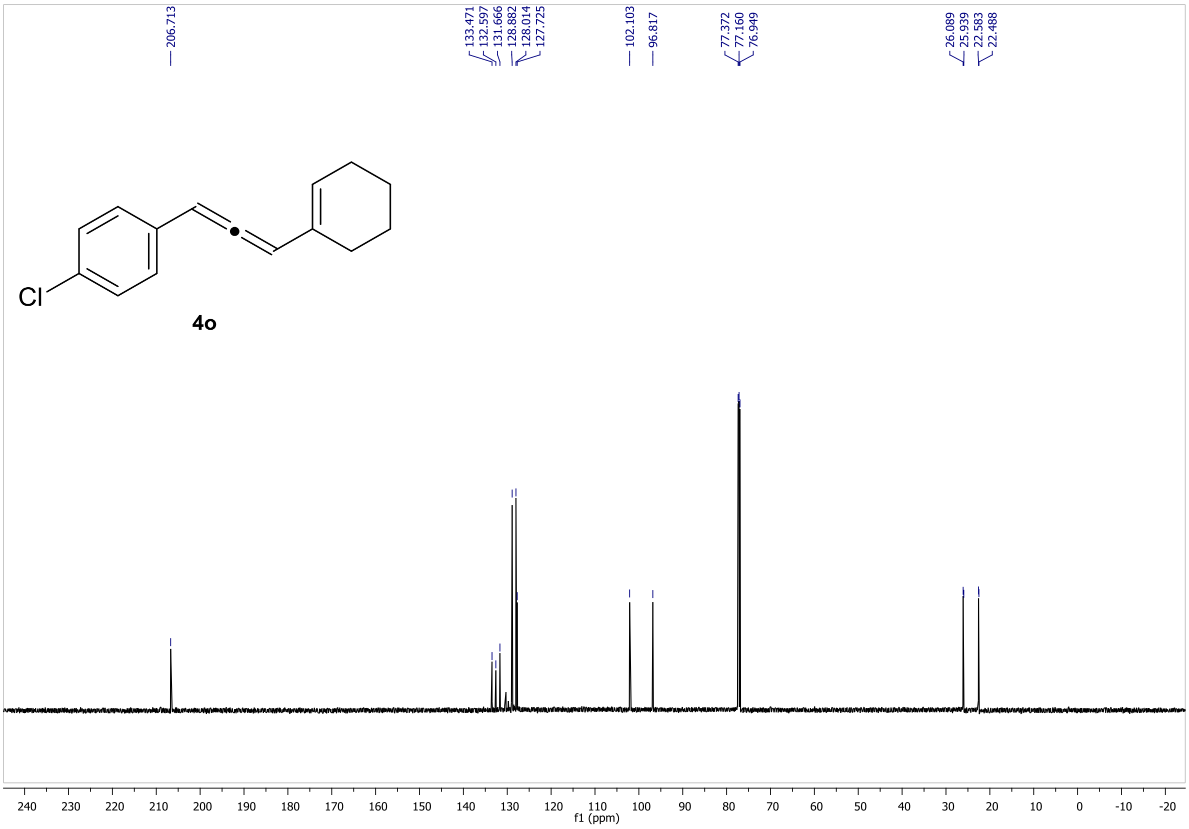

7-(4-chlorophenyl)hepta-5,6-dienenitrile (4p):

^{1}H NMR, 600 MHz, $CDCl_3$:

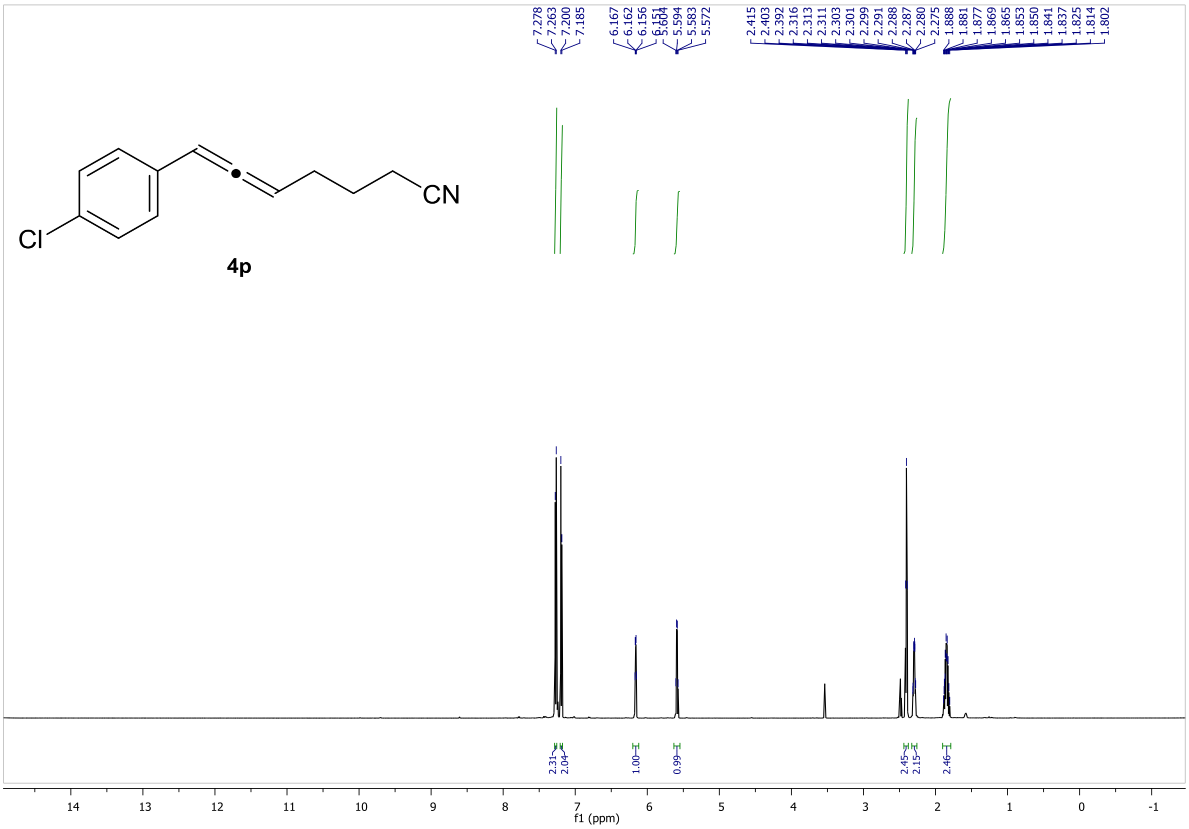

^{13}C NMR, 150 MHz, $CDCl_3$:

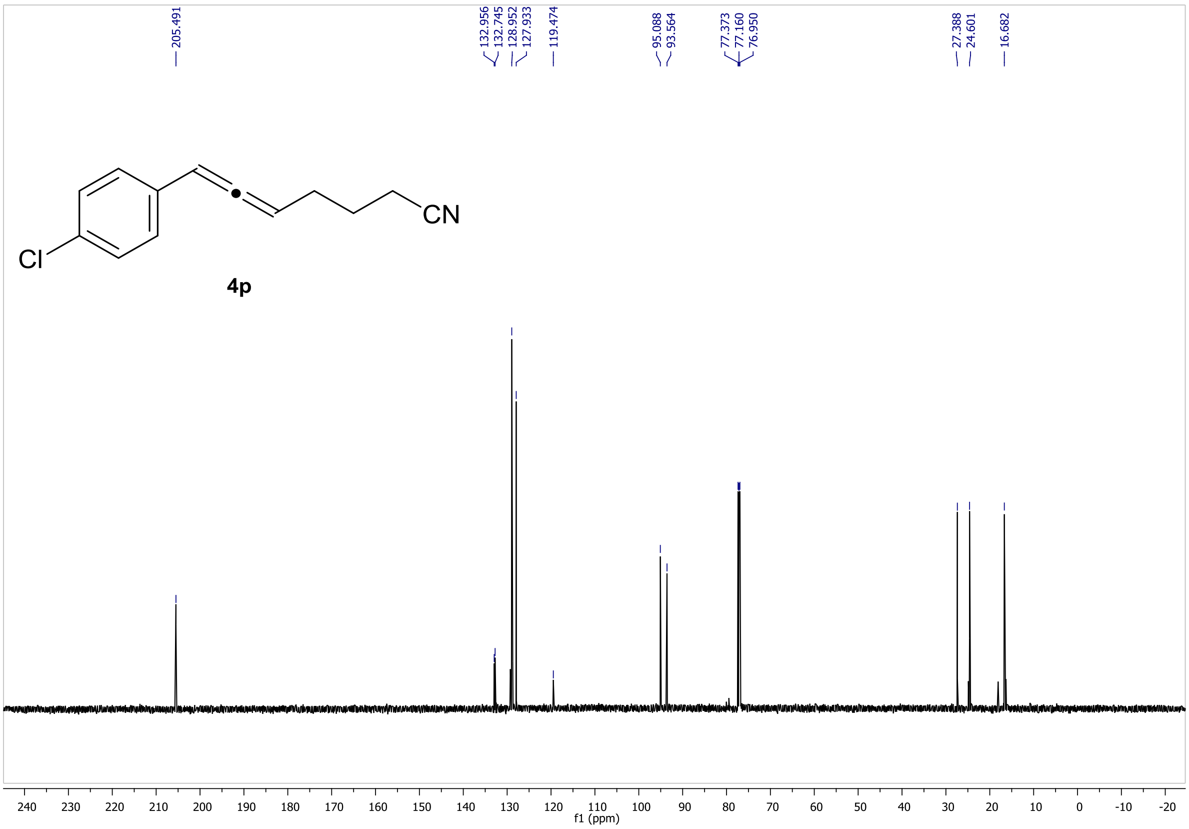

***tert*-butyl (4-(4-chlorophenyl)buta-2,3-dien-1-yl)carbamate (4q):**

^{1}H NMR, 600 MHz, $CDCl_3$:

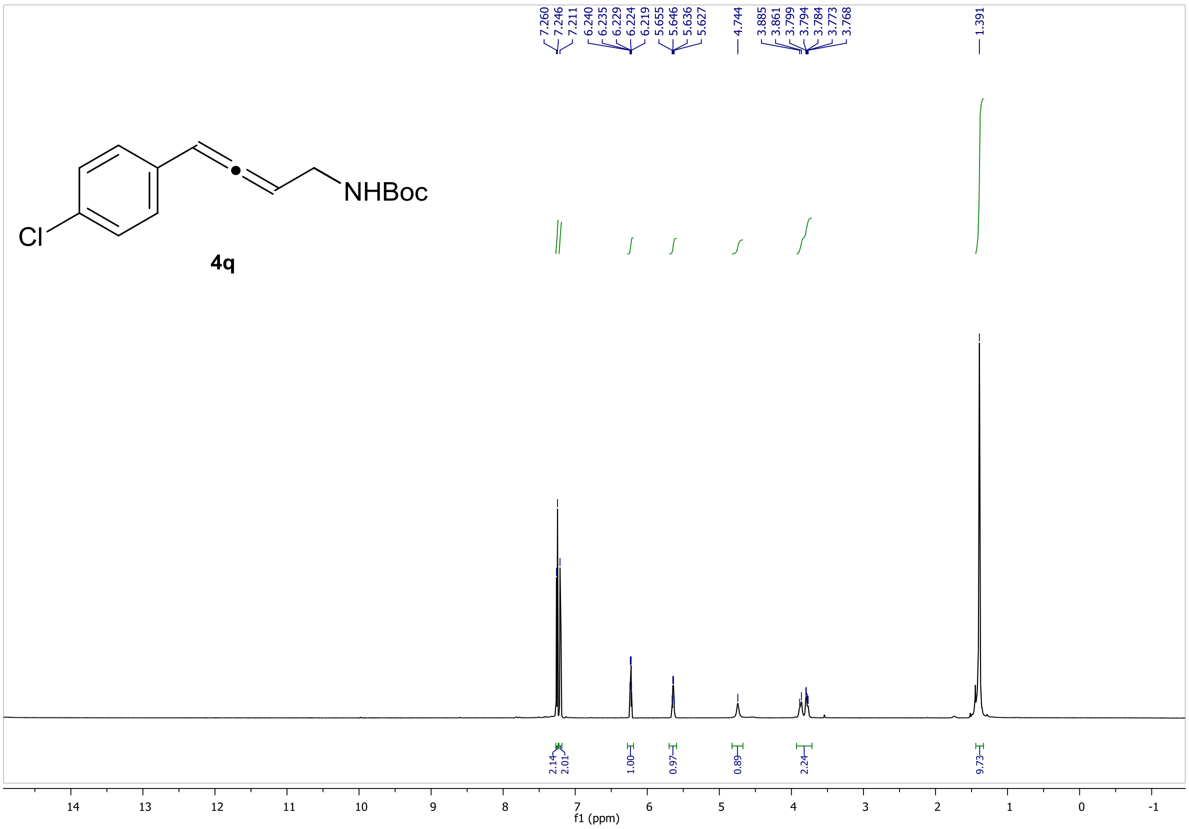

^{13}C NMR, 150 MHz, $CDCl_3$:

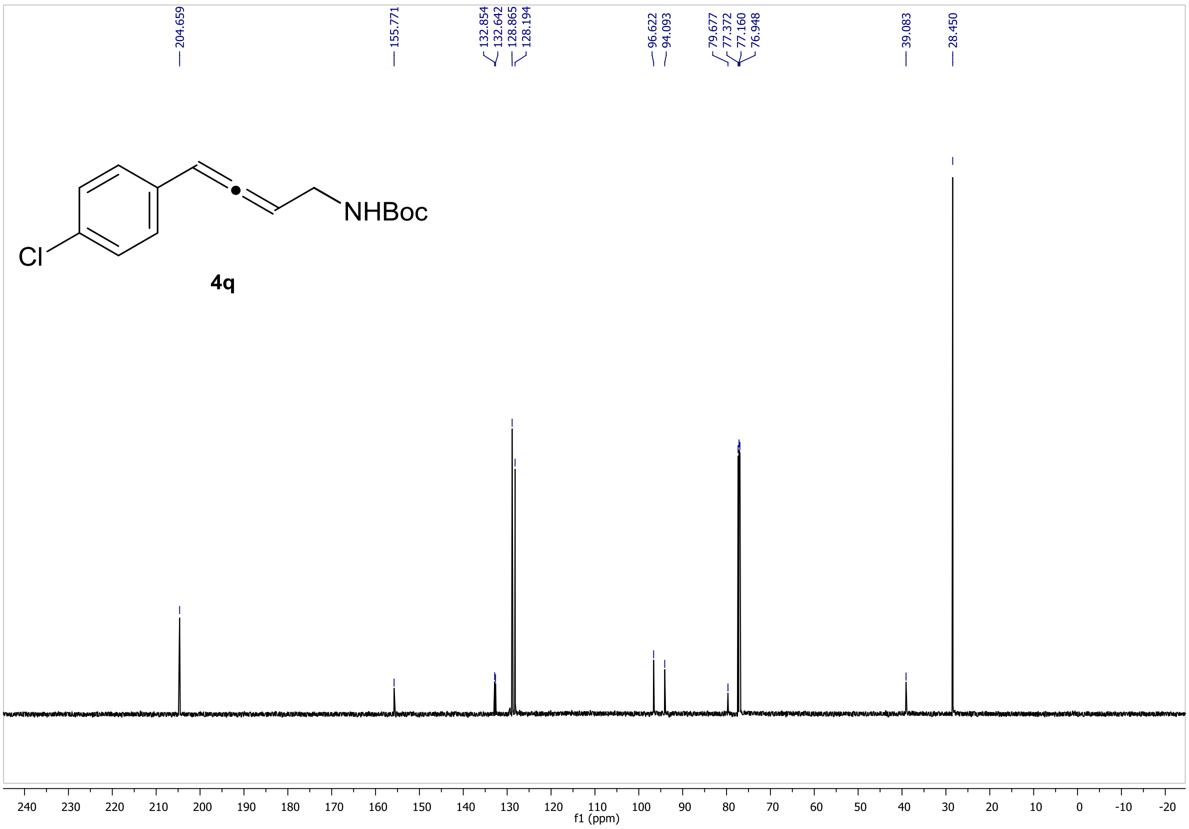

5-(4-chlorophenyl)penta-3,4-dien-1-ol (4r):

^{1}H NMR, 600 MHz, $CDCl_3$:

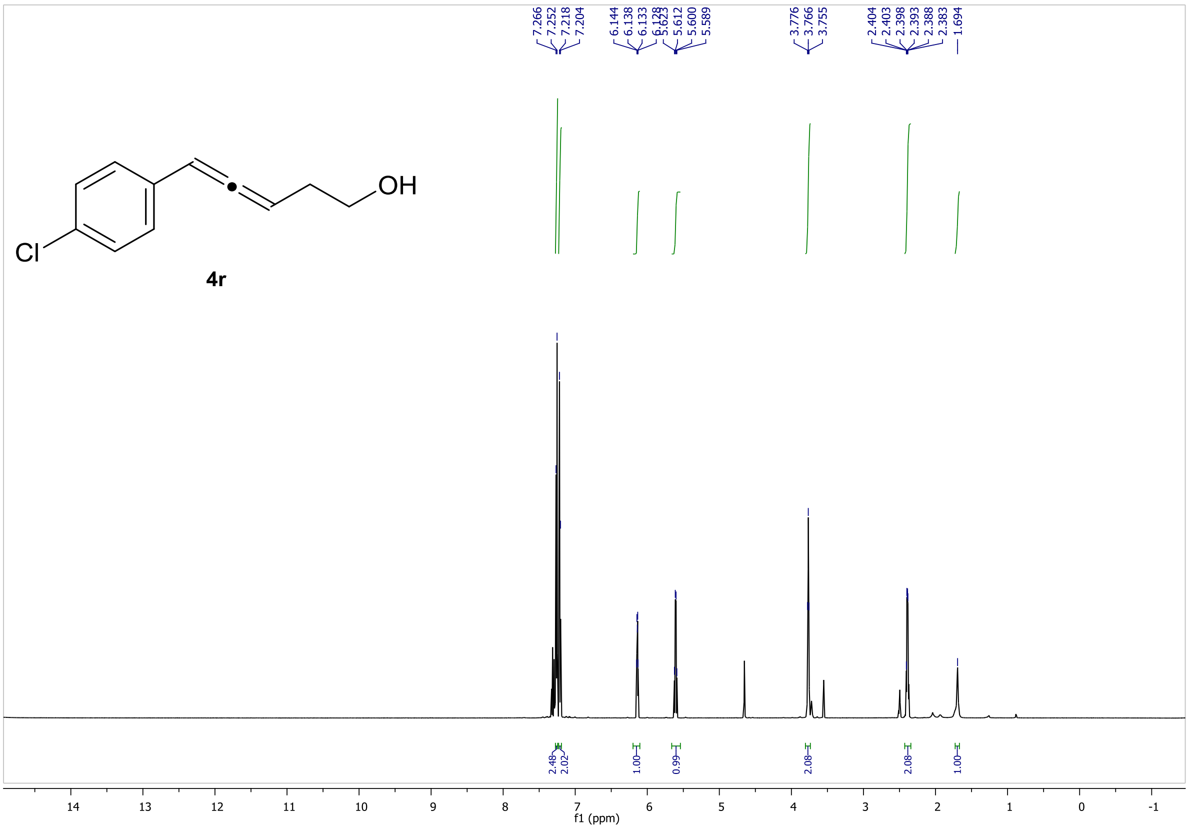

^{13}C NMR, 150 MHz, $CDCl_3$:

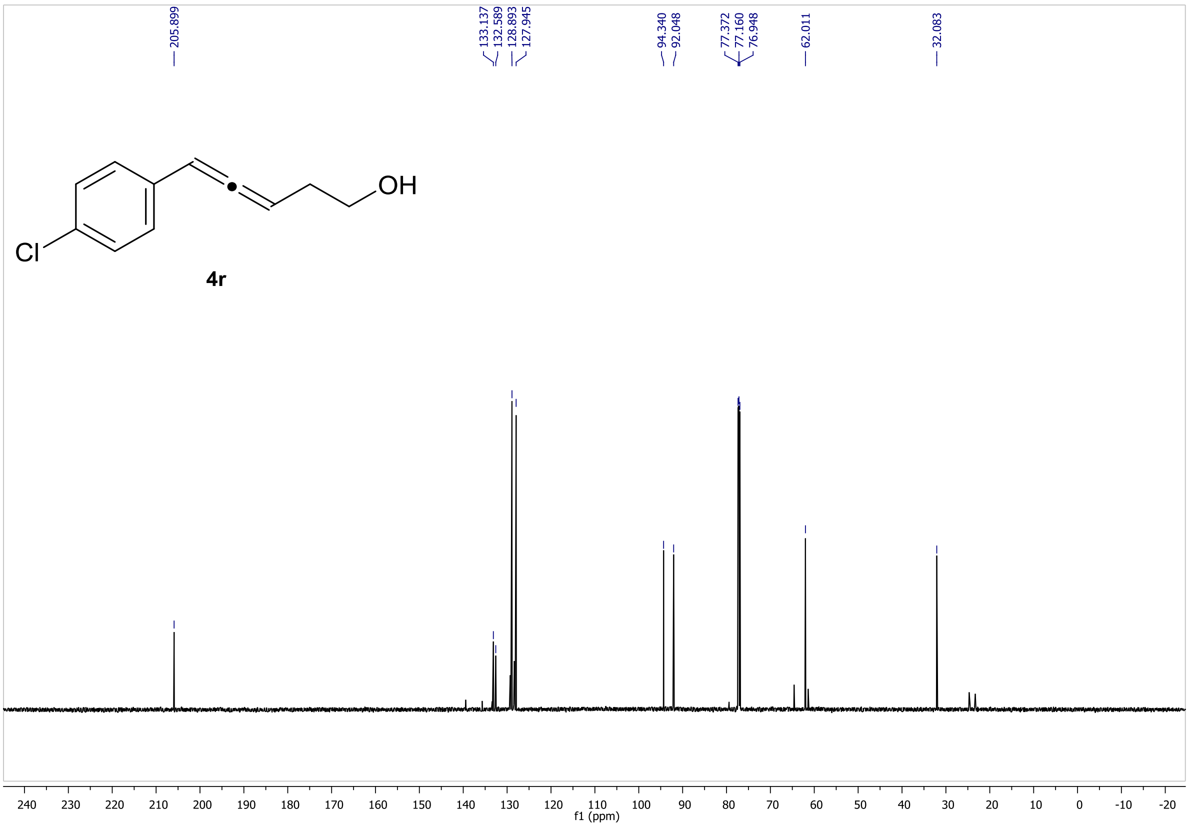

5-(4-chlorophenyl)penta-3,4-dien-2-ol (4s):

^{1}H NMR, 600 MHz, $CDCl_3$:

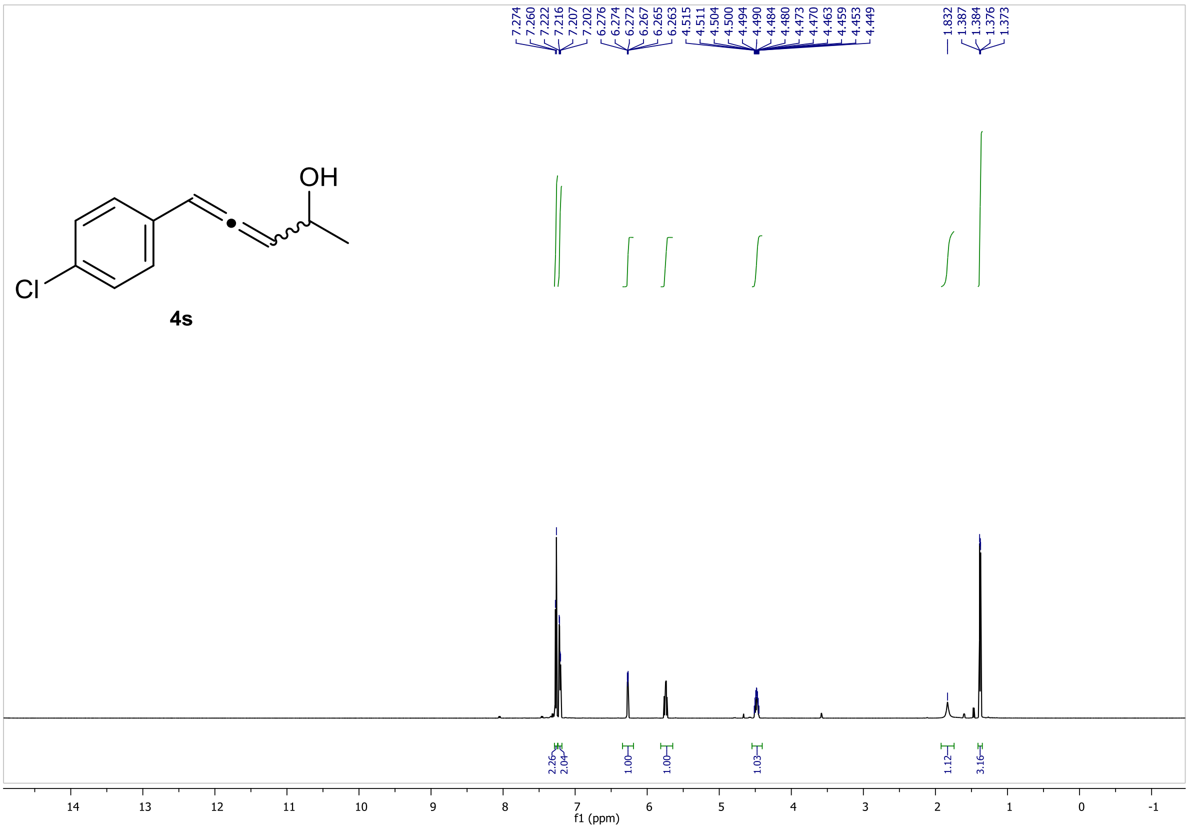

^{13}C NMR, 150 MHz, $CDCl_3$:

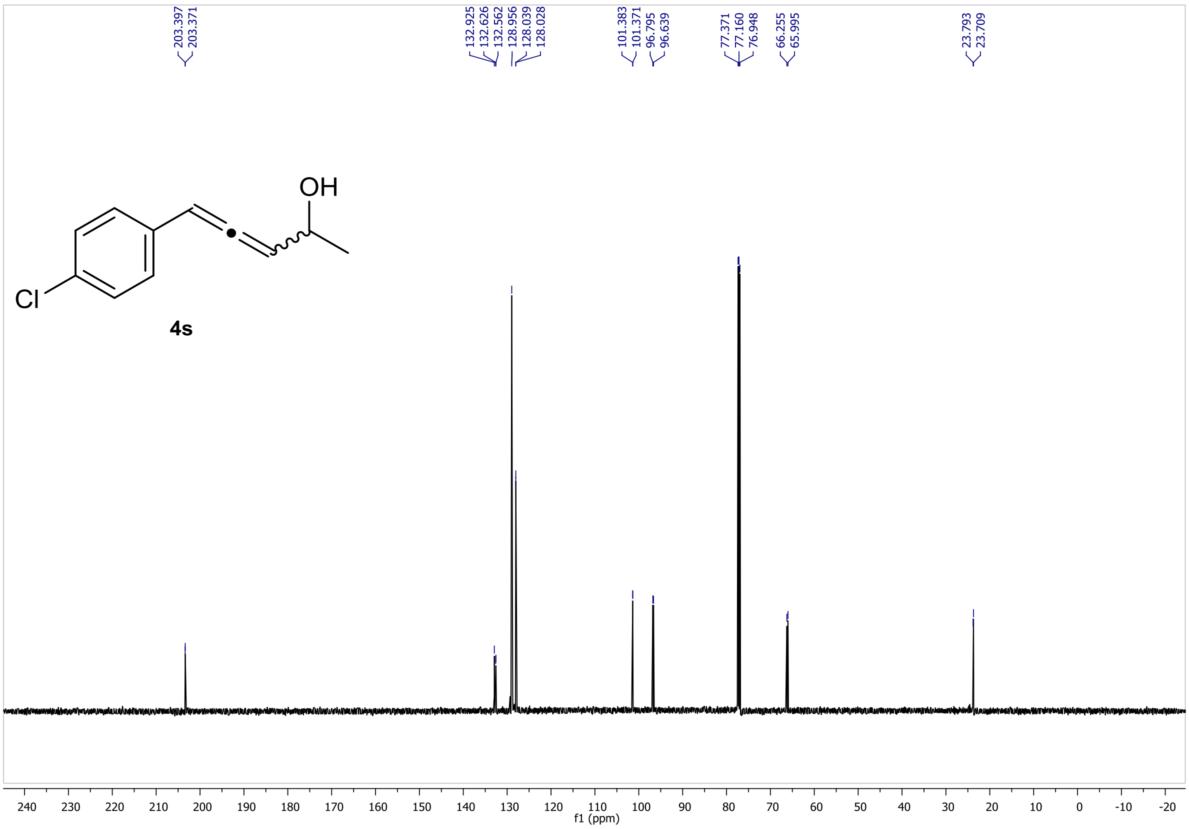

4-(4-chlorophenyl)-1-phenylbuta-2,3-dien-1-ol (4t, *diastereomer A*):

^{1}H NMR, 600 MHz, $CDCl_3$:

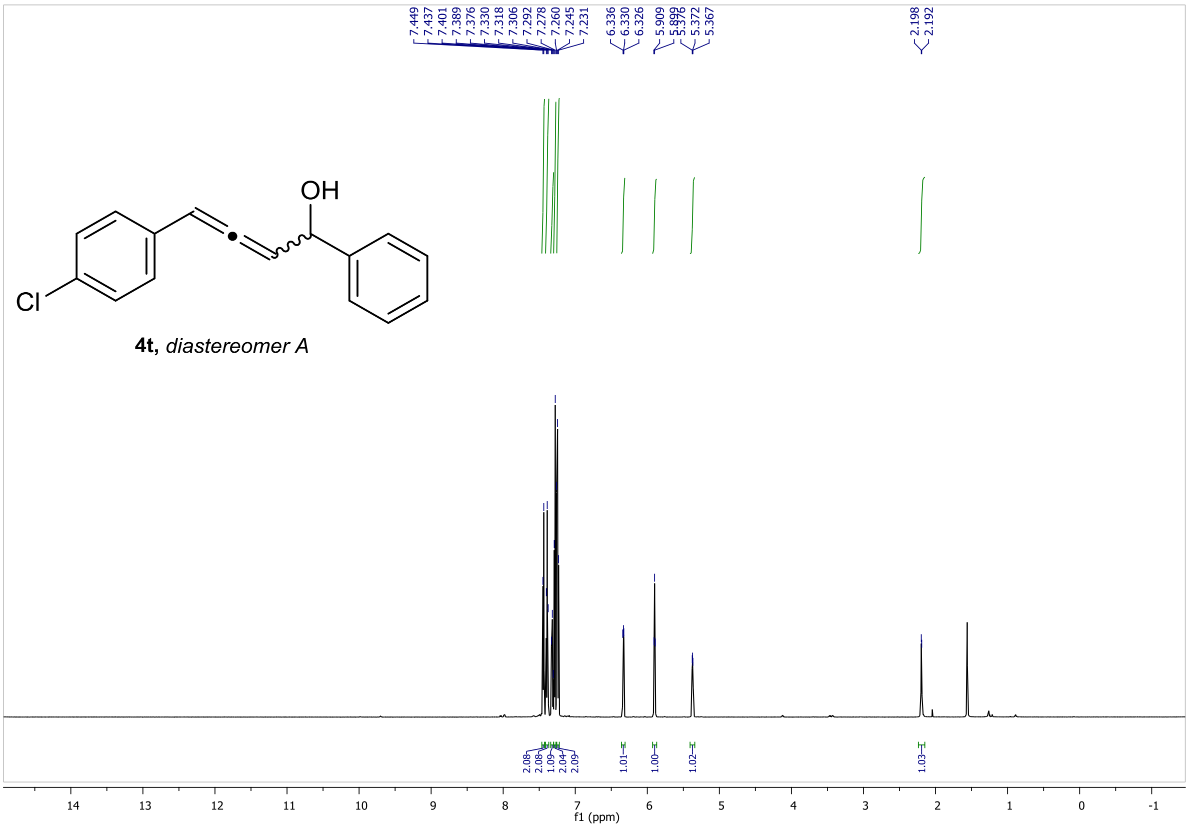

^{13}C NMR, 150 MHz, $CDCl_3$:

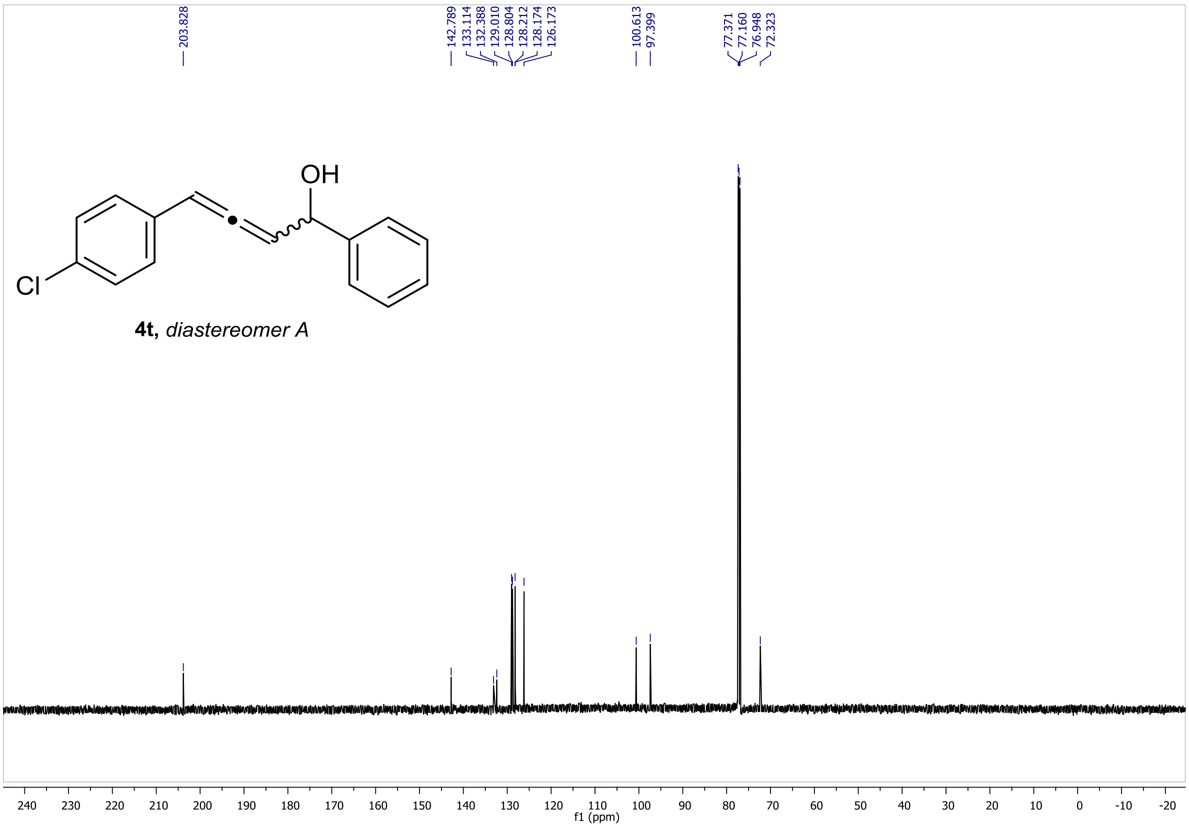

4-(4-chlorophenyl)-1-phenylbuta-2,3-dien-1-ol (4t, *diastereomer B*):

^{1}H NMR, 600 MHz, $CDCl_3$:

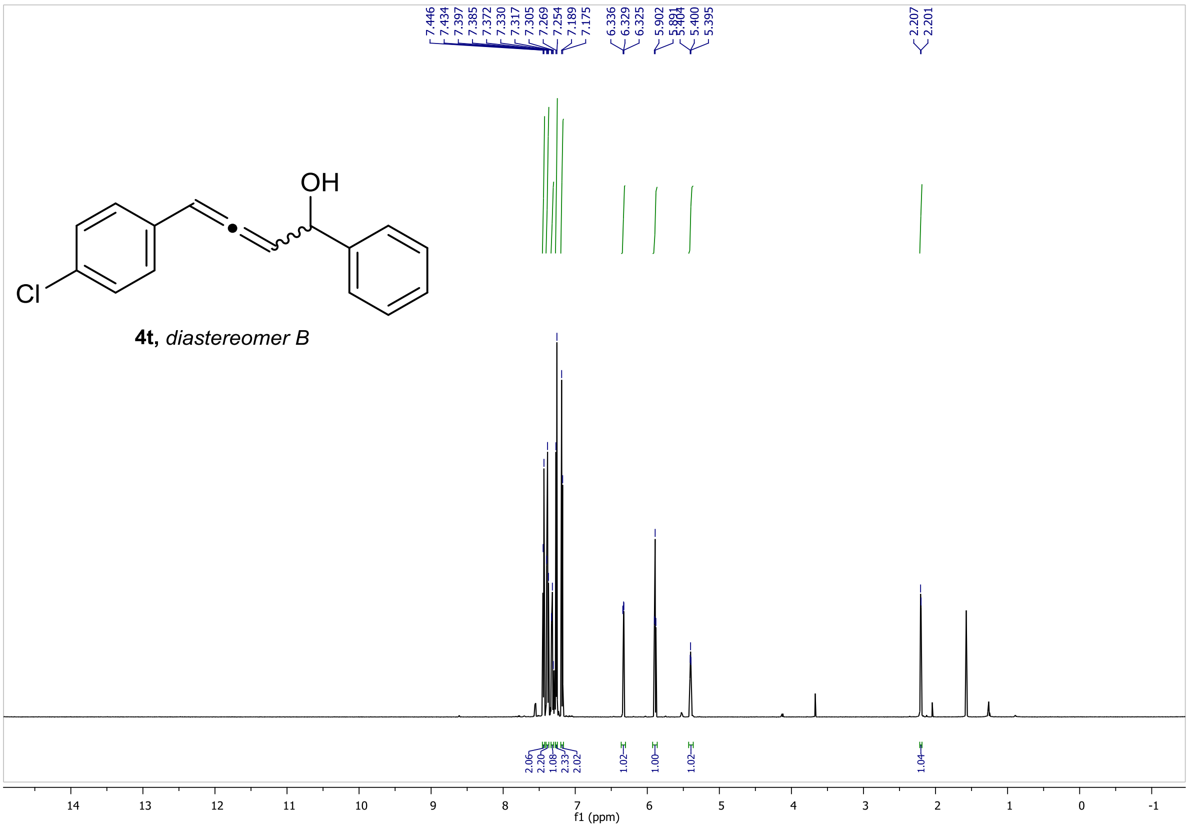

^{13}C NMR, 150 MHz, $CDCl_3$:

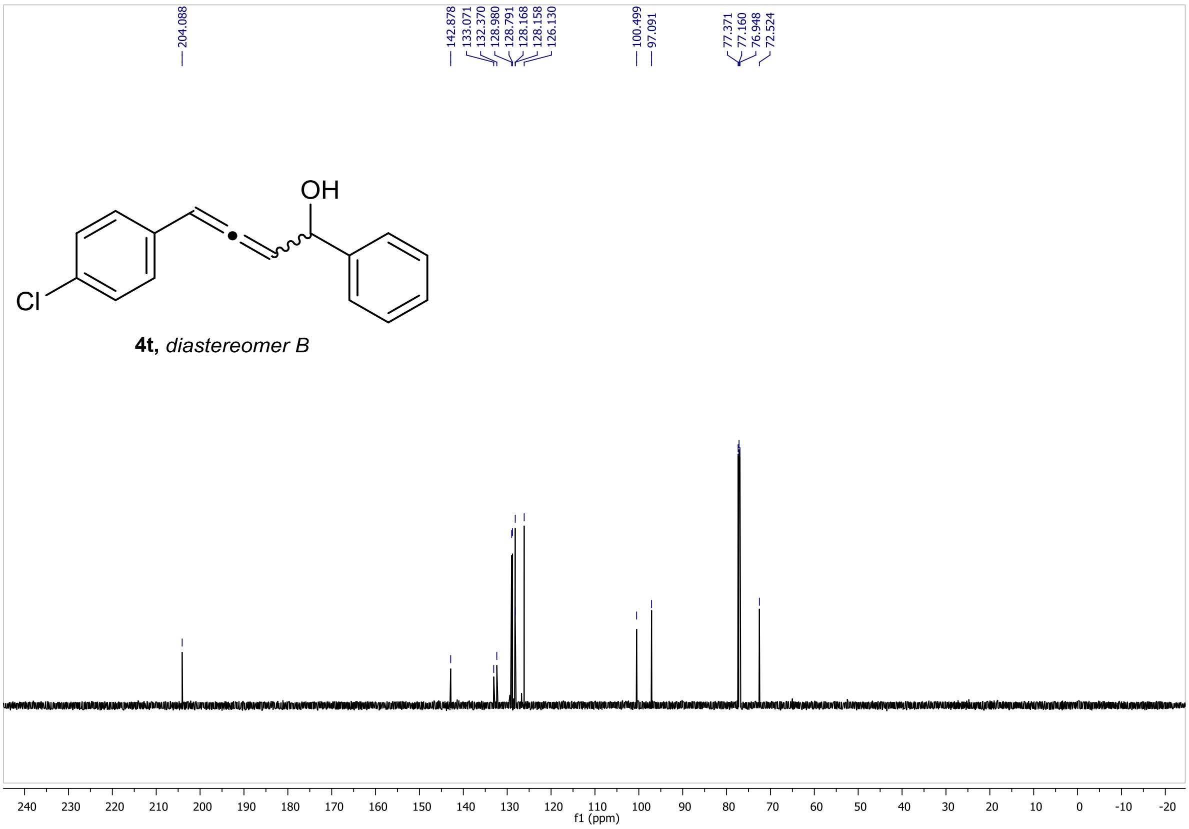

5-(4-chlorophenyl)-2-methylpenta-3,4-dien-2-ol (4u):

^{1}H NMR, 600 MHz, $CDCl_3$:

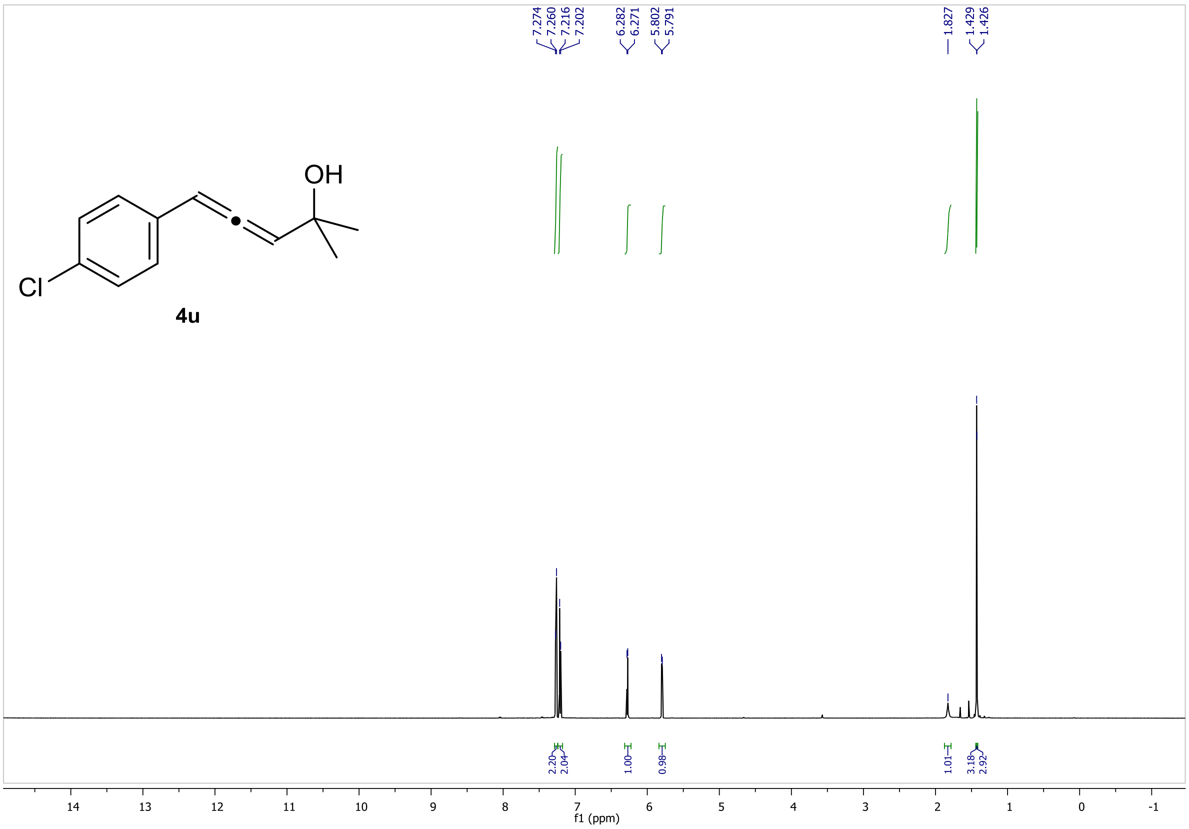

^{13}C NMR, 150 MHz, $CDCl_3$:

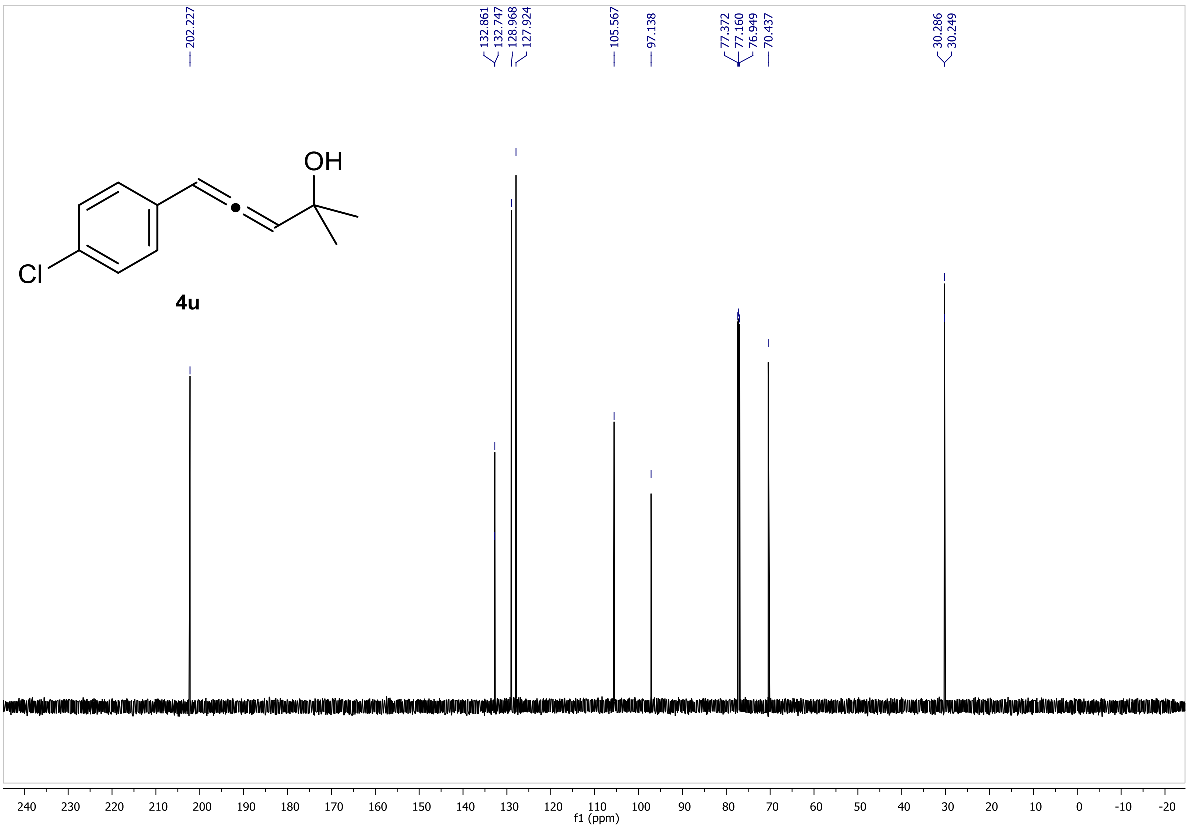

2-(((4-(4-chlorophenyl)buta-2,3-dien-1-yl)oxy)methyl)-3-phenyloxirane (4v):

^{1}H NMR, 600 MHz, $CDCl_3$:

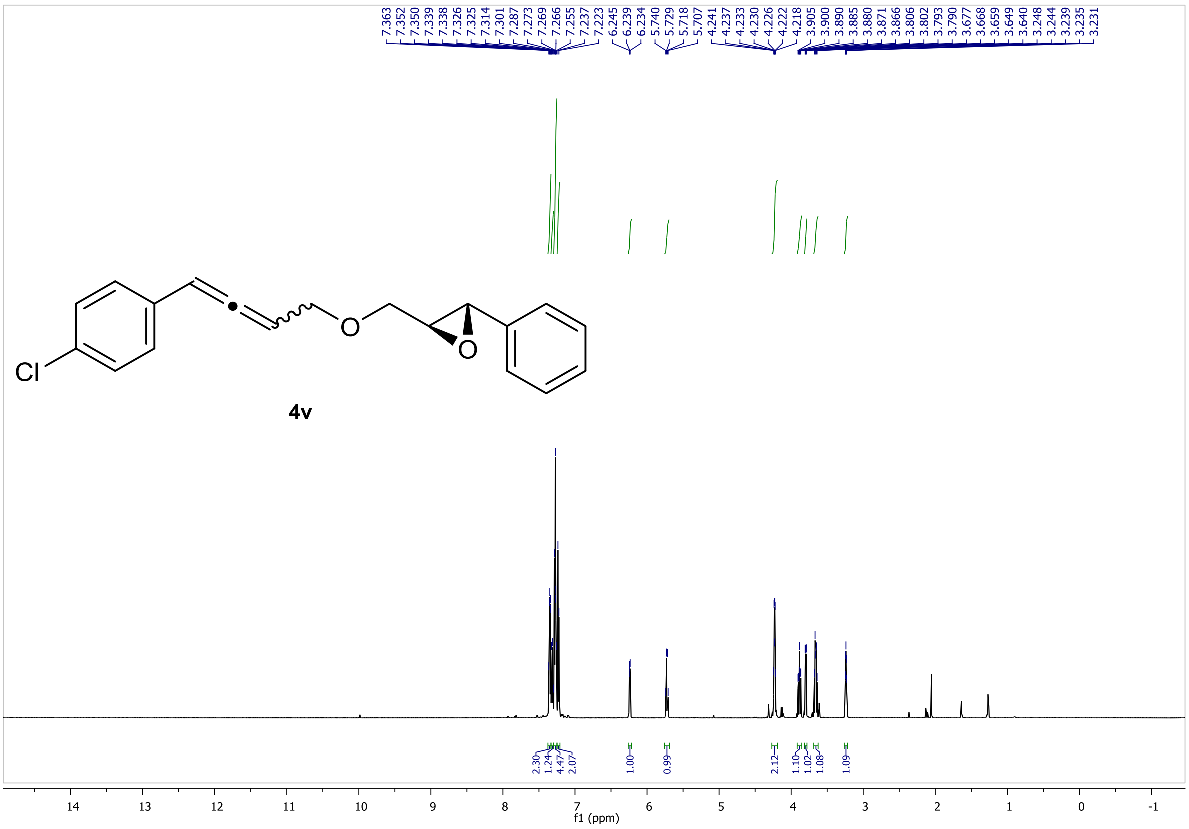

^{13}C NMR, 150 MHz, $CDCl_3$:

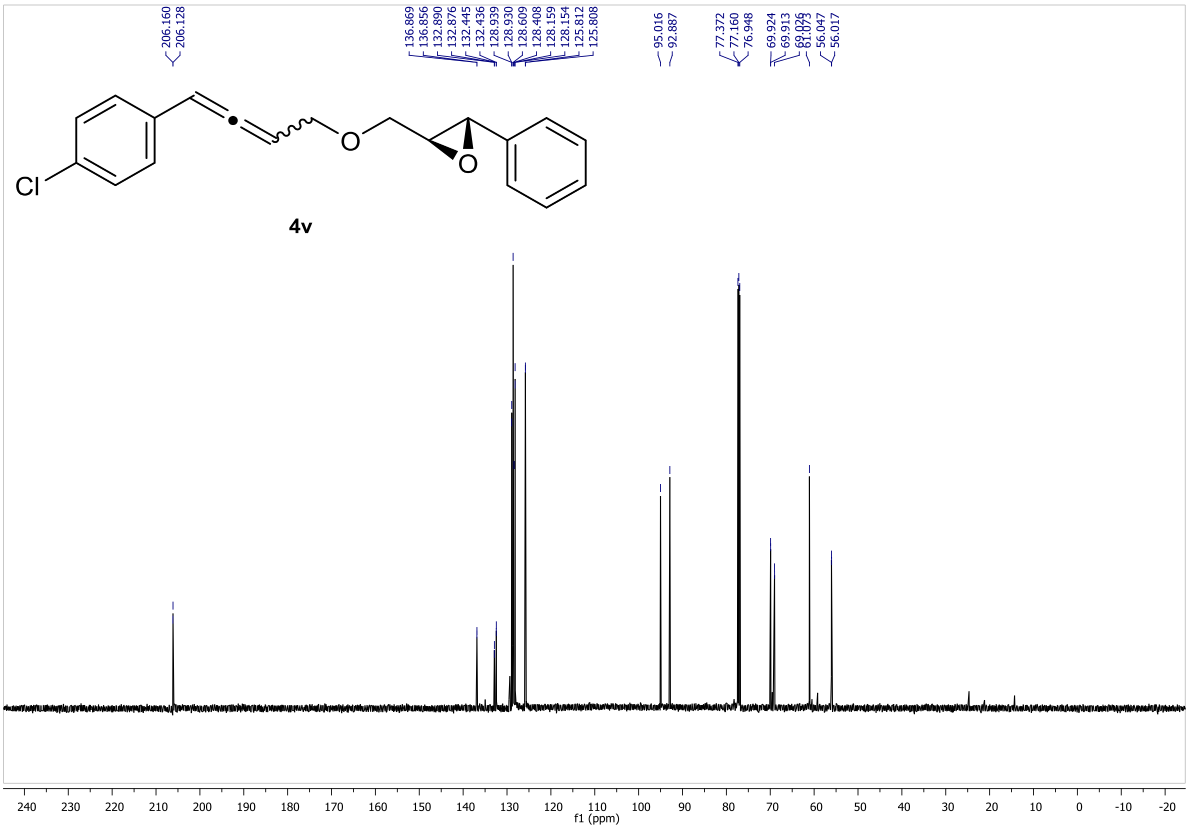

3-(4-chloro)phenylpropa-1,2-dienylferrocene (4w):

^{1}H NMR, 600 MHz, $CDCl_3$:

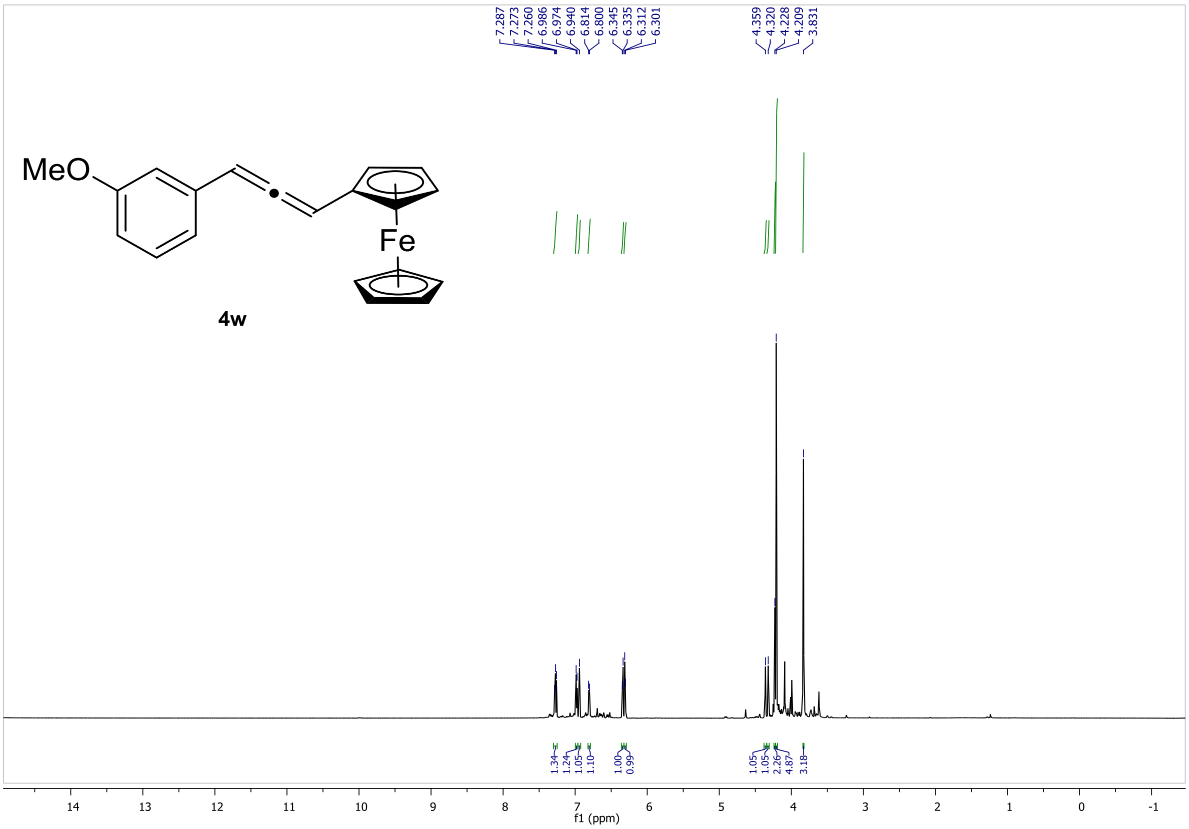

^{13}C NMR, 150 MHz, $CDCl_3$:

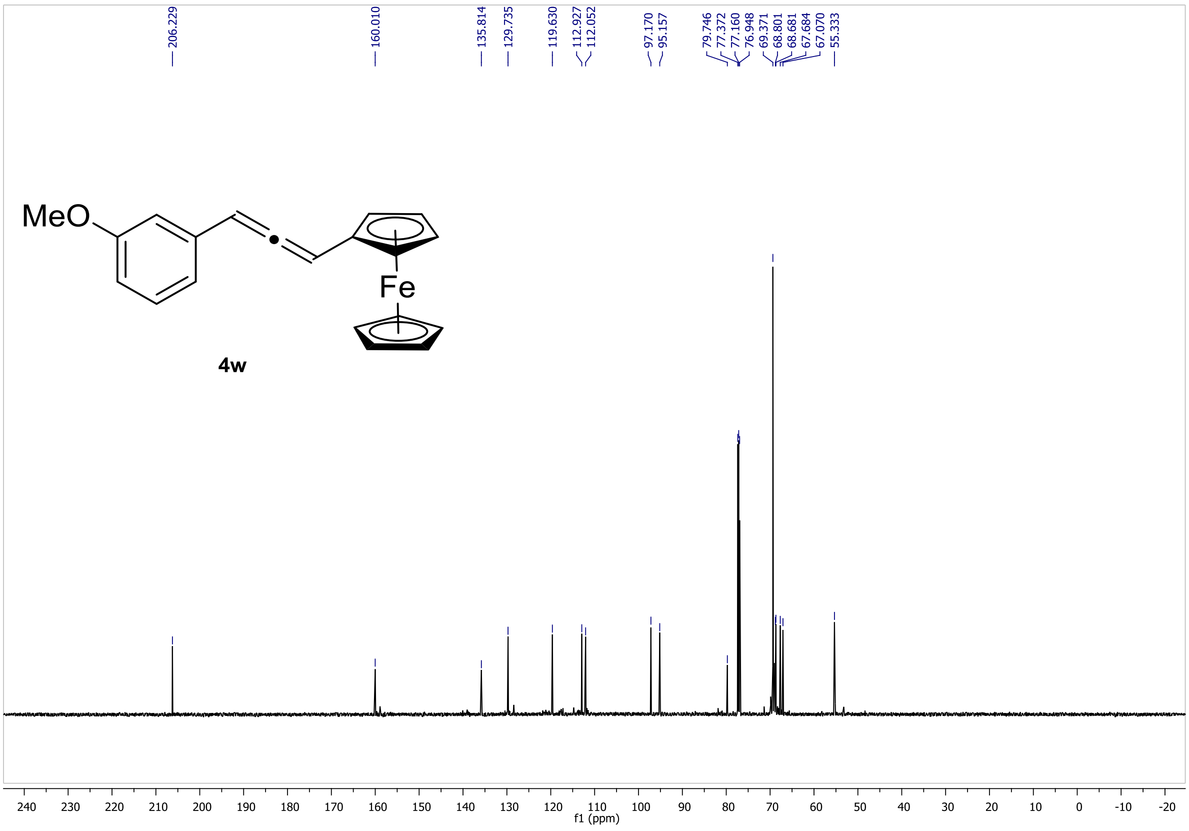

1-chloro-4-(4,4-diethoxybuta-1,2-dien-1-yl)benzene (4x):

^{1}H NMR, 600 MHz, CDCl$_3$:

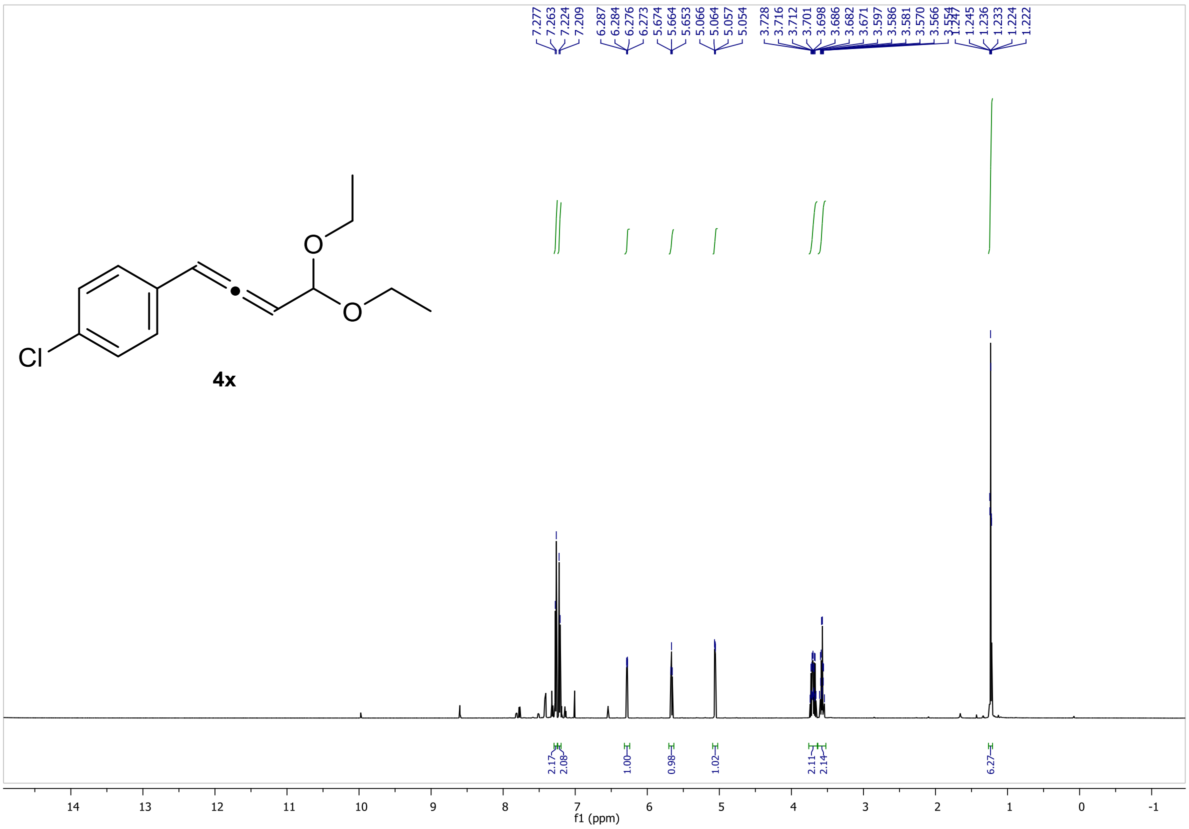

^{13}C NMR, 150 MHz, CDCl$_3$:

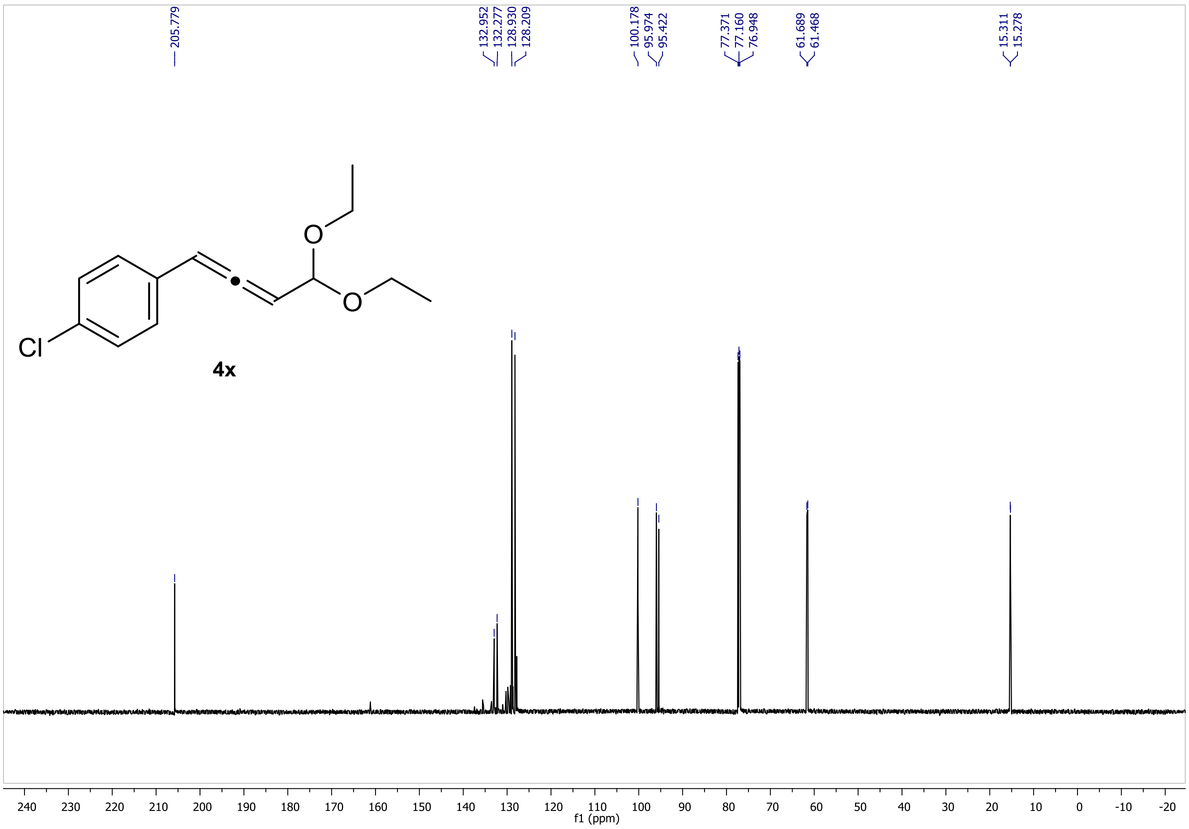

(8R,9S,10R,13S,14S,17R)-17-(3-(4-chlorophenyl)propa-1,2-dien-1-yl)-17-hydroxy-13-methyl-1,2,6,7,8,9,10,11,12,13,14,15,16,17-tetradecahydro-3H-cyclopenta[a]phenanthren-3-one (9, ***diastereomer A*****):**

^{1}H NMR, 600 MHz, $CDCl_3$:

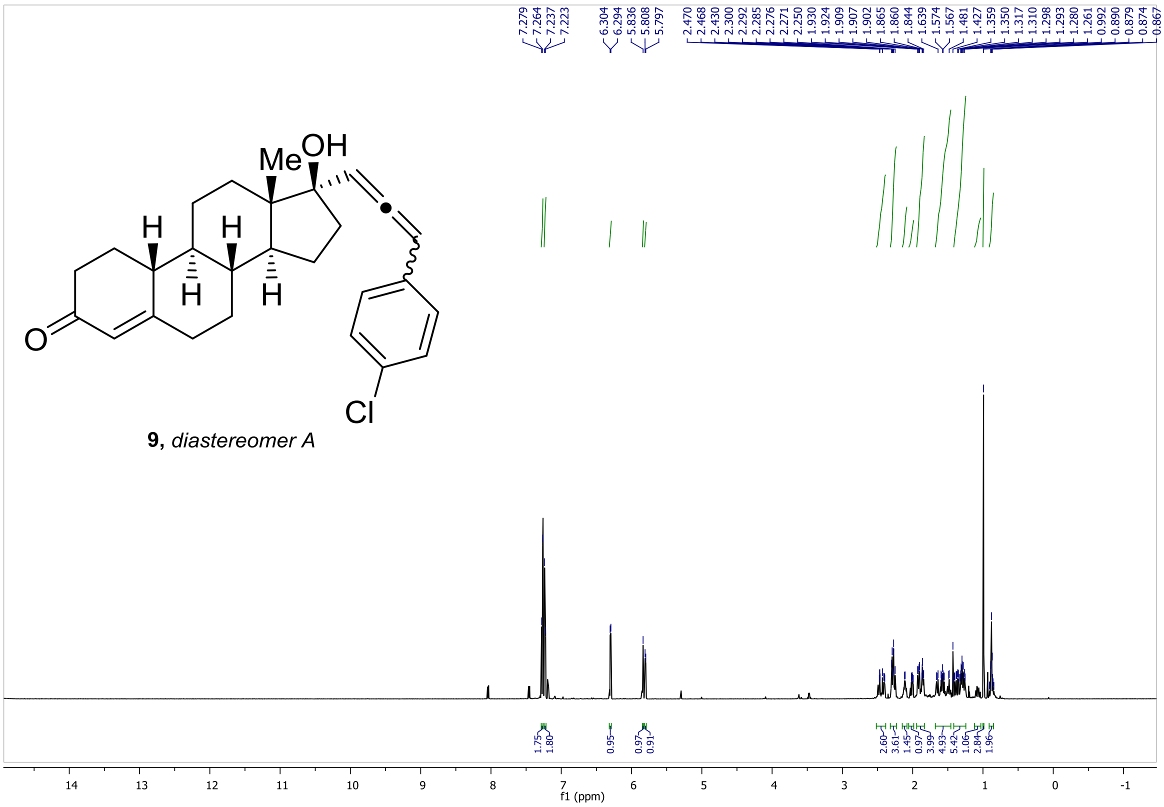

^{13}C NMR, 150 MHz, $CDCl_3$:

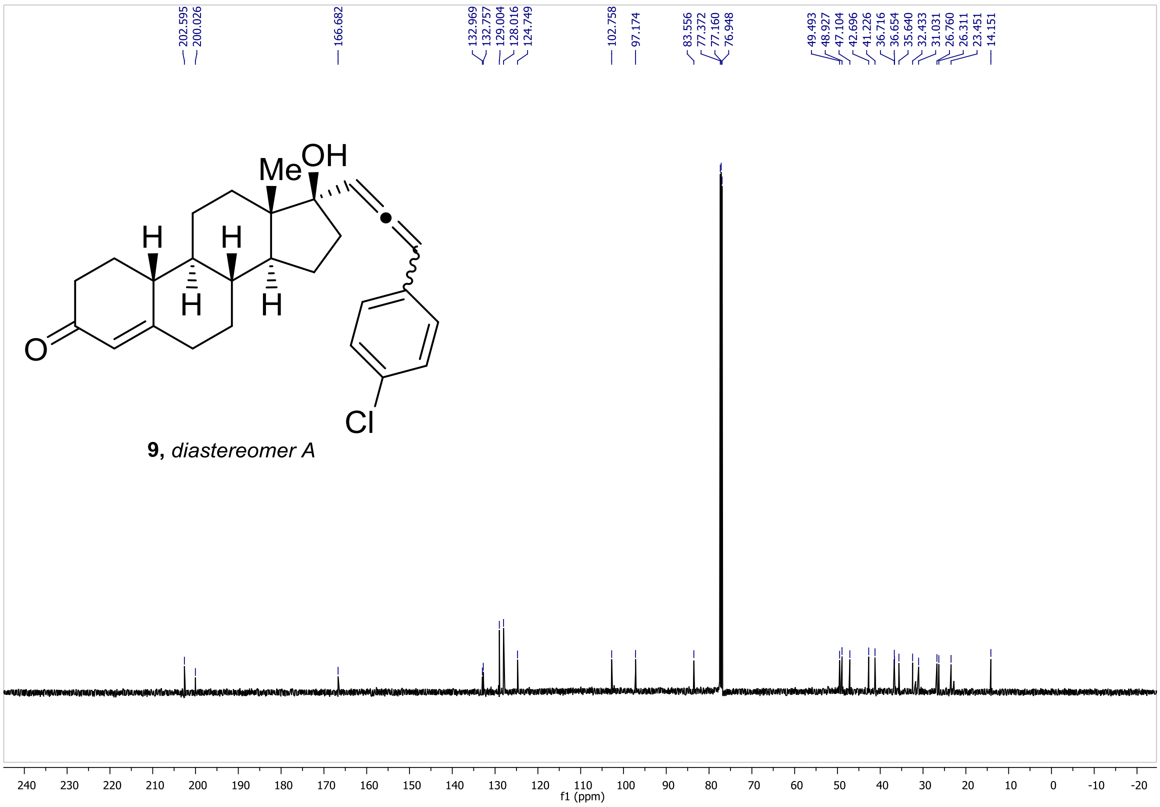

(8R,9S,10R,13S,14S,17R)-17-(3-(4-chlorophenyl)propa-1,2-dien-1-yl)-17-hydroxy-13-methyl-1,2,6,7,8,9,10,11,12,13,14,15,16,17-tetradecahydro-3H-cyclopenta[a]phenanthren-3-one (9, ***diastereomer B*****):**

^{1}H NMR, 600 MHz, $CDCl_3$:

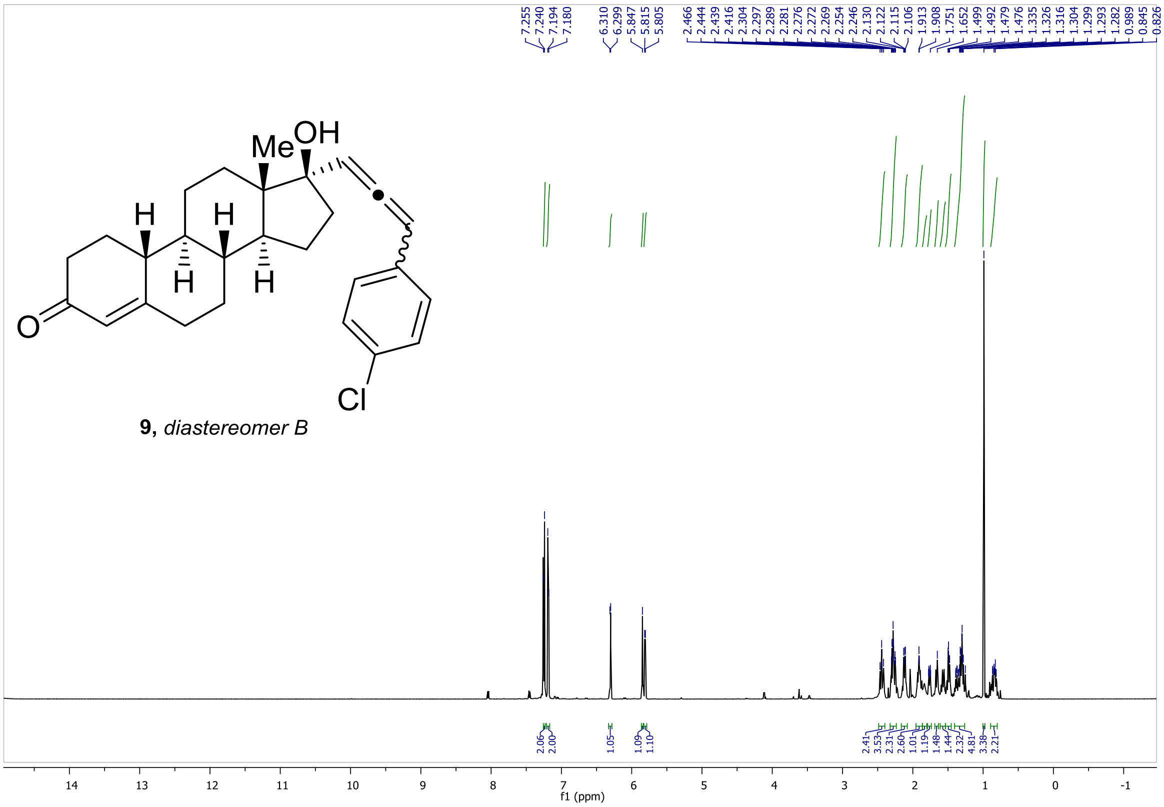

^{13}C NMR, 150 MHz, $CDCl_3$:

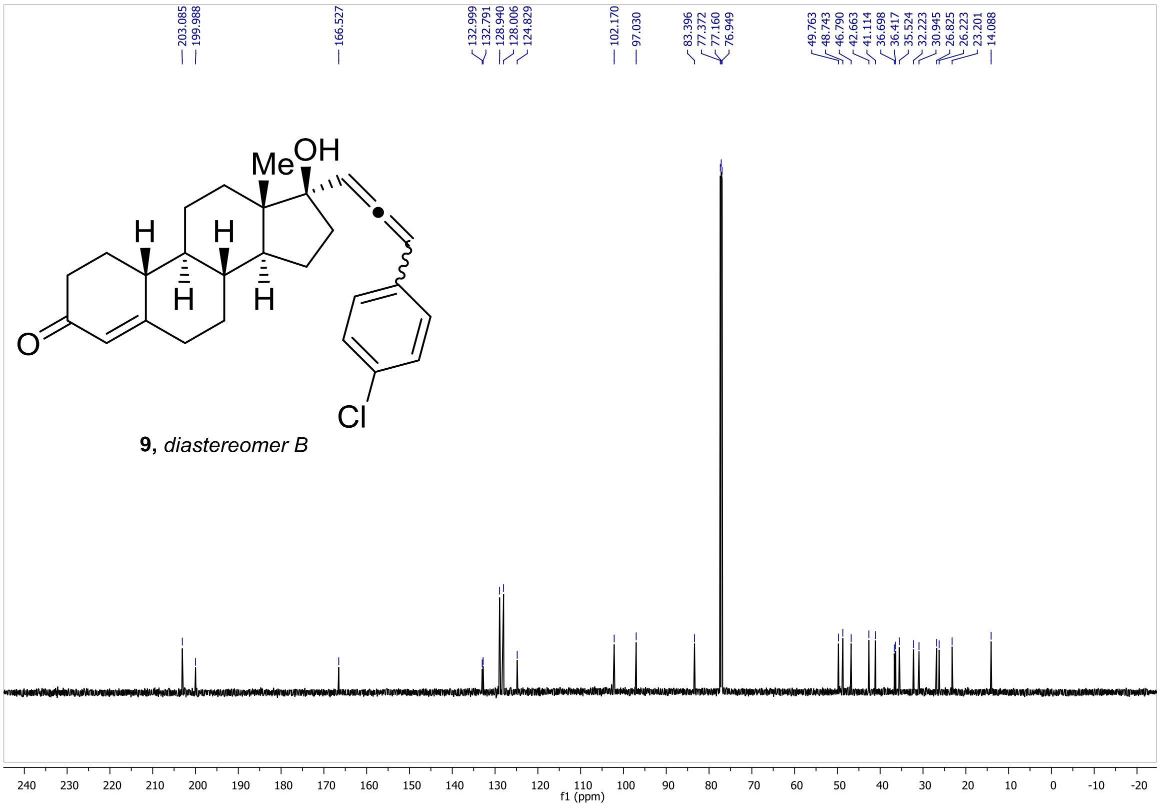

4-(4-((1*R*)-(6-methoxyquinolin-4-yl)((2*S*)-5-vinylquinuclidin-2-yl)methoxy)buta-1,2-dien-1-yl)benzonitrile (11):

^{1}H NMR, 600 MHz, $CDCl_3$:

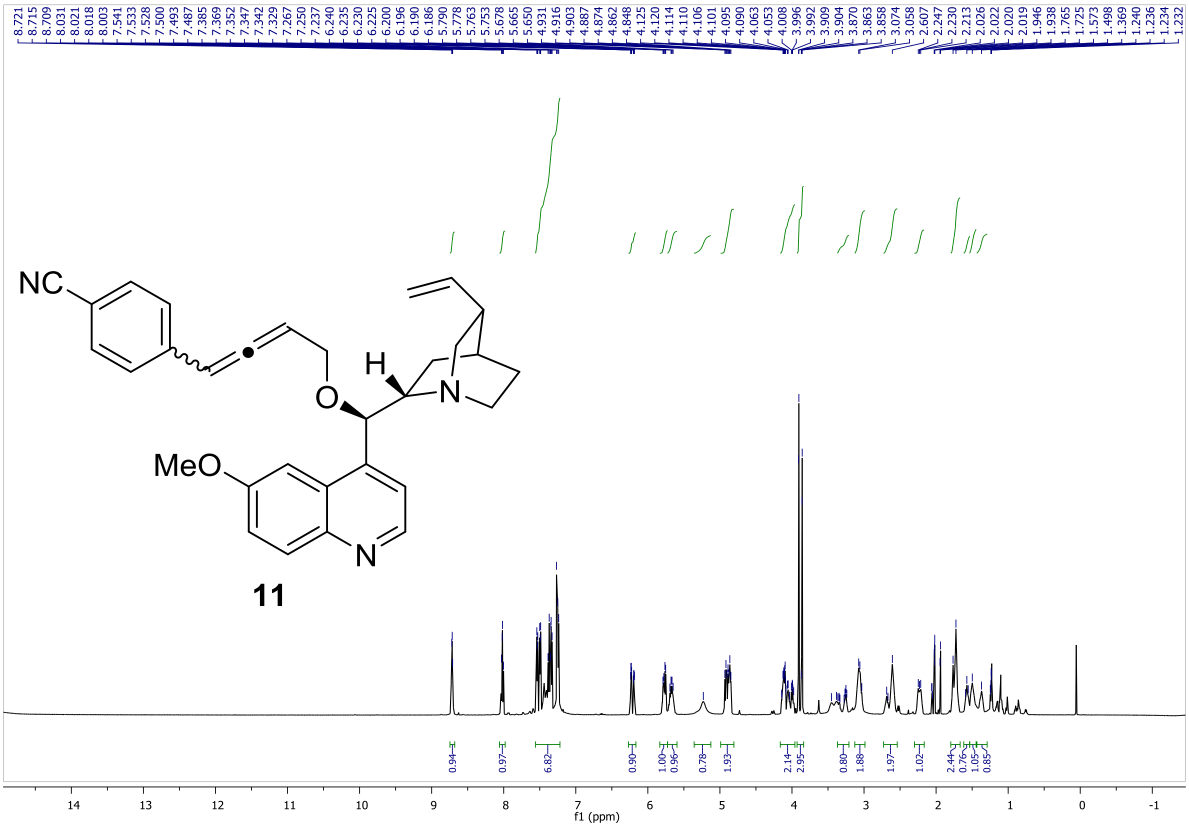

^{13}C NMR, 150 MHz, $CDCl_3$:

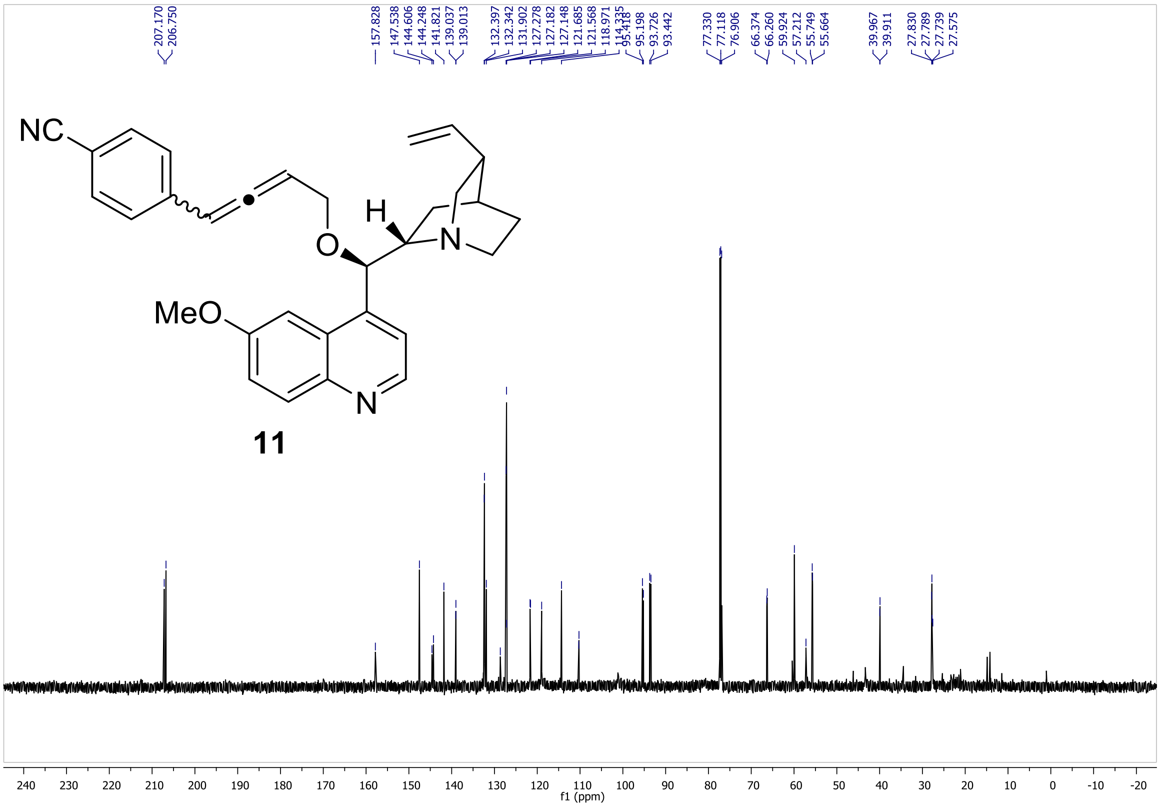

4-methyl-*N*-(4-phenylpenta-2,3-dien-1-yl)benzenesulfonamide (12a):

^{1}H NMR, 600 MHz, $CDCl_3$:

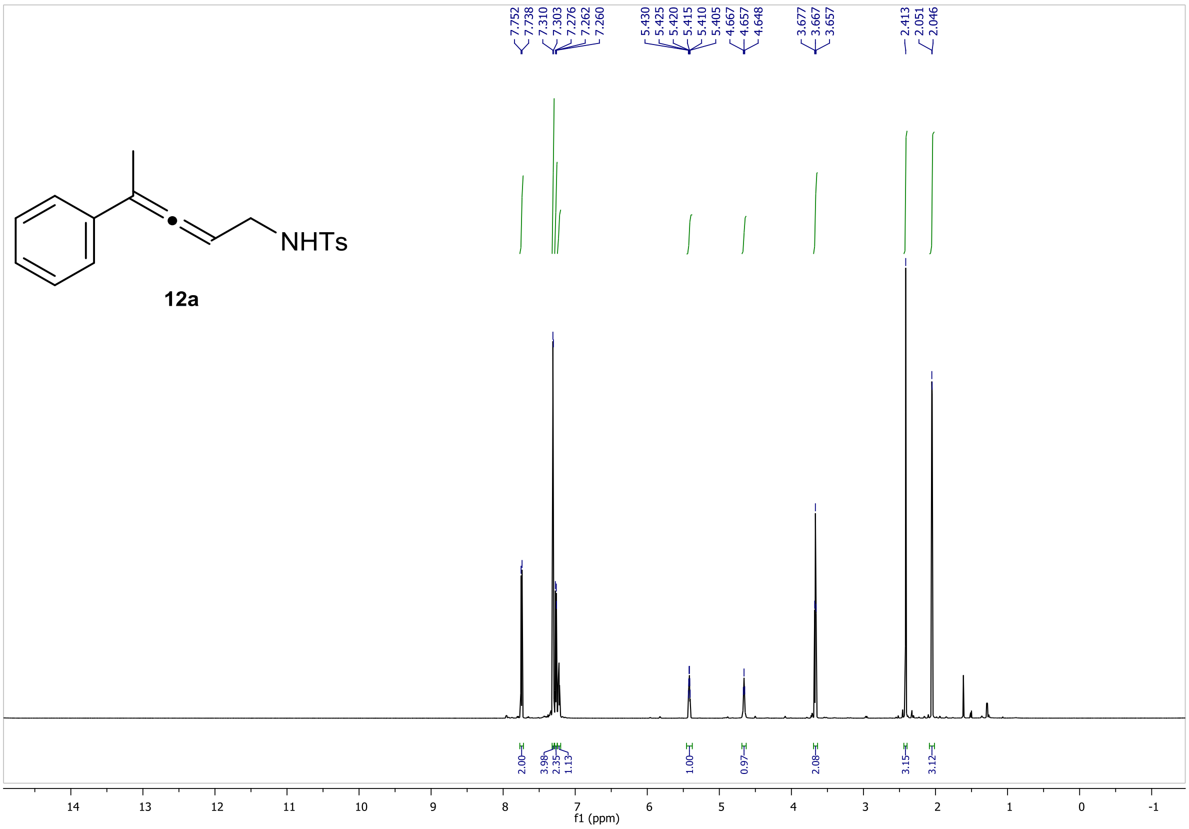

^{13}C NMR, 150 MHz, $CDCl_3$:

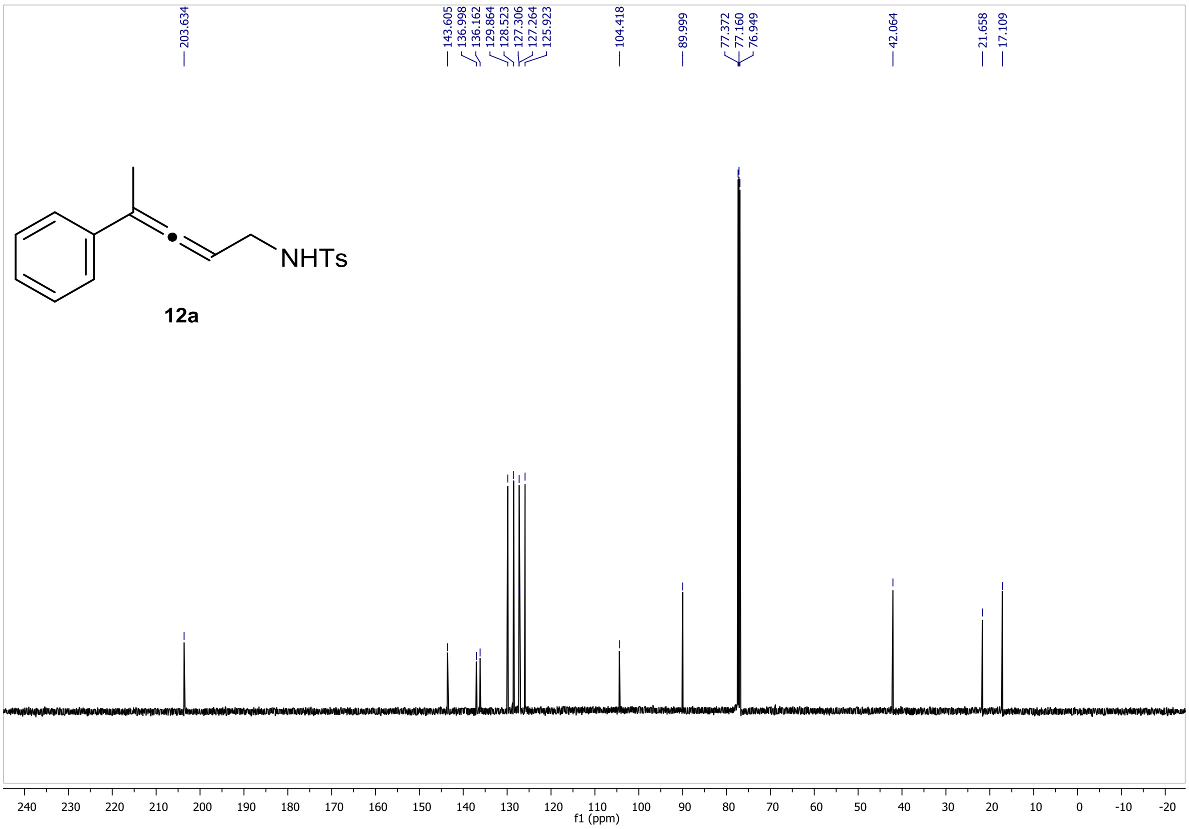

***tert*-butyl (4-phenylpenta-2,3-dien-1-yl)carbamate (12b):**

^{1}H NMR, 600 MHz, $CDCl_3$:

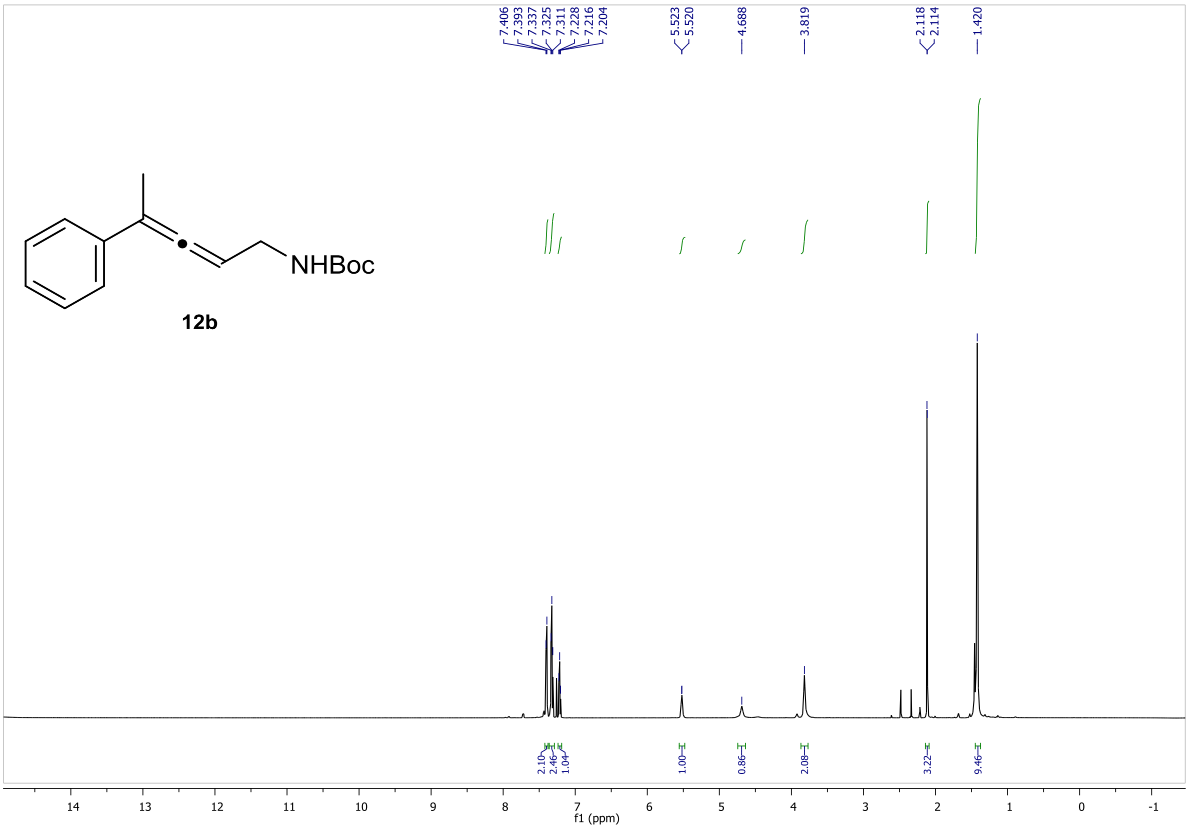

^{13}C NMR, 150 MHz, $CDCl_3$:

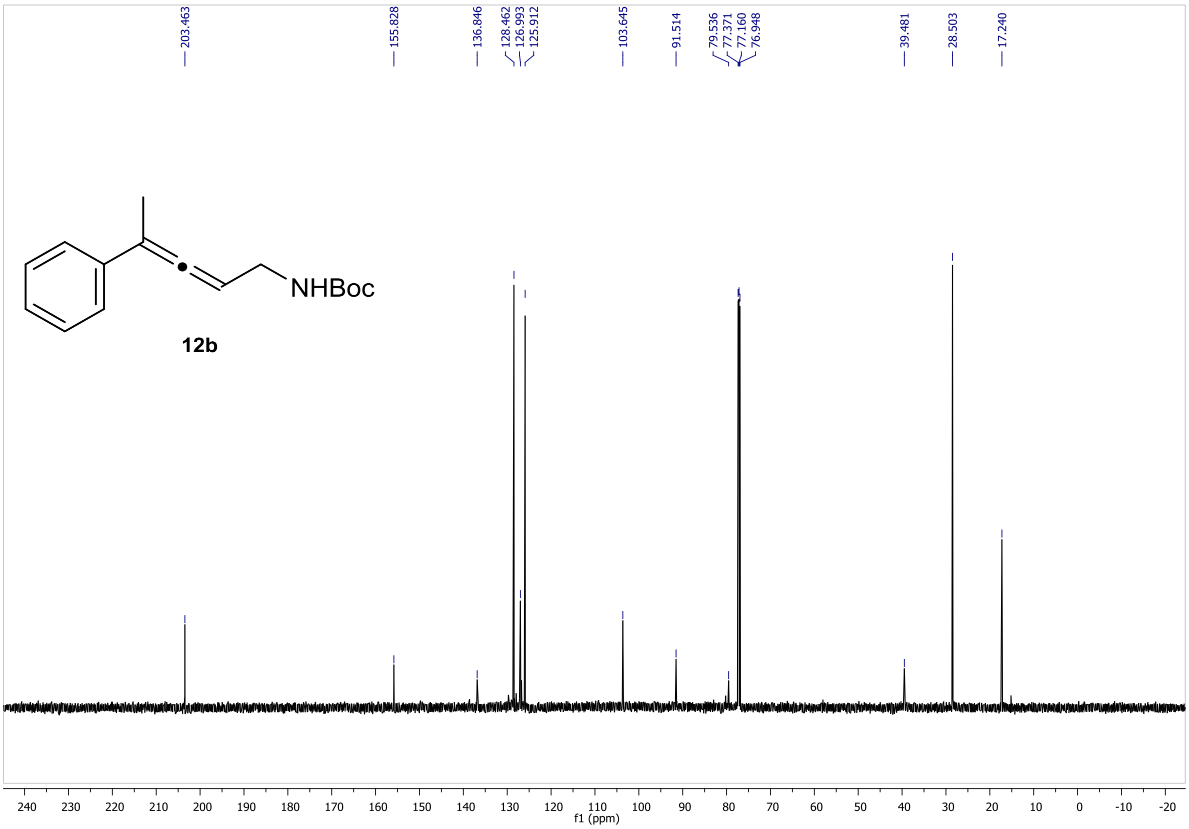

(5-methoxypenta-2,3-dien-2-yl)benzene (12c):

^{1}H NMR, 600 MHz, $CDCl_3$:

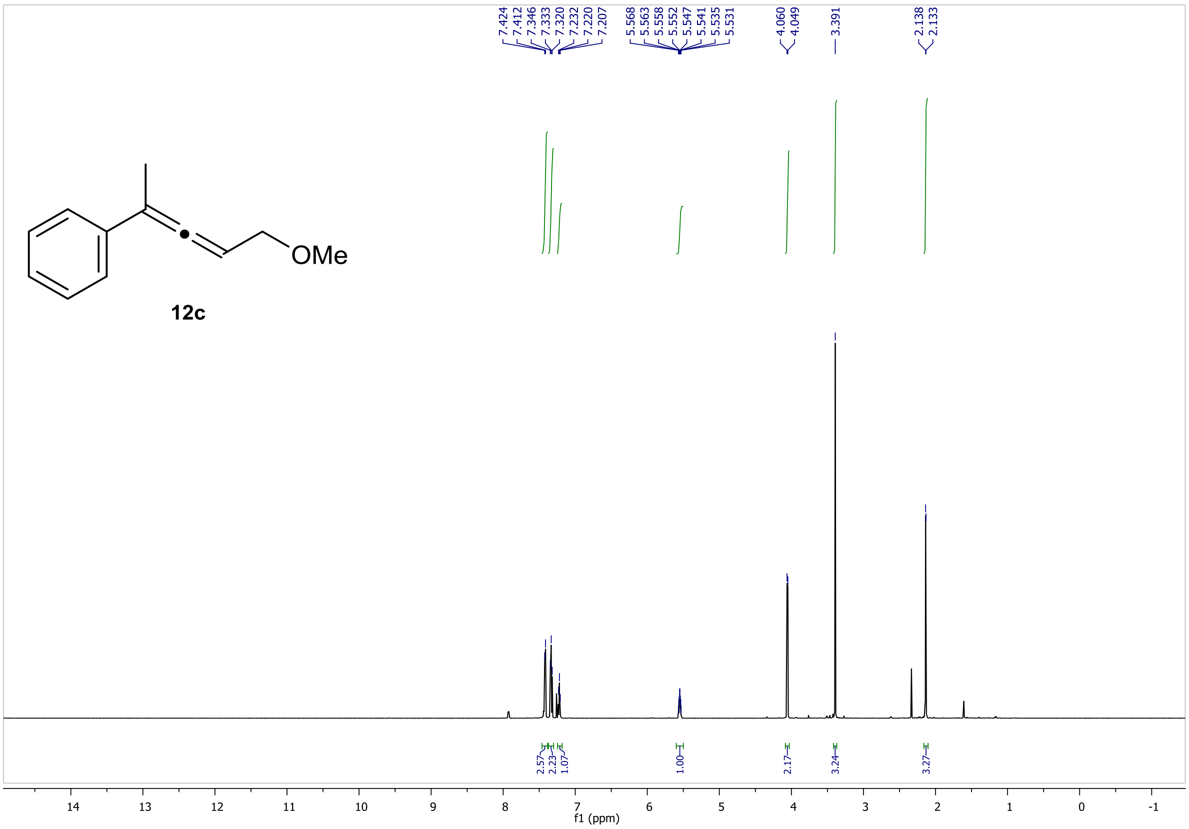

^{13}C NMR, 150 MHz, $CDCl_3$:

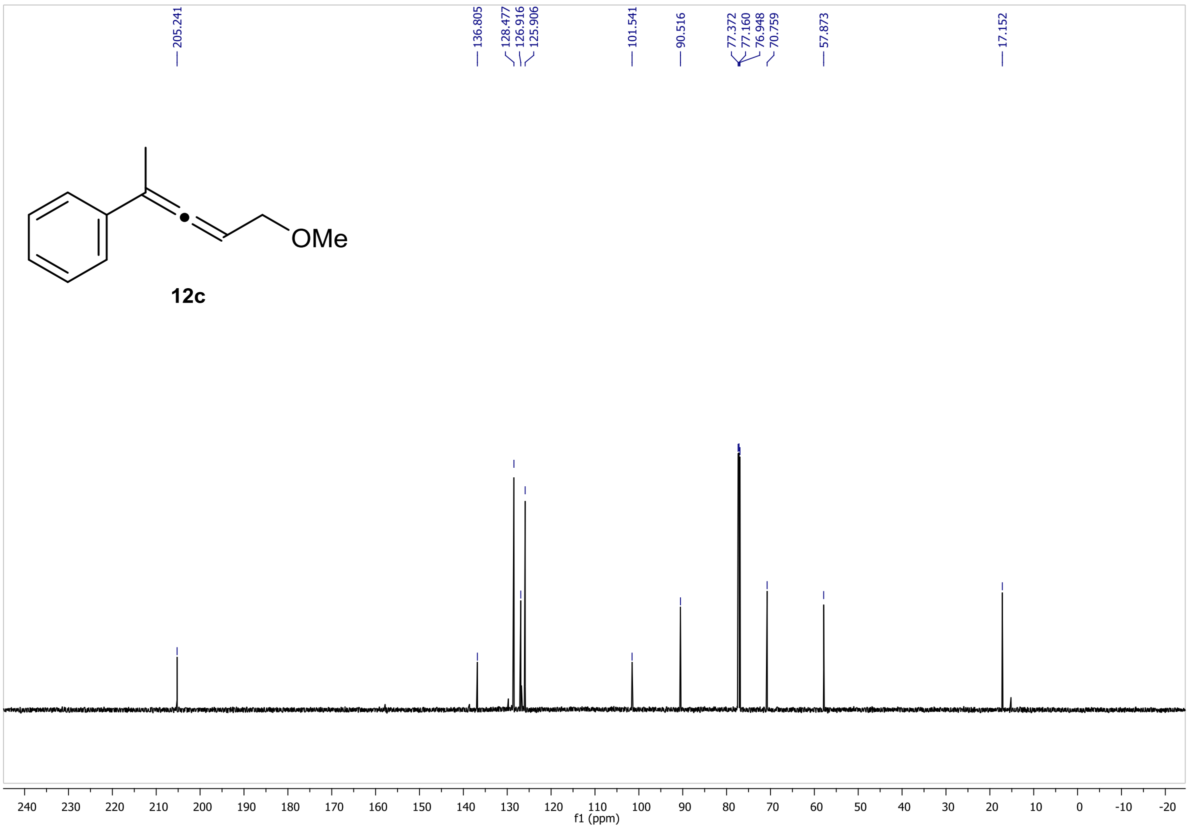

7-phenylocta-5,6-dienenitrile (12d):

^{1}H NMR, 600 MHz, $CDCl_3$:

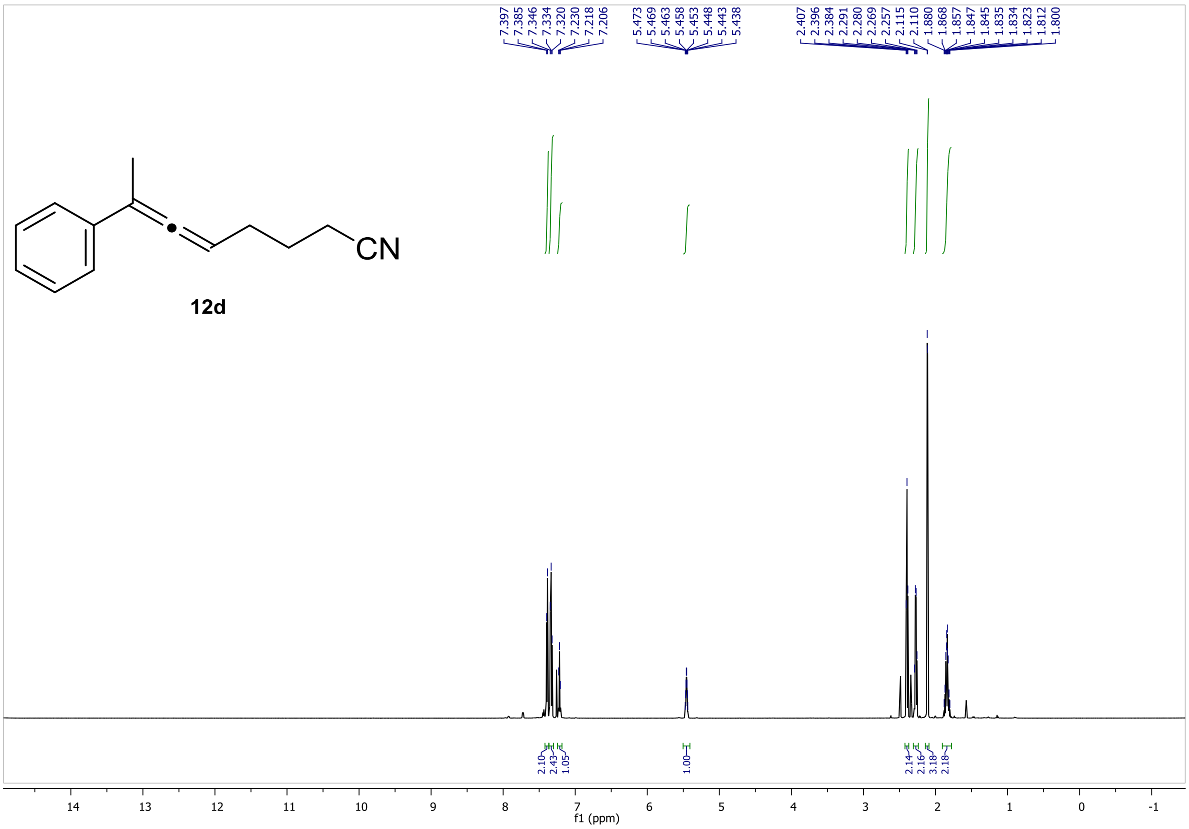

^{13}C NMR, 150 MHz, $CDCl_3$:

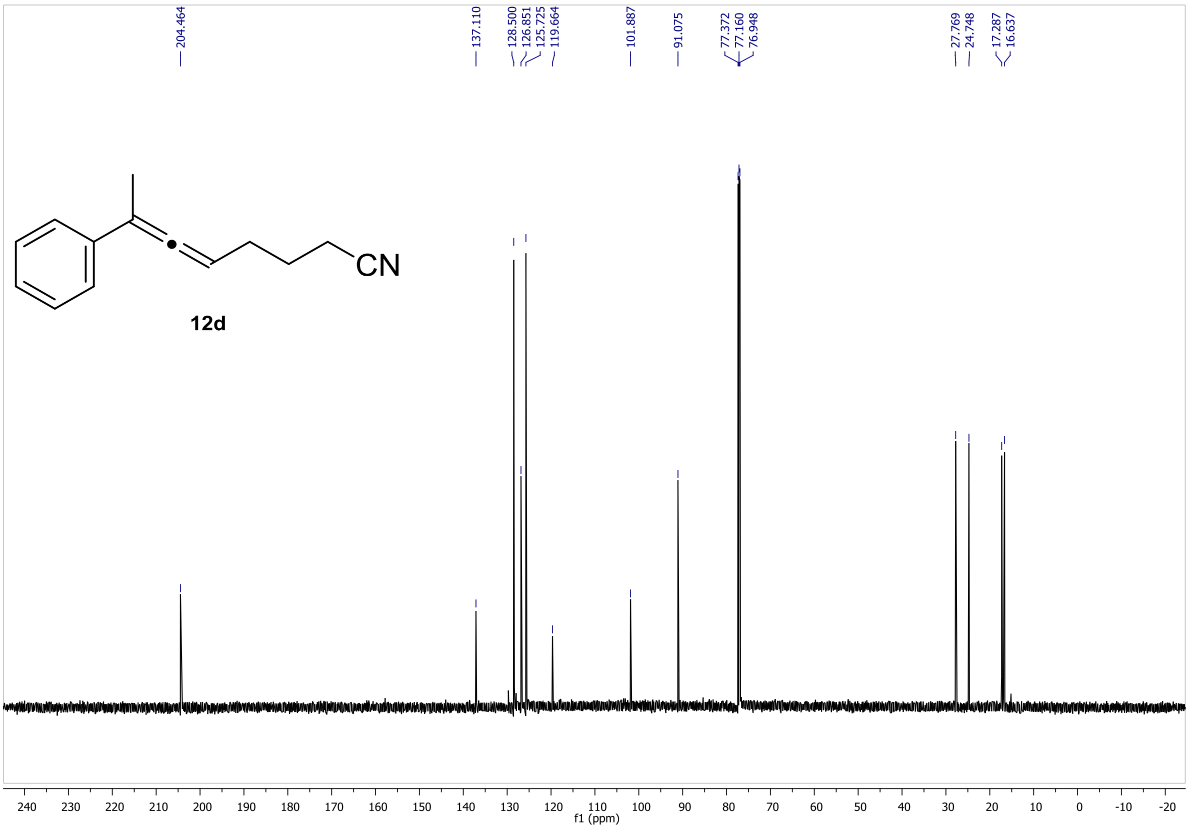

***tert*-butyl (4-(4-chlorophenyl)penta-2,3-dien-1-yl)carbamate (12e):**

^{1}H NMR, 600 MHz, CDCl$_3$:

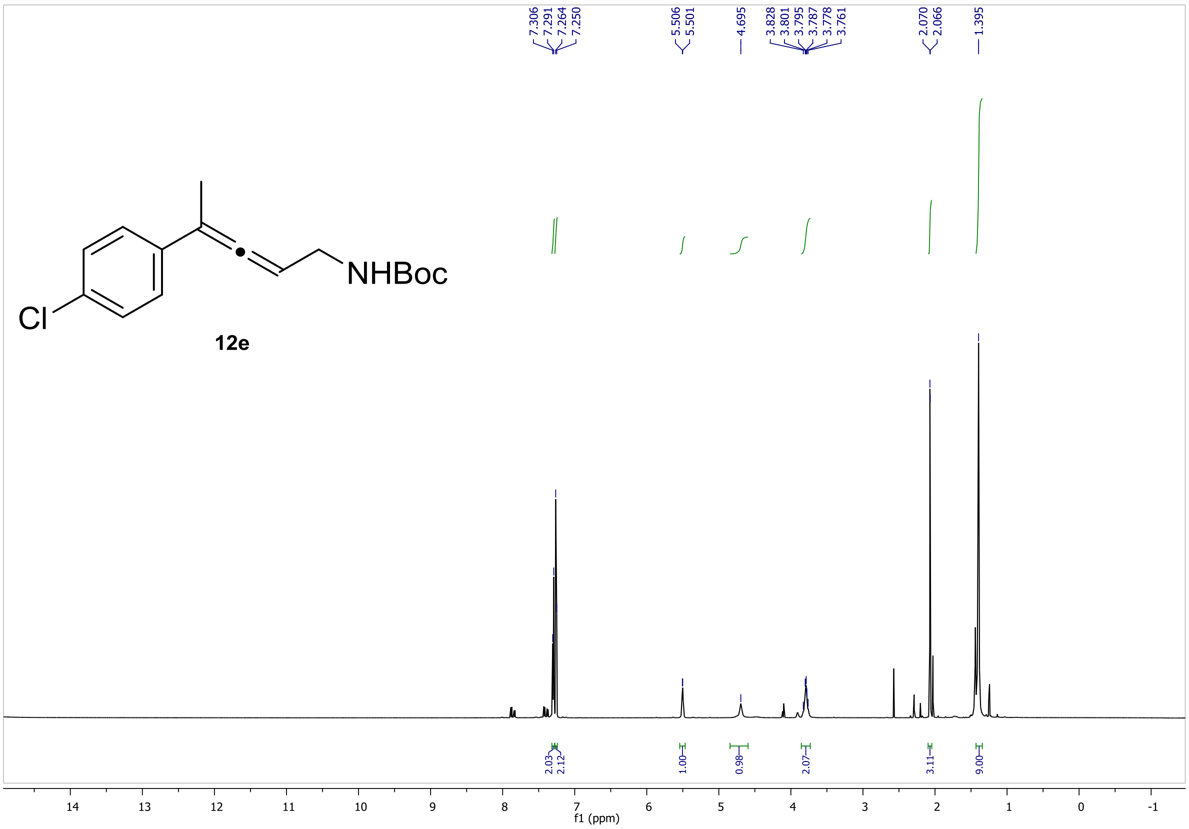

^{13}C NMR, 150 MHz, CDCl$_3$:

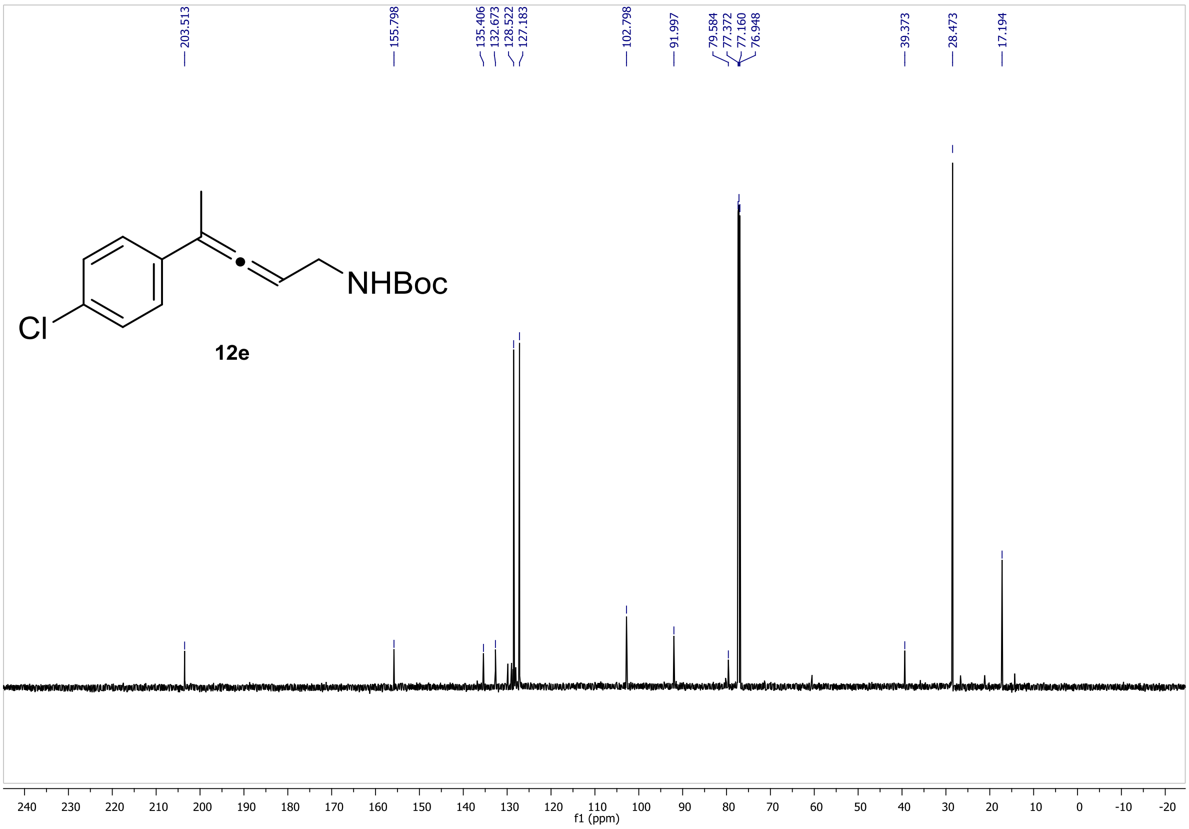

1-chloro-4-(4-phenylbuta-2,3-dien-2-yl)benzene (12f):

^{1}H NMR, 600 MHz, $CDCl_3$:

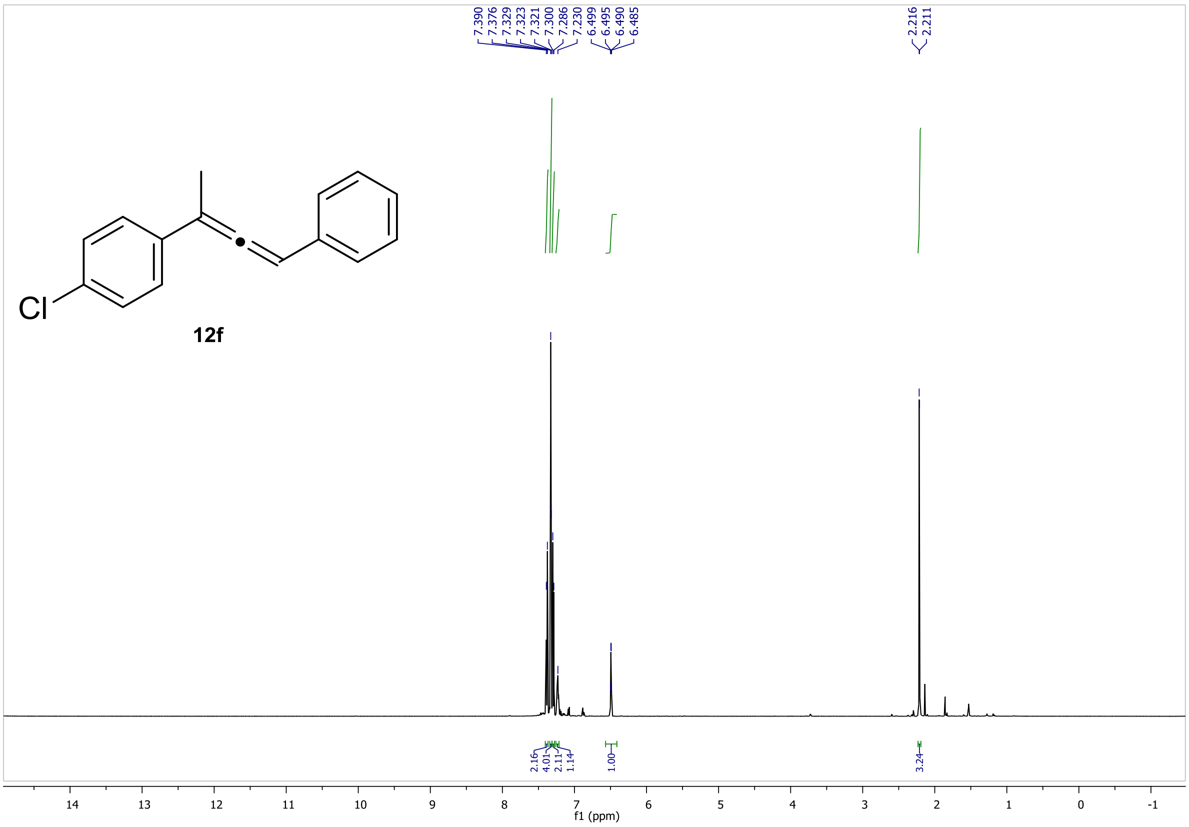

^{13}C NMR, 150 MHz, $CDCl_3$:

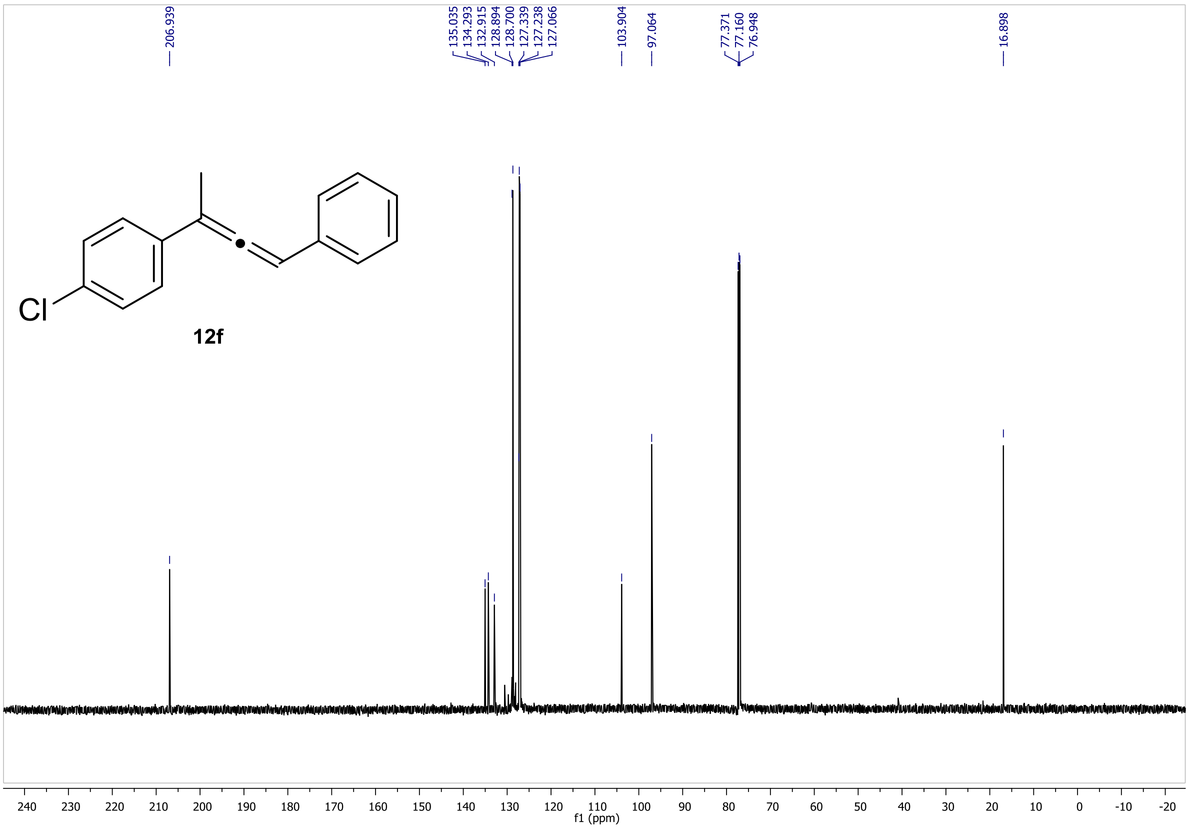

3-(3-(4-chlorophenyl)buta-1,2-dien-1-yl)thiophene (12g):

^{1}H NMR, 600 MHz, $CDCl_3$:

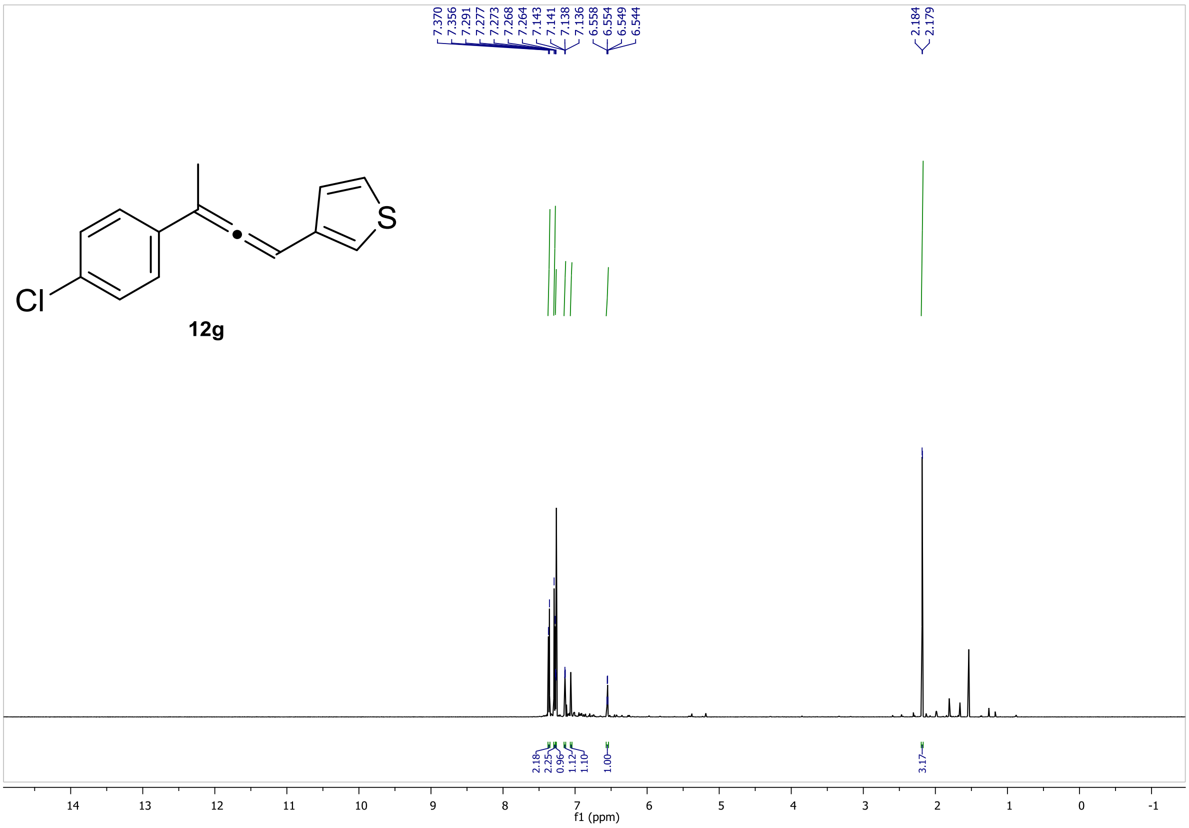

^{13}C NMR, 150 MHz, $CDCl_3$:

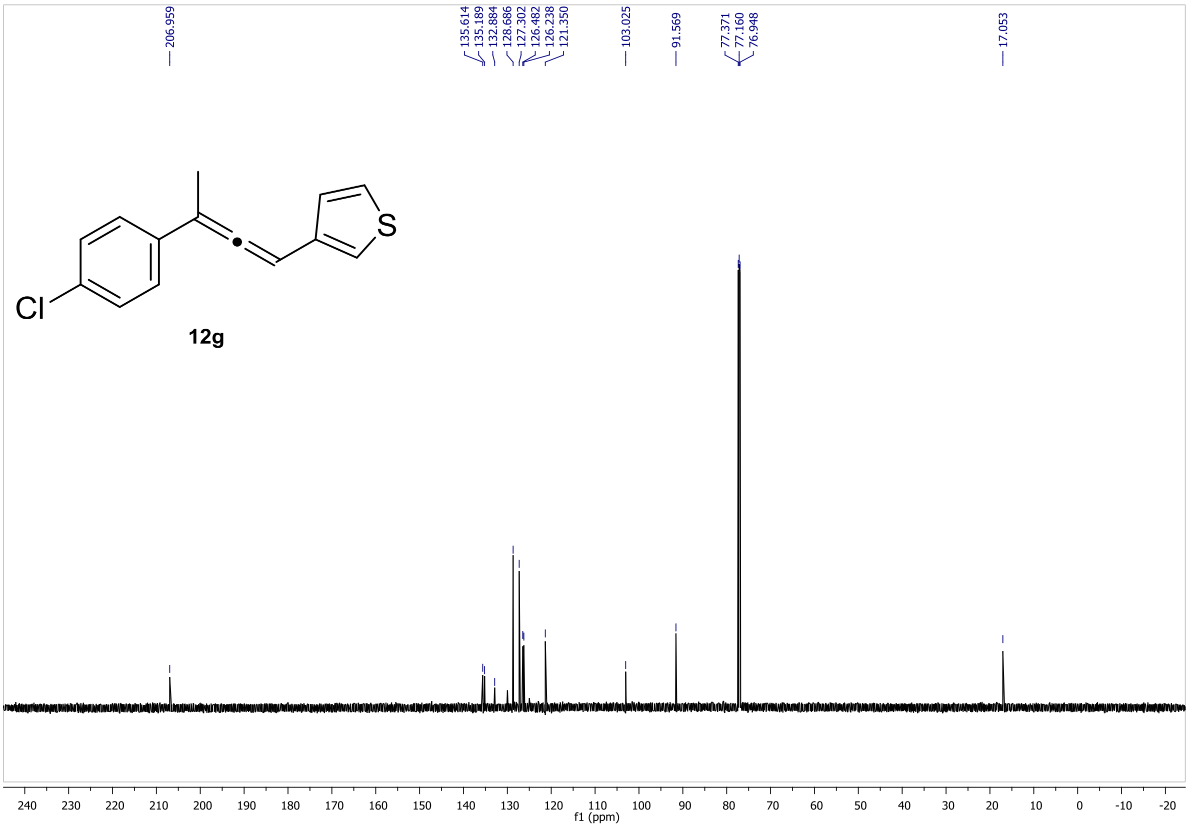

(3-cyclopropylpropa-1,2-diene-1,1-diyl)dibenzene (12h):

^{1}H NMR, 600 MHz, $CDCl_3$:

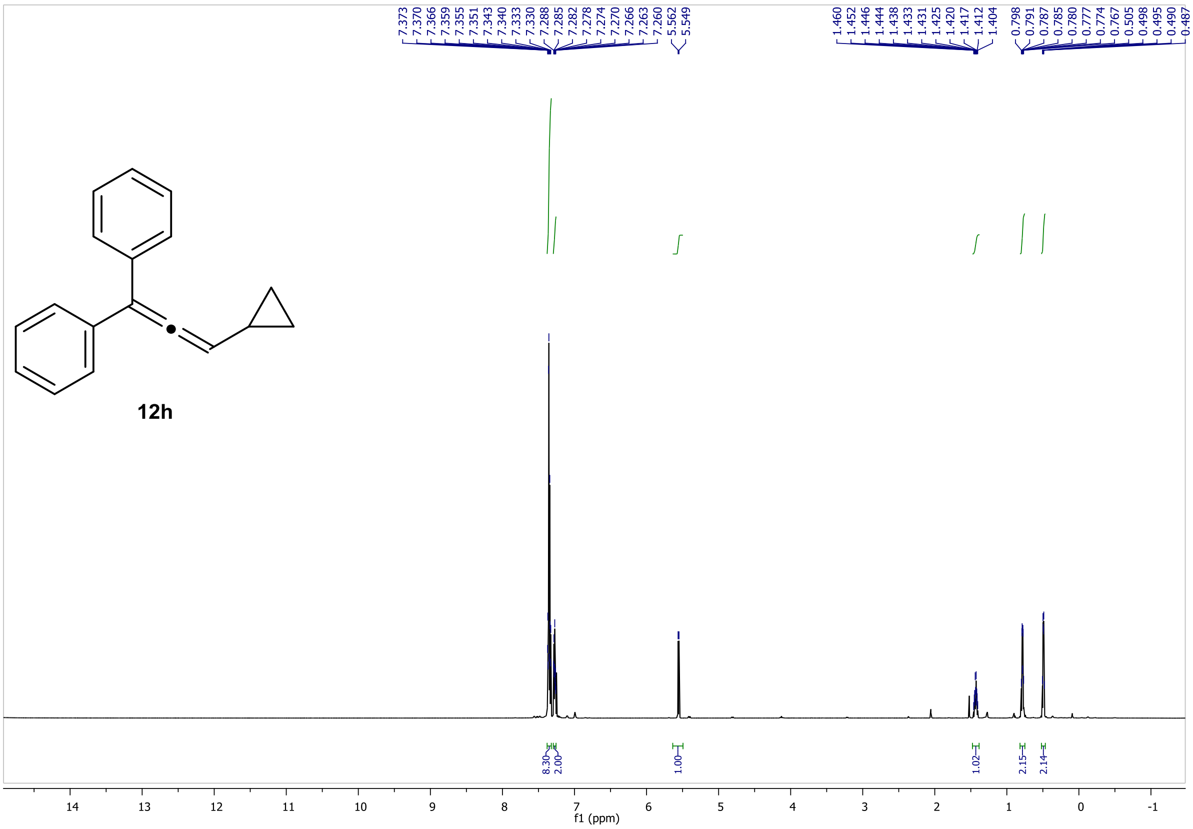

^{13}C NMR, 150 MHz, $CDCl_3$:

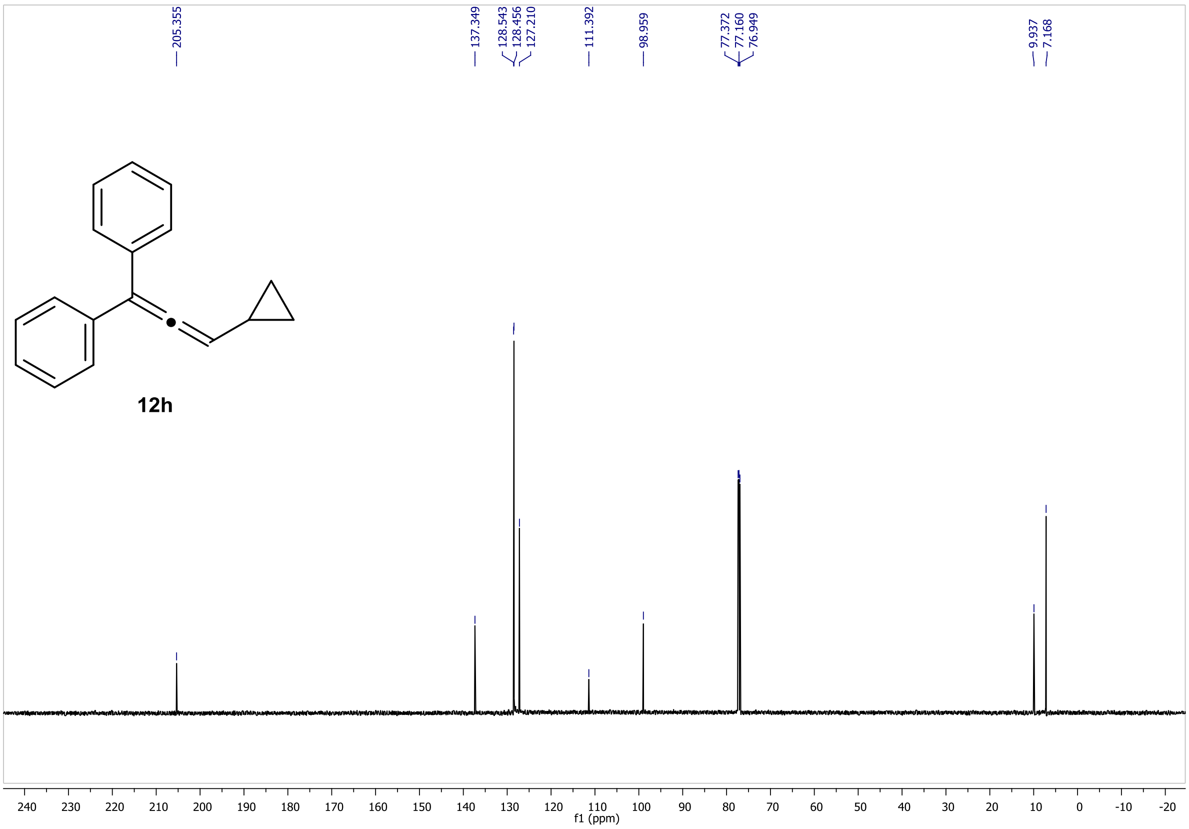

***tert*-butyl (4,4-diphenylbuta-2,3-dien-1-yl)carbamate (12i):**

^{1}H NMR, 600 MHz, CDCl$_3$:

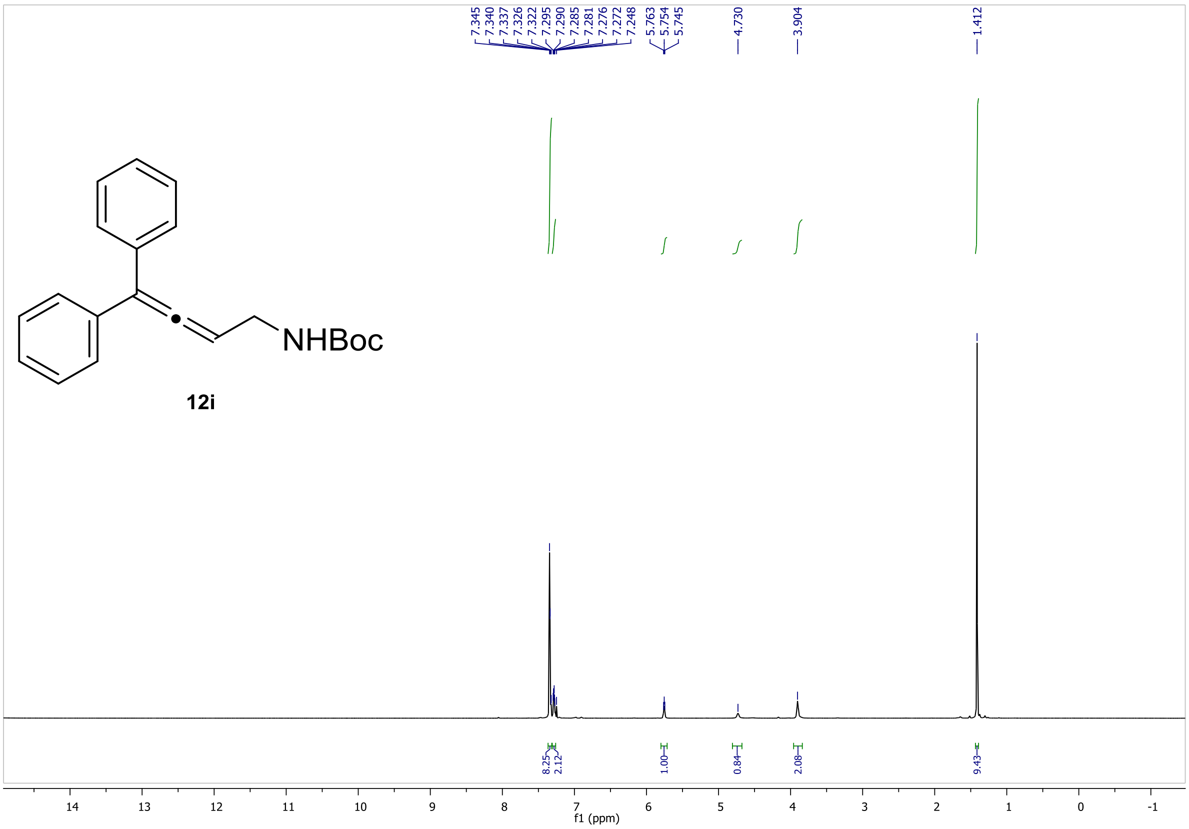

^{13}C NMR, 150 MHz, CDCl$_3$:

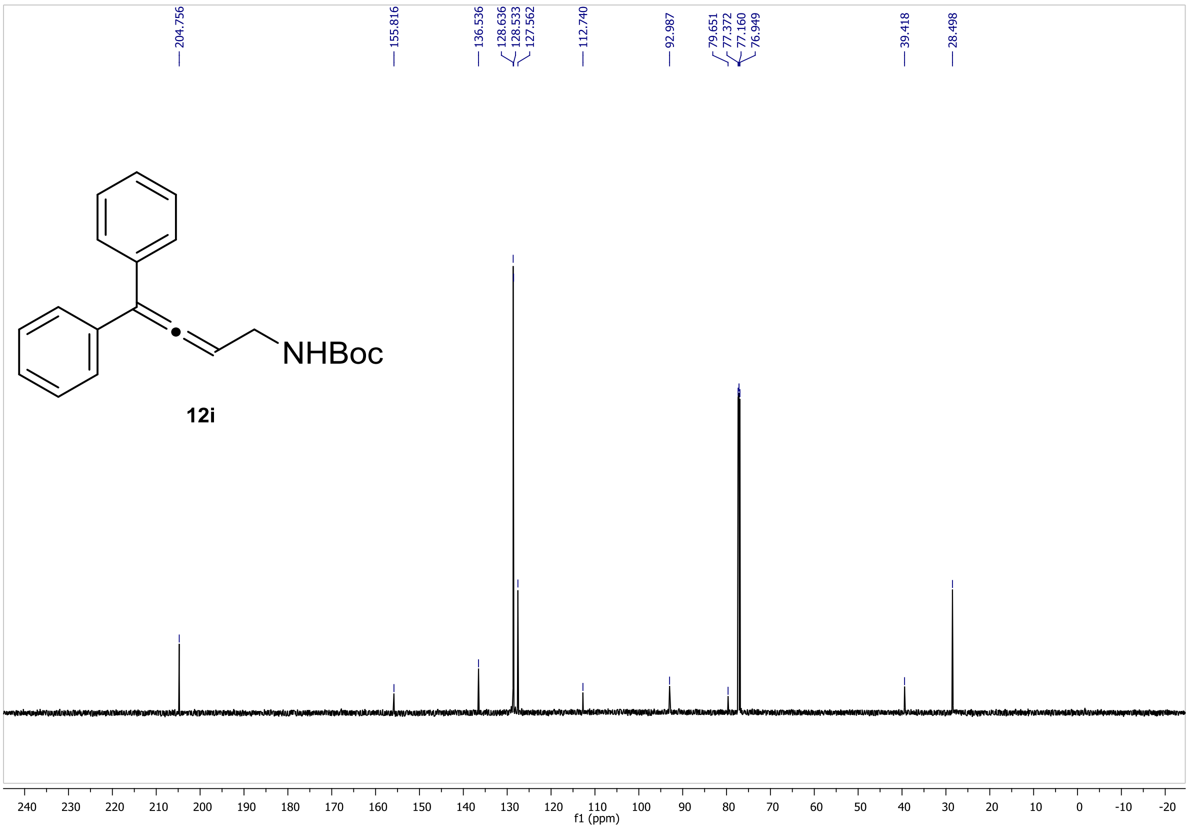

4,4-diphenylbuta-2,3-dien-1-ol (12j):

^{1}H NMR, 600 MHz, $CDCl_3$:

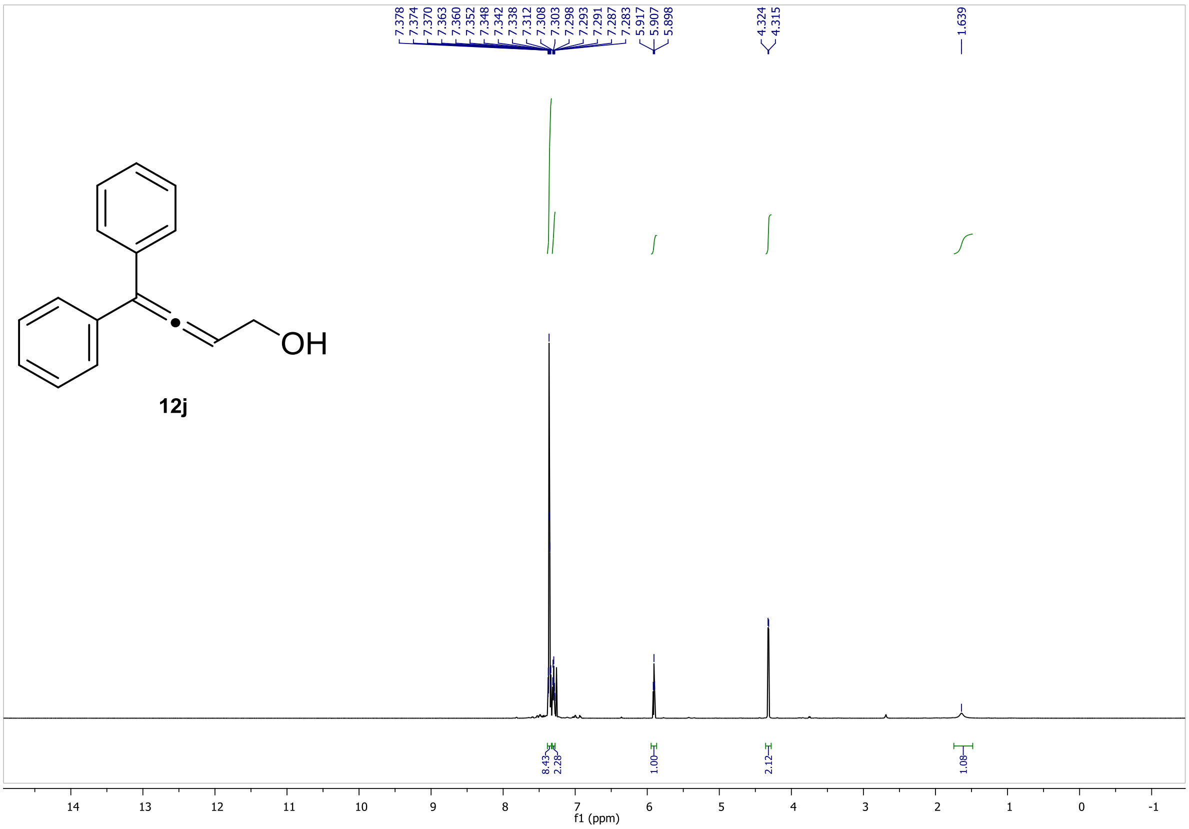

^{13}C NMR, 150 MHz, $CDCl_3$:

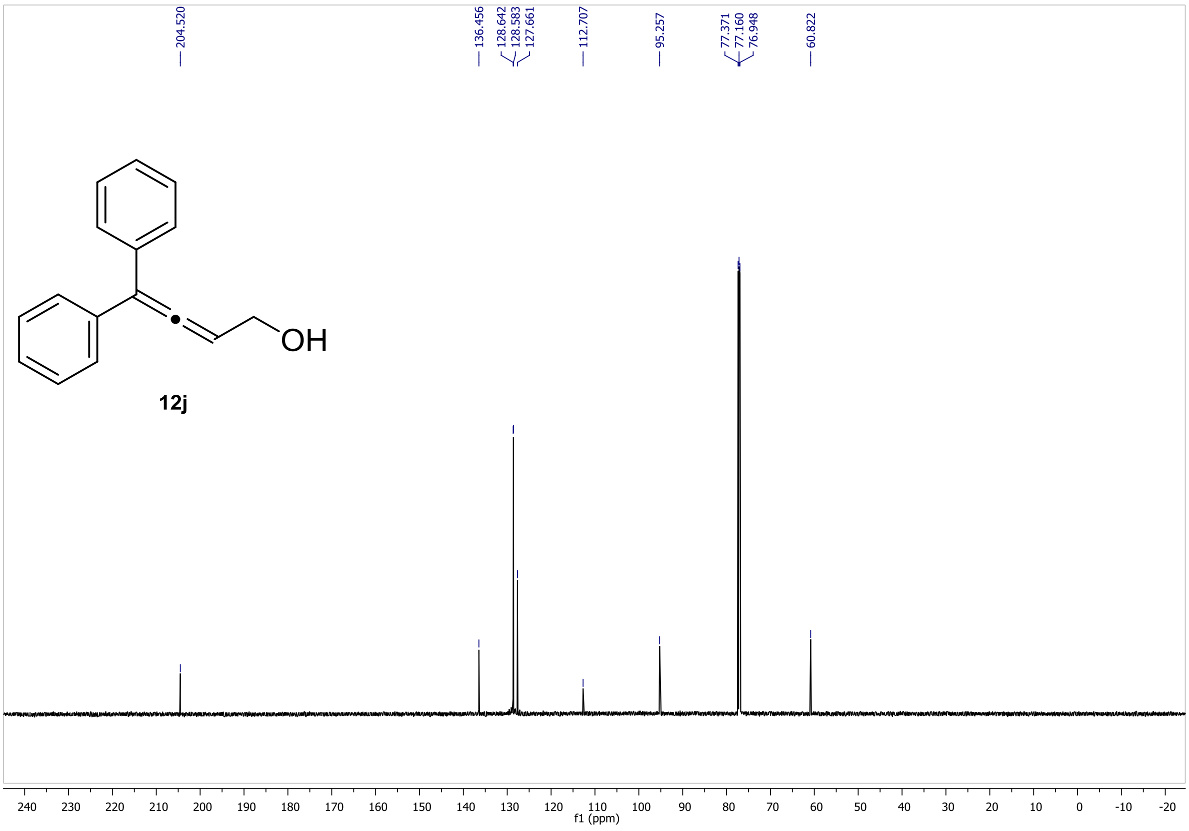

***tert*-butyl (4-(4-(trifluoromethyl)phenyl)octa-2,3,7-trien-1-yl)carbamate (12k):**

^{1}H NMR, 600 MHz, $CDCl_3$:

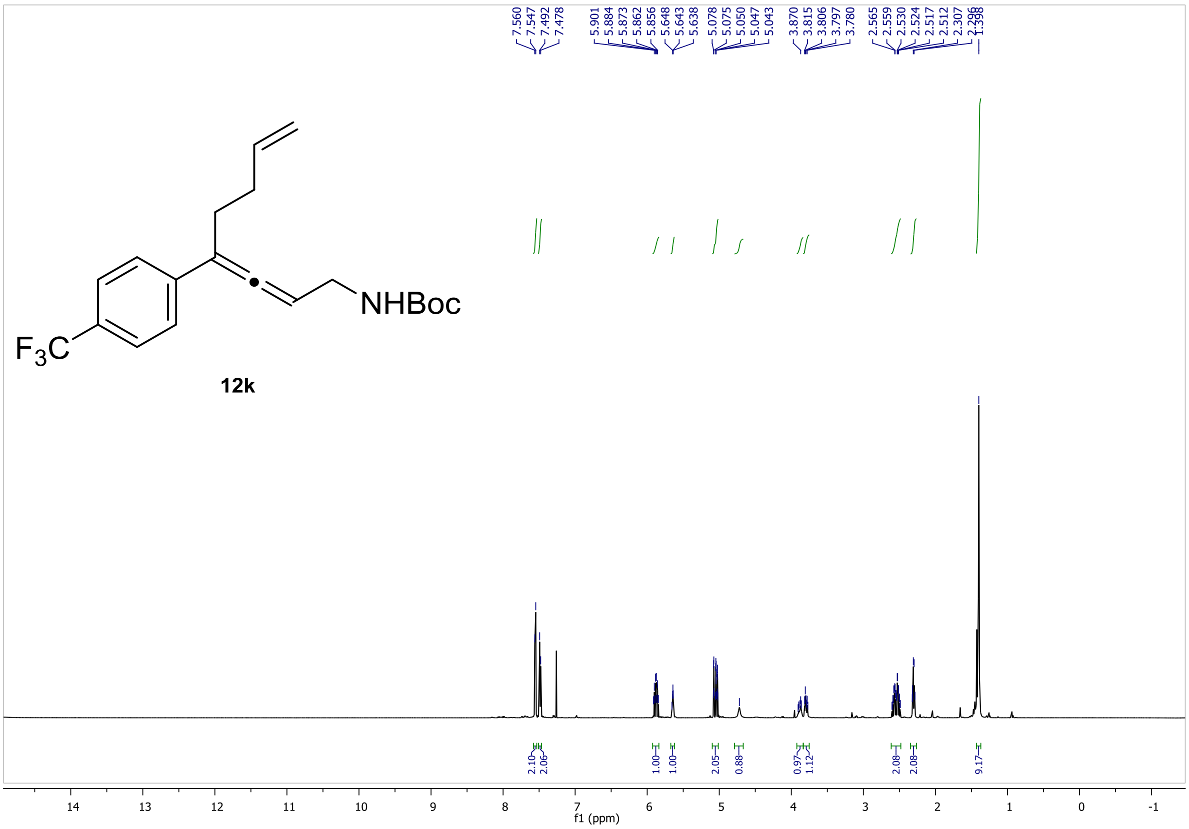

^{13}C NMR, 150 MHz, $CDCl_3$:

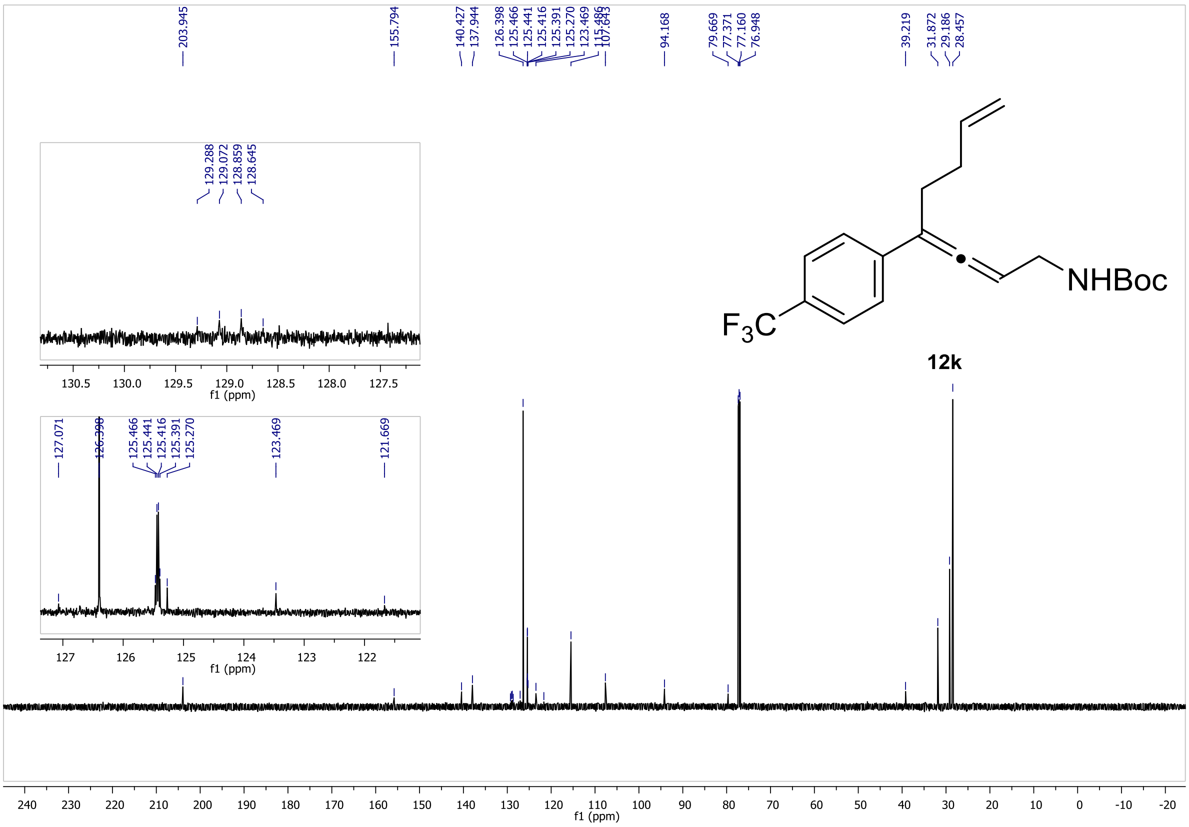

^{19}F NMR, 376 MHz, $CDCl_3$:

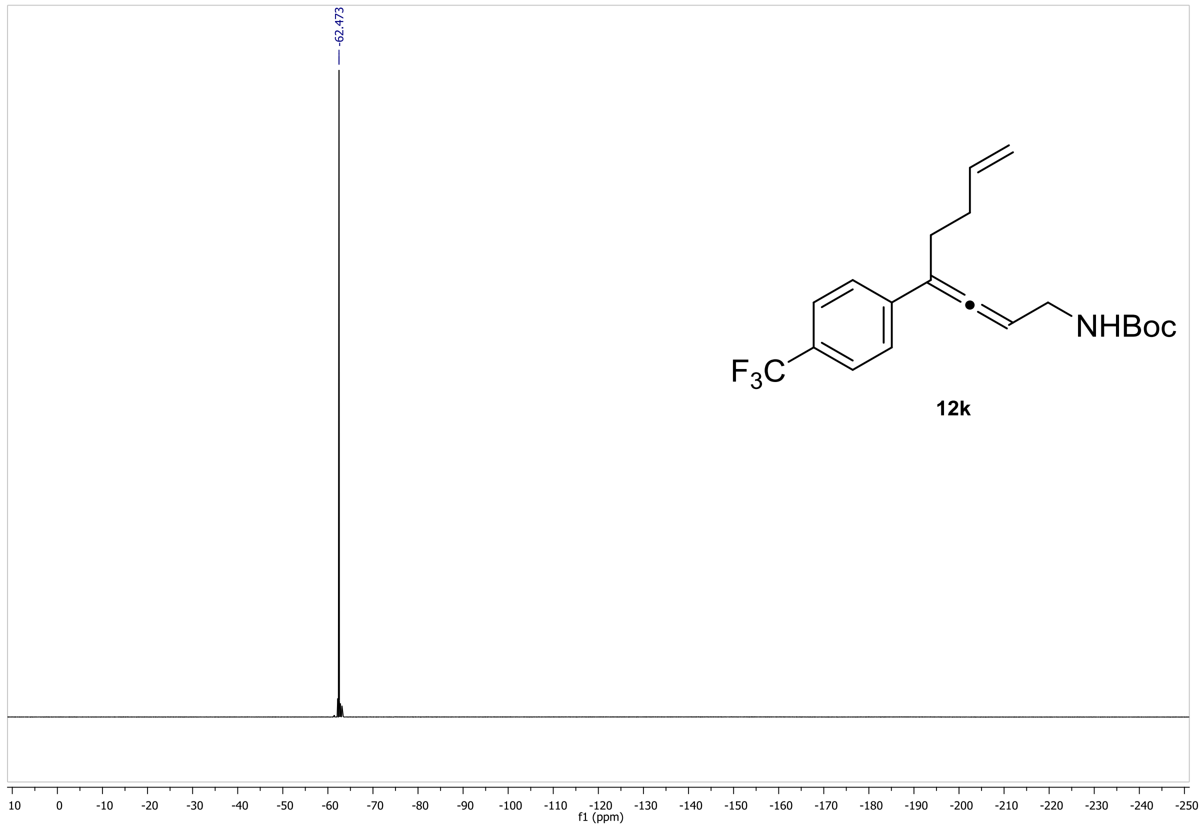

2-(8-bromo-4-(4-methoxyphenyl)octa-4,5-dien-1-yl)-5,5-dimethyl-1,3-dioxane (12l):

^{1}H NMR, 400 MHz, $CDCl_3$:

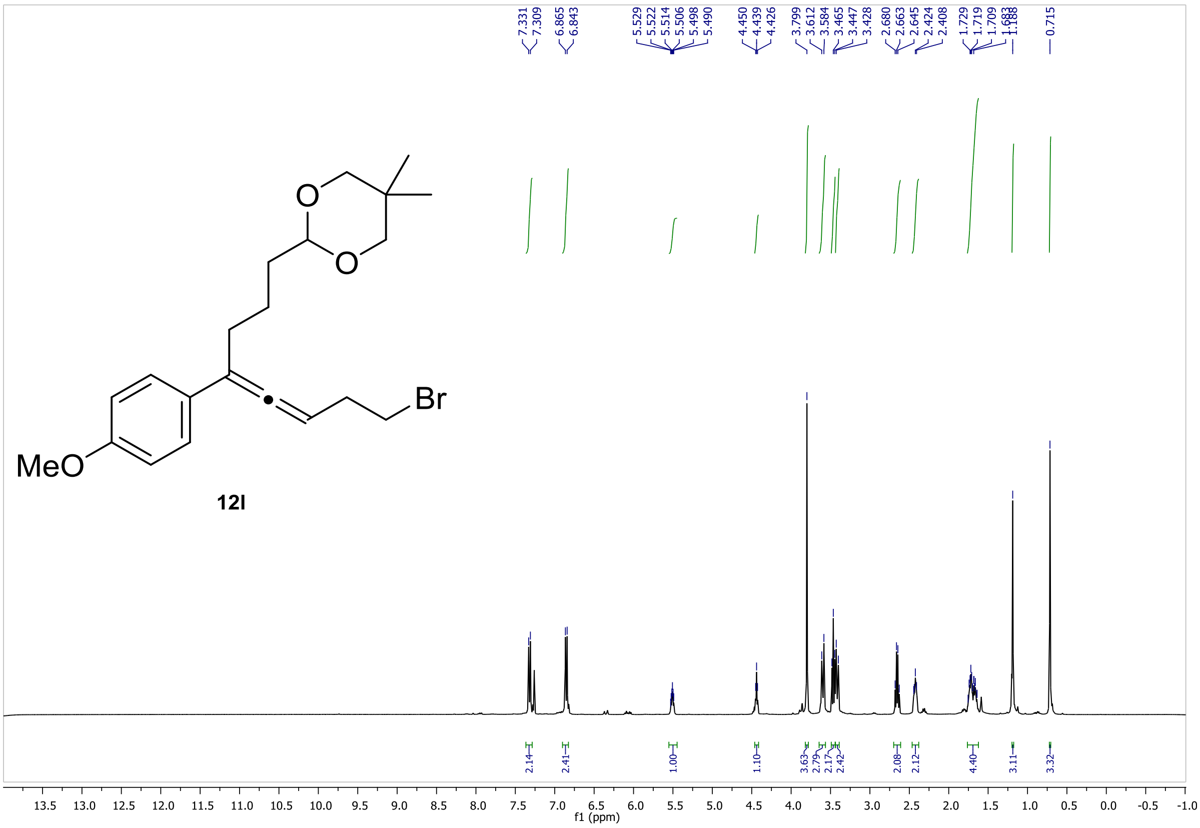

^{13}C NMR, 100 MHz, $CDCl_3$:

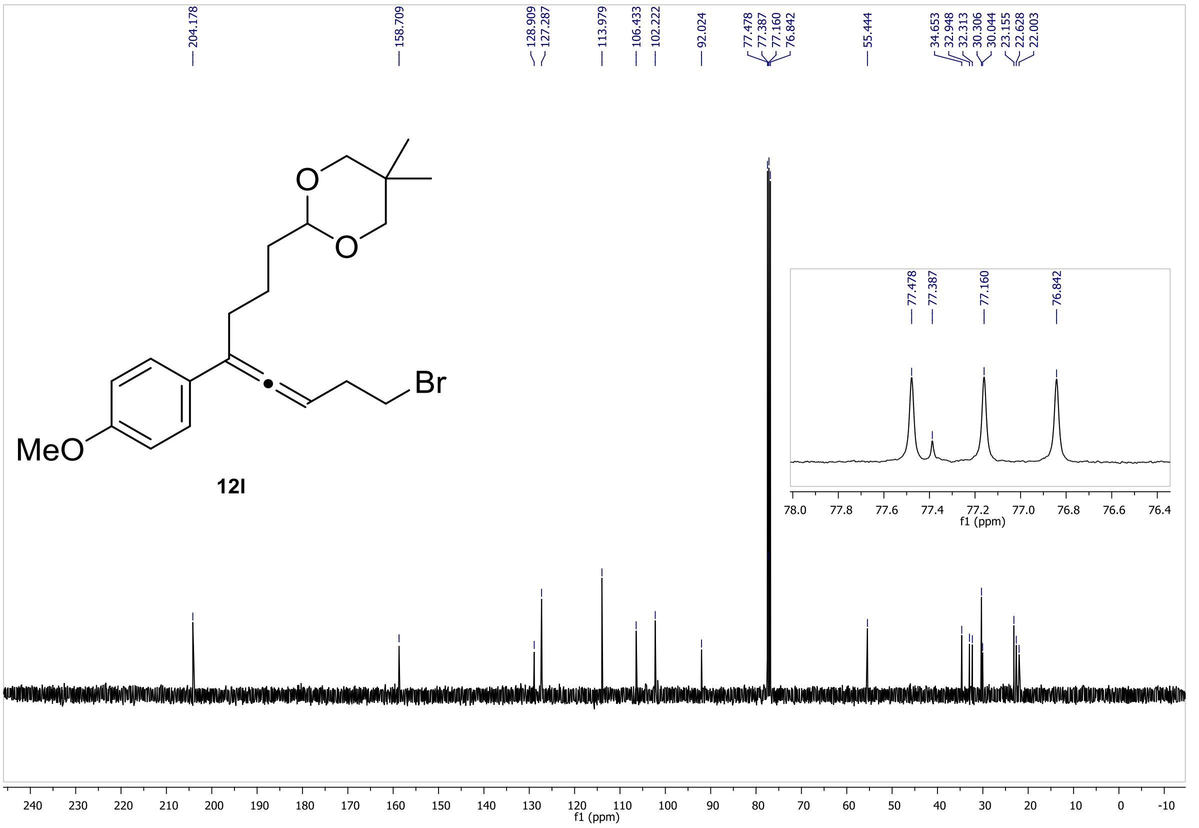

1-chloro-4-(3-cyclopropylpropa-1,2-dien-1-yl-3-*d*)benzene ([D]-4j):

^{1}H NMR, 600 MHz, $CDCl_3$:

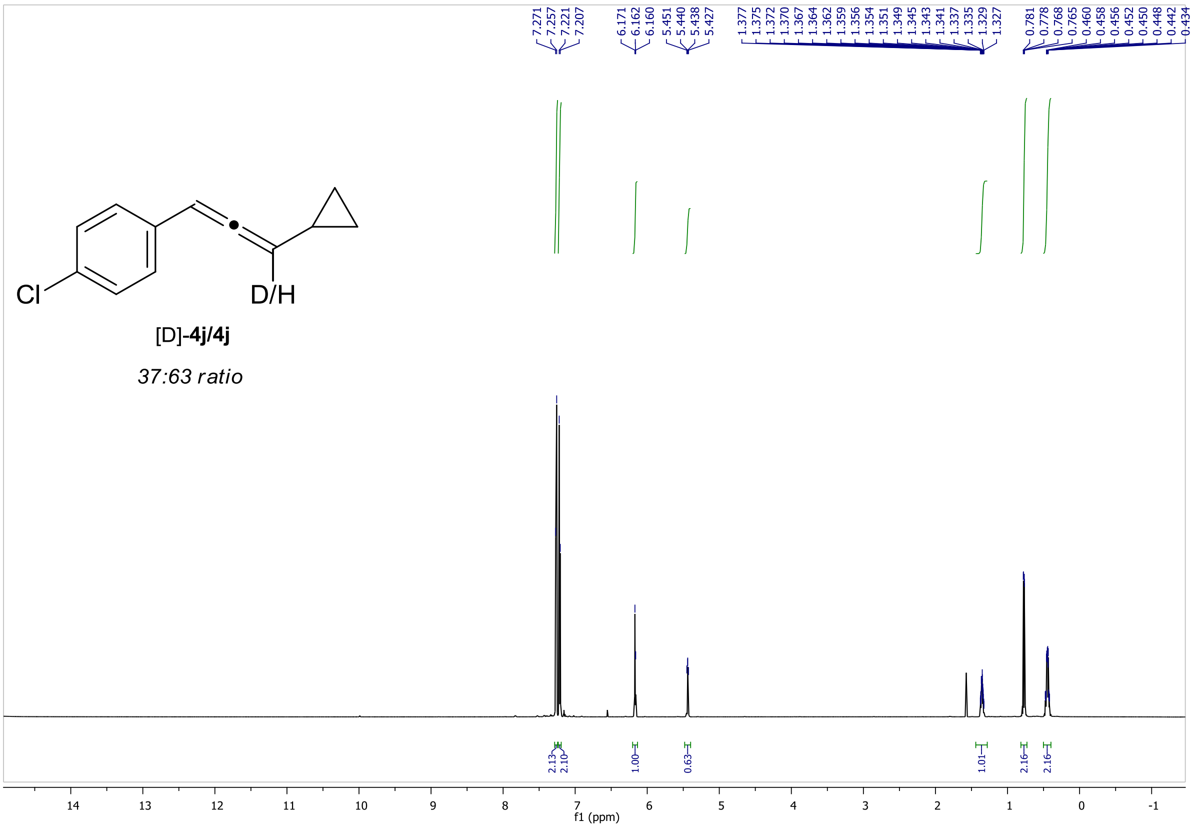

^{13}C NMR, 150 MHz, $CDCl_3$:

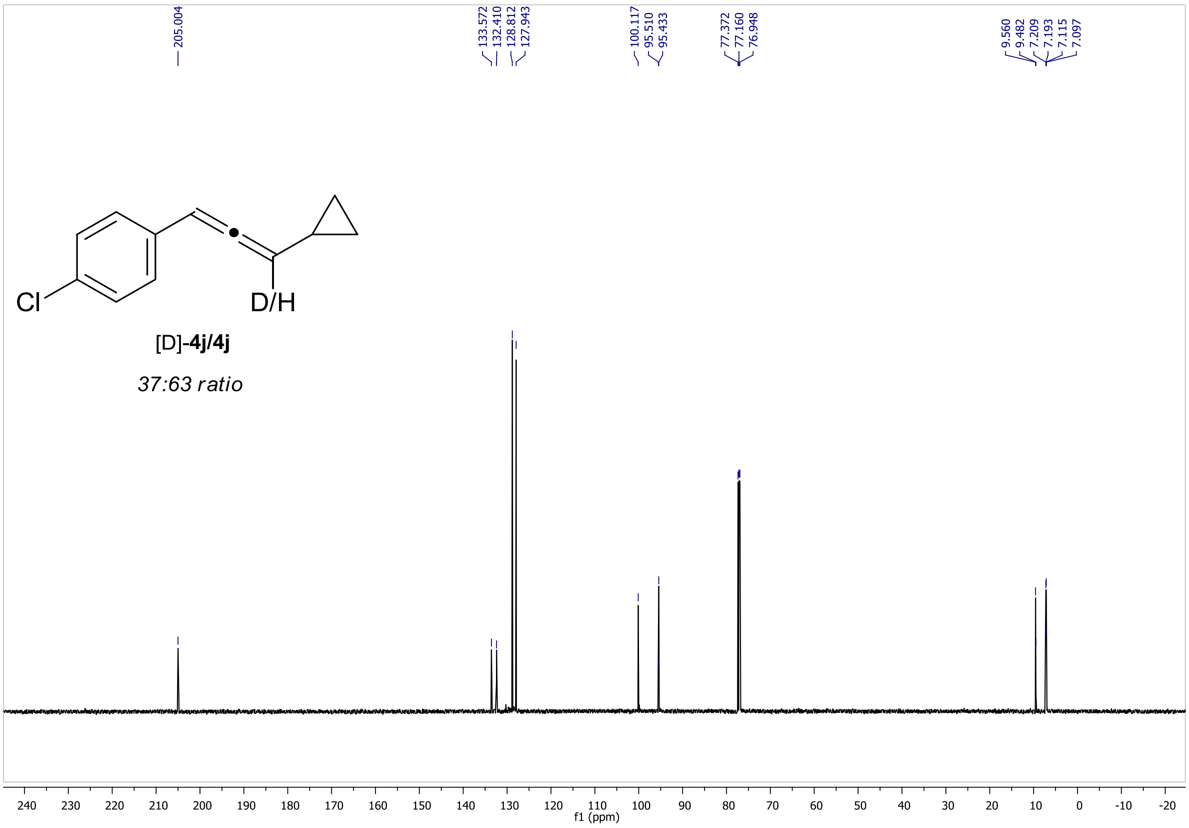

6. References

[1] http://www.uniqsis.com/

[2] http://uk.mt.com/gb/en/home/products/L1_AutochemProducts/ReactIR/flow-ir-chemis.html

[3] V. Z. Shirinian, L. I. Belen'kii, M. M. Krayushkin, *Russ. Chem. Bull.* **1999**, *48*, 2171-2172.

[4] A. V. Shastin, V. N. Korotchenko, V. G. Nenajdenko, E. S. Balenkova, *Tetrahedron* **2000**, *56*, 6557-6563.

[5] M. S. Gordon, S. A. Sojka, *J. Org. Chem.* **1984**, *49*, 97-100.

[6] V. G. Nenajdenko, V. N. Korotchenko, A. V. Shastin, E. S. Balenkova, K. Brinner, J. A. Ellman, *Org. Synth.* **2005**, *82*, 93-98.

[7] V. Gudla, R. Balamurugan, *J. Org. Chem.* **2011**, *76*, 9919-9933.

[8] Y. Ishii, R. Fujimoto, M. Mikami, S. Murakami, Y. Miki, Y. Furukawa, *Org. Process Res. Dev.* **2007**, *11*, 609-615.

[9] http://www.knauer.net/en/downloads/pumps.html

[10] D. A. Mundal, K. E. Lutz, R. J. Thomson, *J. Am. Chem. Soc.* **2012**, *134*, 5782-5785.

[11] P. Warner, R. Sutherland, *J. Org. Chem.* **1992**, *57*, 6294-6300.

[12] Q. Xiao, Y. Xia, H. Li, Y. Zhang, J. Wang, *Angew. Chem. Int. Ed.* **2011**, *50*, 1114-1117.

[13] L.-P. Liu, M. Shi, *Chem. Commun.* **2004**, 2878-2879.
